# CBFA: phenotype prediction integrating metabolic models with constraints derived from experimental data

**DOI:** 10.1186/s12918-014-0123-1

**Published:** 2014-12-03

**Authors:** Rafael Carreira, Pedro Evangelista, Paulo Maia, Paulo Vilaça, Marcellinus Pont, Jean-François Tomb, Isabel Rocha, Miguel Rocha

**Affiliations:** Centre of Biological Engineering, University of Minho, Campus de Gualtar, Braga, Portugal; CCTC, University of Minho, Campus de Gualtar, Braga, Portugal; E.I. DuPont De Nemours & Co., Inc, Wilmington, DE USA; SilicoLife, Lda, Rua do Canastreiro 15, Braga, Portugal

**Keywords:** Constraint-based modeling, Metabolic Flux analysis, Metabolic engineering, Open-source software

## Abstract

**Background:**

Flux analysis methods lie at the core of Metabolic Engineering (ME), providing methods for phenotype simulation that allow the determination of flux distributions under different conditions. Although many constraint-based modeling software tools have been developed and published, none provides a free user-friendly application that makes available the full portfolio of flux analysis methods.

**Results:**

This work presents *Constraint-based Flux Analysis* (CBFA), an open-source software application for flux analysis in metabolic models that implements several methods for phenotype prediction, allowing users to define constraints associated with measured fluxes and/or flux ratios, together with environmental conditions (e.g. media) and reaction/gene knockouts. CBFA identifies the set of applicable methods based on the constraints defined from user inputs, encompassing algebraic and constraint-based simulation methods. The integration of CBFA within the OptFlux framework for ME enables the utilization of different model formats and standards and the integration with complementary methods for phenotype simulation and visualization of results.

**Conclusions:**

A general-purpose and flexible application is proposed that is independent of the origin of the constraints defined for a given simulation. The aim is to provide a simple to use software tool focused on the application of several flux prediction methods.

**Electronic supplementary material:**

The online version of this article (doi:10.1186/s12918-014-0123-1) contains supplementary material, which is available to authorized users.

## Background

Over the past 20–30 years, there has been a substantial increase in the production of materials through the use of cell factories. Metabolic engineering (ME) [1] deals with the analysis and design of metabolic systems towards particular goals, such as increasing the production of useful compounds. The field has received increased attention, due to the growing adoption of industrial biotechnology for producing pharmaceuticals, food ingredients and fine chemicals, among others [2]. The emergence of high-throughput tools for genome sequencing [3], gene expression, protein and metabolome analysis [4], has enabled a better understanding of cell metabolism and, consequently, empowered rational strain development approaches. However, a deeper understanding of the physiological state of cells can be obtained from metabolic flux profiles [5] and, therefore, the measurement of metabolic fluxes and the understanding of their control within metabolic systems are at the core of ME.

Current biochemical knowledge and the information collected from the annotation of genome sequencing projects allowed the development of genome-scale metabolic models (GSMMs), supporting the simulation of the metabolic phenotypes. Although the process is only semi-automatic, a large number of GSMMs have been reconstructed for different organisms [6].

Through the use of a metabolic model, taking into account stoichiometry, reaction reversibility, and quasi-steady-state assumptions, linear constraints over the values of intracellular fluxes can be established. Environmental conditions (e.g. media) are added in the form of constraints over uptake fluxes, while reaction deletions can be defined setting the respective fluxes to zero. However, in the context of genome-scale models, the majority of these systems are underdetermined [7], meaning that the number of constraints is not enough to algebraically solve the system for unknown fluxes. Thus, simulation approaches based on linear/quadratic programming (LP/QP) optimization methods are used to calculate flux values. This is the case with the well-known Flux Balance Analysis (FBA) method [8], where an optimization problem is formulated to optimize a defined objective function, typically the maximization of growth rate as defined by an artificial biomass flux [9].

However, the configuration of a proper objective function is not straightforward [10]. Moreover, LP based approaches can have multiple solutions, i.e. different flux distributions that satisfy all the constraints and have the same optimal value for the objective function [11]. Additional limitations arise from the reliance on balances for cofactors, such as NADH and NADPH [12] and from the presence of reversible reactions and futile cycles [13]. Some of these problems can be attenuated by experimental measurements or previous knowledge of certain metabolic fluxes, but these are rarely able to turn the system into a determined one, to allow the unique determination of all fluxes in the model.

Metabolic flux analysis (MFA) methods are, thus, based on the use of metabolic models and experimental data (fluxomics). When available, data of measured fluxes (both exchange and internal fluxes) and known flux ratios [14,15] can be added to the metabolic model in the form of additional constraints, reducing the solution space, eventually leading to a determined (or over-determined) system. These data are usually obtained from the measurements of exchange fluxes, such as the rates of formation and consumption of compounds or from more sophisticated procedures that include feeding of labelled substrates [5].

Several software applications have been put forward providing methods for flux quantification based on experimental data [16]. These typically work over data from ^13^C labelled substrate experiments [17-20].

Numerous tools for general-purpose constraint-based analysis have been developed in the last few years. In Additional file 1: Table S1 in supplementary material, the main features of these tools are summarized. All these tools offer the possibility to perform FBA, however few provide flexible and easy-to-use tools for defining additional constraints as those coming from measured or otherwise known fluxes or flux ratios. For example, in this GSMMs’ era, it is important not only to provide users with the capacity to compute flux distributions from experimental data for determined systems, but also to evaluate the impact of the measurements for systems that, although remaining underdetermined, see their solution space reduced. Moreover, it is important to be able to combine different types of experimental data or other available knowledge, such as flux measurements or ratios for intracellular fluxes with exchange fluxes or to easily perform these calculations for both wild-type and mutant strains.

Few tools exploit the various derivations of FBA that enable the application of tailored approaches to specific problems. Although COBRA [19] is the tool that contains the most relevant features, it requires the commercial software Matlab. CellNetAnalyser [21], the successor of FluxAnalyzer [16], provides key features for MFA, such as the classification of models according to determinacy and redundancy. However, it is also built over Matlab and is itself a commercial tool, although it allows an academic license. A few software tools, like MetaFluxNet and SBRT [22], execute matrix operations to calculate flux distributions without the application of optimization methods. Also, importing metabolic flux ratios to constrain MFA methods is a feature not supported by existing tools, although SBRT and COBRA accept the introduction of linear combinations of reactions to constrain the systems.

In this work, *CBFA – Constraint-based Flux Analysis*, a general-purpose and flexible application is proposed that is independent of the type of data available and their processing. *CBFA* is able to work with regular constraint-based models, without information about atom transitions between precursors and products in the model reactions (as required by other tools). Independence from carbon atom mapping facilitates user interaction, since this kind of information is not easily compiled for genome-scale models, though efforts to accomplish such task have been reported [23,24]. In *CBFA*, input data are not fluxomics measurements generated by analytical techniques, such as Mass Spectrometry (MS) or Nuclear Magnetic Resonance (NMR), rather, input data are known or measured metabolic flux ratios, which can be easily used to reflect the portions of flux that generate a certain metabolite originating from different pathways [14,15] and that have already been used in the context of genome-scale models [25]. Other data, such as known or measured exchange or internal fluxes can also be directly inputted. The aim is to provide a simple to use software tool focused on the straightforward application of several state-of-the-art flux analysis methods and the simulation of the behaviour of wild type or mutant strains. The development of tools to generate these ratios from fluxomics data (GC-MS) is addressed in a complementary software, whose publication is currently under preparation.

The main features of this tool are the following:

- Open-source – it allows all users to use the tool freely and invites contribution of other researchers by providing the source code to the community;
- User-friendly – facilitates its use by users with no/little background in modelling/programming;
- Simple – needs only a few steps to perform flux analysis methods and obtain flux distributions, simplifying the use of experimental information in the simulations;
- Modular – as it is incorporated in the Optflux software [26], it follows a plug-in based architecture, facilitating the addition of specific features;
- Compatible with standards –compatibility with the Systems Biology Markup Language (SBML) [27], the Mathematical Markup Language (MathML) [28] and several layout standards for visualization (SBGN-ML, XGMML, SBML Layout) rendering interoperability with other tools easier.

In its current version, *CBFA* accommodates several tools and algorithms that have been developed for the manipulation of metabolic models:

- methods for phenotype simulation, such as FBA and its variant Parsimonious Enzyme Usage FBA (pFBA) [29];
- a method to minimize the error between calculated and measured fluxes, through a quadratic programming formulation;
- methods for system characterization:
  - ○ through the calculation of tight bounds of fluxes in the model;
  - ○ through the analysis of the null space of the stoichiometric matrix.
- a method for robustness analysis of a configured objective function;
- methods to calculate fluxes when the non-measured fluxes can be calculated from the input constraints, and exploiting redundancies when additional data are available;
- a suitable model visualization tool to facilitate the interpretation of results.

Additional file 1: Table S1 highlights some of the features of this tool, when compared to some of the applications mentioned above. It also lists, in a different tab, the novel features of this tool in the framework of the OptFlux application.

The main concepts used in the development and its main functionalities are presented in the next sections.

## Implementation

The tool is fully implemented in the Java language, and thus available for all platforms. The only non-Java parts consist of the linear/quadratic programming solvers, where several interfaces are implemented such as the *GNU Linear Programming Kit* [30] and *IBM ILOG CPLEX*.

In order to explore several features for model handling and visualization, and to enable the interaction with different tools, *CBFA* was built as a new plugin for *Optflux. Optflux* is an open-source and modular ME software platform with a plug-in based architecture. *Optflux* and *CBFA* follow the model-view-controller (MVC) design pattern, incorporating three well-defined concepts: *operations*, *datatypes* and *datatype views*. This design pattern allows the combination and re-use of different units of work and facilitates the continuous development of new features.

*CBFA* is a user-friendly software, with a graphical user interface (GUI) layer to allow users to call and visualize the software features and results, where the presentation layer is well separated from the business and application layers. Thus, all its features can be used by other software platforms through a well-defined programming interface without the dependency on the GUI input interactions.

## Results and discussion

**Figure 1:**
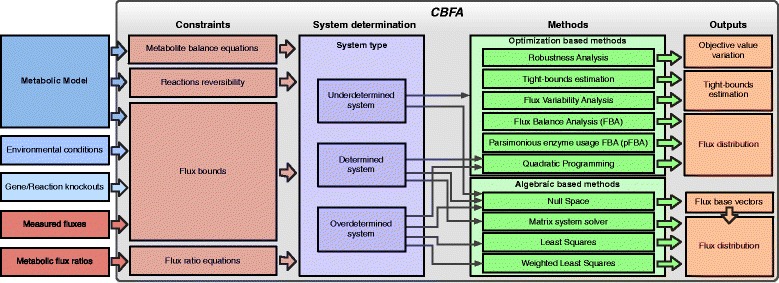
**Overview of the application: on the left side, the inputs are represented; the centre box contains the different functional blocks of the application: the types of constraints, the determination of the system type and the supported methods; the right boxes represent the outputs of the methods.**

An overview of the overall workflow implemented in *CBFA* is provided in Figure 1, where the different inputs, the generated constraints, the system types, the supported methods and their outputs are shown. In this section, the main steps and alternatives offered by the pipeline to execute the flux analysis methods are briefly explained. The detailed description of the implemented methods and complete mathematical formulations are provided as supplementary material (Additional file 2), also available on the project’s website. Additionally, a Beginner’s tutorial with illustrations for all the steps needed to perform the software tasks is available to help first-time users (Additional file 3 and online documentation on the site).

### System configuration

Starting with a metabolic model that can be loaded in different formats (e.g. SBML or CSV files), the user can configure inputs to flux analysis methods, including environmental and genetic conditions (gene/reaction deletions), measured or otherwise known fluxes and/or flux ratios given as expressions in the form:

$$ \frac{{\displaystyle {\sum}_{i=1}^n{\kappa}_i{\upsilon}_i}}{{\displaystyle {\sum}_{j=1}^n{\kappa}_j{\upsilon}_j}}=\tau $$

where κ_i_, κ_j_ ∈ R and τ ∈ R\{0} are user-defined real numbers and v_i_ and v_j_ are fluxes. This allows the definition of constraints of different origins, e.g. coming from calculations over experimental data, for example from ^13^C experiments, or deriving from existing knowledge about a biological system. Note that the specific calculations to reach flux ratios from experimental data in different formats are not included in this software.

Given this information, degrees of freedom of the system are calculated from the properties of the original stoichiometric matrix of the model, the defined knockouts, the experimental values defined for some fluxes, and the provided flux ratios. The accurate number of degrees of freedom is obtained by the difference between the number of unknown variables (fluxes) of the system and the number of linearly independent equations of the system. However, since the purpose of this work is mainly to perform flux analysis tasks upon GSMMs, the typical configurations will lead to large numbers of degrees of freedom. It is not relevant to calculate the exact number of degrees of freedom if this is too high. Therefore, the costly algebraic operations that are performed towards the exact calculation of the degrees of freedom are not always made, rather an approximation is firstly calculated, given by the difference between the unknowns and the equations of the system, without taking into account the linear dependency between them. If this approximation gets close to a threshold, the proper number of degrees of freedom is calculated and presented to the user.

To reduce the degrees of freedom, the user can add new flux measurements, gene/reaction deletions or equality flux ratios. If the type of system changes, the set of available methods is updated accordingly.

### Available methods for Flux analysis

Two approaches are used to determine flux distributions: algebraic or optimization based methods (linear or quadratic programming). Depending on the degrees of freedom of the system, different methods can be applied (Figure 1). If the system is either determined or over-determined it can be algebraically solved and, therefore, fluxes can be calculated. In the latter case, the additional information can be used to calculate fluxes by regular or weighted least squares fitting.

However, with genome-scale models, systems are more commonly underdetermined, even when experimental information is added. Thus, optimization methods are used to obtain flux values, by defining an objective function, while respecting the defined constraints. FBA and pFBA can be selected, maximizing a given objective function (e.g. the biomass flux). An alternative is to use quadratic programming to minimize the difference between the values of a subset of measured fluxes and their calculated values. The resulting formulation is mathematically similar to the minimization of metabolic adjustment (MOMA) [31].

The null space approach can be used to calculate a unique solution to the non- measured fluxes, when the system is either determined or over-determined. It can also be used when the system is underdetermined, but in this case returning the admissible flux space represented by the generating base vectors. These vectors can be used to generate valid flux distributions.

In order to characterize the system, other approaches can also be used like the computation of tight bounds of the system under certain conditions. Here, the lower and the upper admissible bounds for all fluxes, which have neither been measured nor have associated knockouts, are obtained through the application of an optimization-based method that maximizes/minimizes the flux of interest under the same set of constraints. This method is an approach based on Flux Variability Analysis (FVA) [11] where no constraints are defined for the objective function.

Different solutions leading to the same optimal objective value can be achieved by the methods based on LP problems. To characterize the alternative solutions that satisfy the imposed set of constraints and have the same objective value, FVA is again used, this time to calculate the admissible ranges of flux values under optimality [11].

To analyze the changes of the objective function in response to variations of a specific flux, a method for robustness analysis is also available. For a selected set of fluxes, their values in a prior optimization for the given objective are retrieved. After that, each flux is varied in different percentages of its prior value and the effects of these changes on the objective function can be studied [32].

Furthermore, all the aforementioned methods can be used to investigate how a set of knockouts is reflected on the flux distribution. This is accomplished by the configuration of knockouts as additional constraints to the system. These knockouts can be inserted as reactions or as genes by using the gene-reaction rules of the model (when these are available).

The results and configurations used by the methods described above can be exported to a PDF file, including the model details, the method that was used, all the constraints that were selected, and the result of the simulation in terms of objective function and net conversions. Moreover, it reports the bounds that were actually used in the simulations when constraints overlap with each other.

### User interaction

The aim of *CBFA* is to provide the community with a tool to perform flux analysis tasks that is simple, intuitive and with high usability. The interaction with this software is based on three main concepts:

- *Datatypes*: Encapsulate the different types of objects of the application, holding relevant data such as models, flux measurements, flux ratios, simulation results, etc. The application can keep several instances (objects) of the same datatype.
- *Views*: To check the contents of a datatype, panel views are defined that present to the user the most relevant information that it encompasses. Since a single datatype might contain different types of information, it can have multiple views shown as different panels (tabs).
- *Operations*: All the functionalities are invoked in the form of operations, which are units with well-defined sets of inputs and outputs (datatypes). Whenever an operation is called, a dialog is presented to the user to define the set of input objects. The most common scenario is to create a new instance of a certain datatype (e.g. the result of a simulation) or to modify existing instances (e.g. add flux measurements to an existing set).

**Figure 2:**
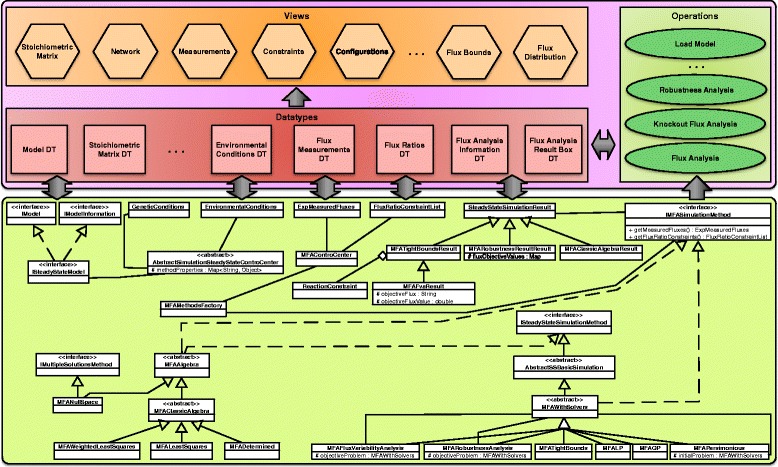
**Functional modules of the CBFA application, including the developed datatypes, views and operations.** The bottom box shows an overview of the classes implemented in the core layer.

**Figure 3:**
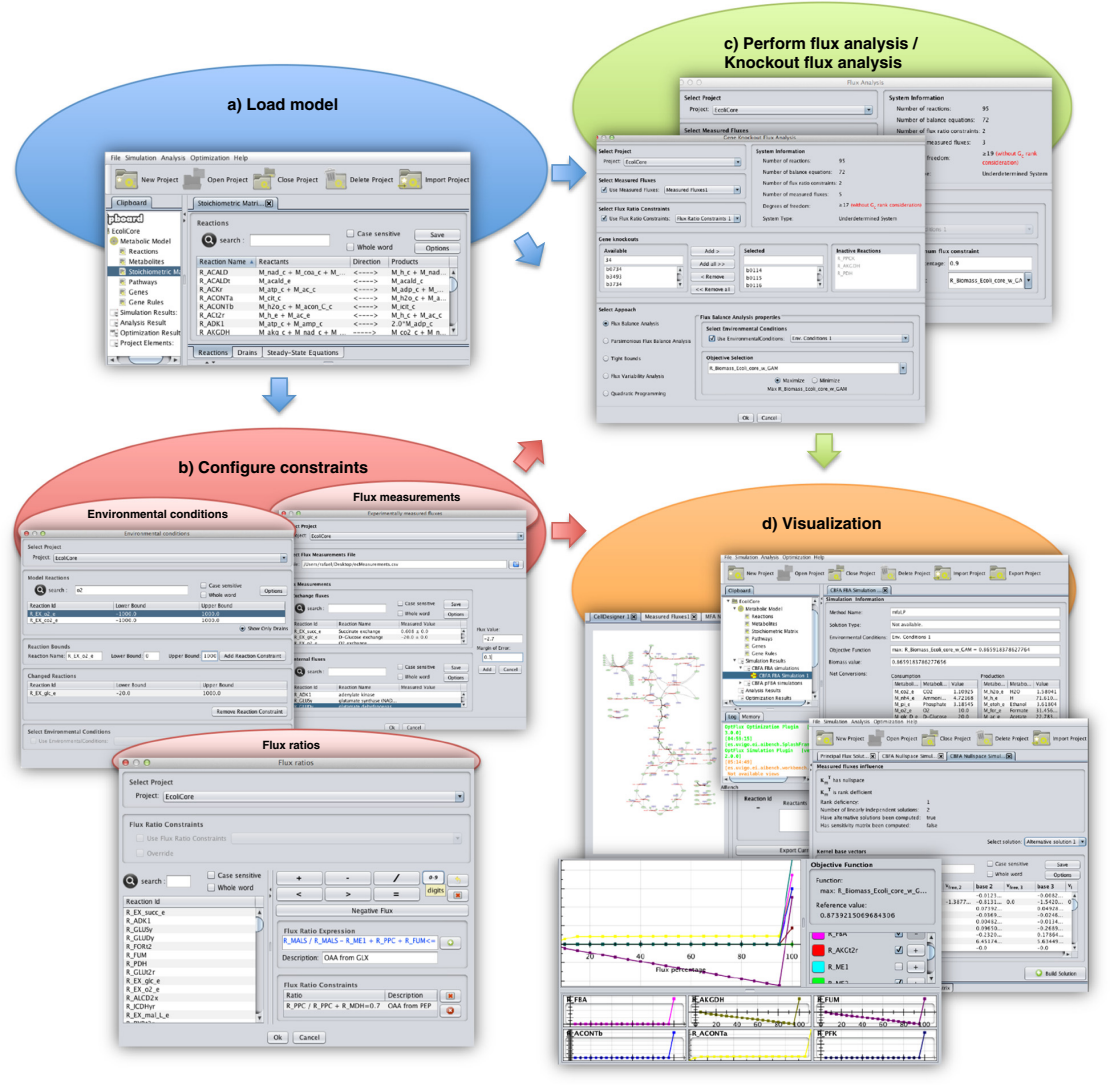
**Screenshots of CBFA: a) Load model – the first step is to load a metabolic model and a new project will be created and made available on the clipboard of the application.** Afterwards, it is possible to visualize their information through the available views; **b)** Configure constraints – different type of constraints can be configured through the use of graphical interfaces that enable to set the parameters to create the datatypes to be used on further operations; **c)** Perform flux analysis/knockout flux analysis – the user can select different constraints to perform several flux analysis methods using a metabolic model. It is also possible to configure a set of knockouts and perform flux analysis over the mutant with the same constraints; **d)** Visualization – the results of the operations create/update datatypes on the clipboard, and the output can always be checked through the views that are defined for the datatypes.

These concepts are used to build the application layer and to construct graphical user interfaces, which intermediate between the user and all the core methods implemented. In Figure 2, the architecture of the application is illustrated showing the main datatypes, views and operations, as well as the relationship with the core classes utilized in the implementation. A complementary view is given by Figure 3, which provides snapshots of some views and interfaces of the operations invoked when performing simulations and constraints configuration, showing a typical workflow when working with CBFA.

A metabolic model is loaded defining a working project, which is the root datatype in the hierarchical organization of the clipboard (left-hand side panel). The visualization of a selected object is presented on the right side. The structure and content of the visualization depends on the specific datatype, and on the object’s specific information. The hierarchical tree of the clipboard keeps all the results of the operations that are performed during the software utilization, namely datatypes for environmental conditions, flux measurements, flux ratios, and simulation results. Results are clustered in the clipboard according to the method used.

All the operations are performed on the scope of a certain project that keeps a single instance of a metabolic model. The other datatypes, such as flux measurements, can have multiple instances, under the same project, and the user can perform different tasks using alternative instances. Moreover, in the same running application, it is possible to have more than one project in the clipboard.

Operations are made available either on the top menu bar of the application or in a popup menu that is triggered when a right click event occurs upon a clipboard item. In the last case, only the operations that have the selected type as one of its input argument types are shown.

#### Case studies

To illustrate some of the main features of *CBFA*, two case studies were considered. First, the yeast *Saccharomyces cerevisiae* was investigated through the use of a simplified metabolic model for its growth [33], while in the second one, a genome-scale model of the microorganism *Escherichia coli* [34] was used to perform flux analysis tasks based on optimization methods. In the first case, the aim was to show how the implemented methods for (over)determined systems could be exploited. The model encompasses 45 reactions and 49 metabolites. The study was performed to analyse the phenotype response obtained by performing a deletion of the fumarate hydratase 1 reaction, through a knockout of the fum1 gene [35], under aerobic conditions with glucose as the carbon source.

Without adding constraints to the model, the system has 9 degrees of freedom. After setting the fum1 knockout, and adding the uptake and excretion rates given in [35], the configured system remained underdetermined. So, some flux ratios, such as the fraction of phosphoenolpyruvate originating from oxaloacetate and the fraction of oxaloacetate deriving from pyruvate [35], were added. In this way, the system was made determined. Thus, it was possible to calculate the intracellular fluxes (see the full results in the Additional file 4) through algebraic methods.

In a second case study, to demonstrate how optimization-based methods can be used to investigate flux distributions in GSMMs, an analysis was made using the *iJR904* model for *Escherichia coli* MG1655 [34]. This model contains 932 reactions, (including a biomass equation), 618 internal metabolites and 143 external metabolites (with respective drain fluxes) are present in the model. The original model has 332 degrees of freedom, and, therefore, the system was underdetermined and appropriate for the utilization of optimization-based methods, such as the ones based on linear programming approaches. In this case, the high number of degrees of freedom precludes the use of methods for determined or over-determined systems, as the amount of extra constraints to be added is very large.

To configure the experimental inputs to the analysis, the work from [36] was considered. Here, to study the influence of the inputs, the metabolic flux distribution was examined by incrementally adding constraints to the system. Analyses were made under aerobic conditions with glucose as the carbon source. The first step was to perform an FBA-based method. By analysing the flux distribution, the absolute values for the reactions R_ADK1 and R_ADK3 were considered to be too high. R_ADK1 was also occurring on the reverse direction, making a cycle with R_ADK3. After performing an FVA analysis, the two reactions had as lower and upper bounds the two extremes of their domains. Therefore, a pFBA-based method was performed, to minimize the total sum of the flux absolute values and remove this futile cycle. It was possible to confirm that the values for these two reactions changed in comparison to the FBA simulation, such that R_ADK1 changed its direction and R_ADK3 was set to zero. For the simulations performed in this first step, the only constraint that has been added was an environmental condition to set the glucose uptake rate to 11 mmol/gDW/hr. The objective was to maximize growth and the obtained value for the biomass flux was 0.97 hr^−1^.

In order to approximate the distribution according to [36], a second step was done, adding external flux measurements for O_2_, CO_2_ and acetate, which resulted in a decrease of the biomass flux to 0.81 hr^−1^. However, there were still some differences in the internal fluxes with respect to the published values. Therefore, some flux ratios were added and changes were detected in the internal flux distributions, such as the flux of phosphoenolpyruvate carboxylase and ATP:oxaloacetate carboxy-lyase. The detailed results (flux distributions) are given in Additional file 5, together with some notes on the conditions.

**Figure 4:**
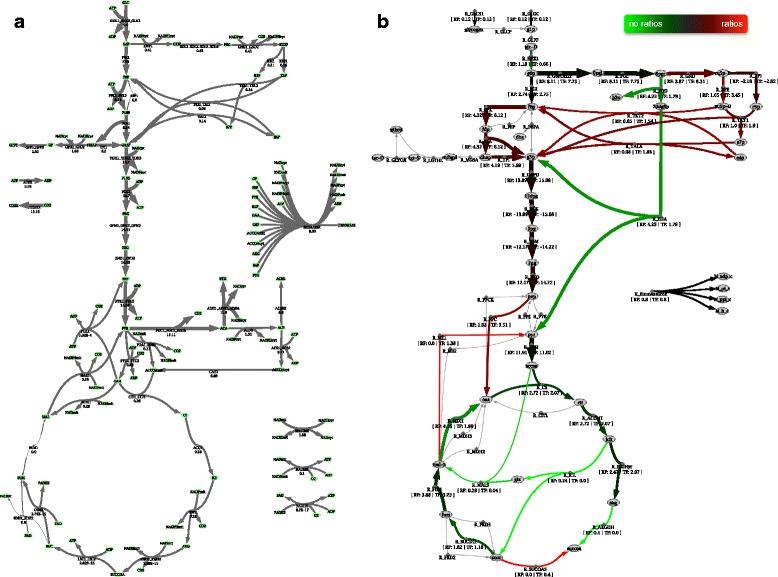
**Visualization of Flux Analysis results: a) The results of the flux calculation for Saccharomyces cerevisiae are illustrated.** The flux values were obtained through least squares, since the configured system was determined. The thickness of the arrows is proportional to the flux value of the corresponding reactions, after overlaying the fluxes over the network layout. Thin light grey arrows represent reactions with no flux value. **b)** The central carbon metabolism of Escherichia coli is shown with a comparison between pFBA-based simulations before and after adding metabolic flux ratio constraints. Here, grey arrows indicate reactions where there is no flux in both simulations, while red and green arrows represent reactions for which the simulations with and without flux ratios, respectively, returned flux values. The darker the arrows are, the nearer the fluxes in both simulations.

As an illustration of the previous results, in Figure 4, the distribution of the central carbon metabolism fluxes in both case studies is illustrated by overlaying fluxes over the network topology. This figure also serves as an example of the visualization capabilities of the tool.

## Conclusions

Although several software tools and applications have been developed and published to perform constrained-based flux analysis, currently none provides the full set of methods to perform flux analysis in a user-friendly way, while many are commercial or based on commercial systems such as Matlab. Thus, the software application described in this work is complementary to existing ones, since it provides a free, simple and quick way to perform flux analysis, with an extensive portfolio of methods, without all the complexity and specificity associated with experimental data processing. Therefore, it is possible to emulate the use of experimental data, by simply setting values (or intervals) for flux measurements, or to establish flux ratios that may reflect the over/ under expression of certain genes, or to translate the flux activity of a given pathway, for instance, obtained from ^13^C labelled substrate experiments.

## Availability and requirements

The software is made available, together with other resources, in the home page given below.

More details:

- Software name: *CBFA plugin for Optflux*
- Project home page: http://www.optflux.org
- Methods details and application tutorial: http://www.optflux.org/cbfa
- Operating system(s): Platform independent
- Programming languages: Java
- Other requirements: Java JRE 1.7.x (for Mac OS users the installation of JDK 1.7 is recommended), GLPK
- License: GNU-GPL, version 3

## Additional files

Additional file 1: Table S1. Feature comparison of several software tools for constraint-based methods, including a list of CBFA novel features under the framework of OptFlux. Additional file 2:
**Document with a full description and mathematical formulation of the implemented Flux Analysis methods.**
Additional file 3:
**CBFA tutorial.**
Additional file 4:
**Results for the simulations carried out in the first case study: reduced model of** ***S. cerevisiae*** **.**
Additional file 5:
**Results for the simulations carried out in the first case study: GSMM of** ***E. coli*** **.**

## Abbreviations

CBFA: Constraint-based Flux analysis
FBA: Flux balance analysis
FVA: Flux variability analysis
GLPK: GNU Linear Programming Kit
GSMMs: Genome-scale metabolic models
GUI: Graphical user interface
LP: Linear programming
ME: Metabolic engineering
MathML: Mathematical markup language
MFA: Metabolic Flux analysis
MOMA: Minimization of metabolic adjustment
MVC: Model view controller
pFBA: Parsimonious enzyme usage Flux balance analysis
QP: Quadratic programming
SBML: Systems biology markup language
