# Supplementary material for: CBFA: phenotype prediction integrating metabolic models with constraints derived from experimental data
Source: BMC Syst Biol. 2014 Dec 3;8:123. doi: 10.1186/s12918-014-0123-1 (PMC4263207; doi:10.1186/s12918-014-0123-1)
Supplement: Additional file 2: — Document with a full description and mathematical formulation of the implemented Flux Analysis methods. [file 12918_2014_123_MOESM2_ESM.pdf]

CBFA: phenotype prediction integrating genome-scale models with constraints derived from experimental data
Supplementary material: detailed methods

Rafael Carreira, Pedro Evangelhista, Paulo Maia,
Paulo Vilaça, Marcellinus Pont, Jean-François Tomb,
Isabel Rocha and Miguel Rocha

September 16, 2014

Contents

1 Introduction **6**

2 Basics of constraint-based modeling **7**

2.1 Metabolite balance 7

2.2 Other constraints 8

3 System determination **15**

4 Methods and formulations **19**

4.1 Optimization methods 19

4.1.1 Flux balance analysis 21

4.1.2 Parsimonious enzyme usage flux balance analysis . . . 22

4.1.3 Quadratic programming 24

4.1.4 Tight bounds calculation 26

4.1.5 Robustness Analysis 28

4.2 Algebraic methods 29

4.2.1 Algebraic calculations 30

4.2.2 Least squares 32

4.2.3 Weighted least squares 32

4.2.4 Null space 34

Methods symbols

Table 1: Symbols used in the equations and formulations of the methods.

Symbol	Description
X	vector with metabolite concentrations
v	vector with the metabolic fluxes
S	stoichiometric matrix of the model
v_i	i^{th} metabolic flux of the model
$\bar{v}_{m,i}$	mean of a set of measurements of the flux i
E_i	margin of error for the flux i
$\mathcal{M}$	set of indexes of the measured fluxes in the model
τ	metabolic flux ratio
$\mathcal{R}_{pos}$	set of indexes of the fluxes in the model that are set to be positive in the metabolic flux ratio constraints
$\mathcal{R}_{neg}$	set of indexes of the fluxes in the model that are set to be negative in the metabolic flux ratio constraints
$v_{objective}$	identifies the flux that has been selected to be maximized/ minimized
Lb_i	lower bound of flux i
Ub_i	upper bound of flux i
$\mathcal{K}$	set of indexes of the fluxes with knockouts
α	relaxing coefficient to be applied to the optimized objective
v_i^+	positive direction of the flux v_i, with respect to the stoichiometric matrix
v_i^-	negative direction of the flux v_i, with respect to the stoichiometric matrix
$v_{c,i}$	the ith calculated flux
v_c	row vector with the calculated fluxes
$\bar{v}_m$	row vector with the mean of the measured fluxes
H	an $(n \times n)$ symmetric matrix, being n is the number of measured fluxes, describing the coefficients of the quadratic terms
I	identity matrix

Continued on next page

Table 1 – continued from previous page

**Symbol**	**Description**
$t_{Lb,i}$	lower tight bound for flux i
$t_{Ub,i}$	upper tight bound for flux i
v_f	fixed flux in the objective solution
ρ	percentage of the fixed flux value
$v_{control,i}$	control flux i
ρ	percentage of the control flux value
$\mathcal{C}$	set of indexes of the fluxes for the robustness analysis
S_τ	extended stoichiometric matrix, with the last rows corresponding to the ratio equations in linear form
S_m	partitioning of S_τ, with the columns corresponding to the stoichiometric coefficients of the measured fluxes
S_c	partitioning of S_τ, with the columns corresponding to the stoichiometric coefficients of fluxes that are neither measured nor deleted through the application of knockouts
r_{S_c}	rank of the matrix S_c
${S_c}^+$	Moore-Penrose pseudoinverse of S_c
$\hat{v_m}$	weighted estimation of the measured fluxes
Ψ	variance-covariance matrix for the residuals of the measured fluxes
R	redundancy matrix that conveys the relations between all the measured reaction fluxes
R_r	reduced matrix of the redundancy matrix
$E(\sigma\sigma^T)$	measured flux variance matrix
σ_i^2	variance of the flux i
K	Kernel matrix that encompasses the base vectors that span the null space of S_τ
β	column vector with the free variables of the system
$ref(S_\tau)$	reduced echelon form of S_τ
U	upper triangular factor obtained from LU decomposition
L	lower triangular factor obtained from LU decomposition
$v_{f,i}$	free variable i

Continued on next page

Table 1 – continued from previous page

**Symbol**	**Description**
c_i	vector that corresponds to the ith special solution (the ith base vector of the null space of S_τ
K_m^T	matrix where its columns refer to the lines in K that correspond to measured fluxes
r_{K_m}	rank of K_m^T
U_s	matrix with the r_{K_m}-first rows of the upper factor of K_m^T
$U_{leading}$	invertible leading matrix
v_{ms}	vector with the r_{K_m}-first measured fluxes
γ_s	solution of forward substitution, by solving the lower triangular system in 31
y	solution of back substitution by solving the upper triangular system in 32
$v_{m,computed}$	vector with computed fluxes that have been measured

1 Introduction

The determination of metabolic fluxes is a fundamental resource to obtain insights regarding which enzymes participate in metabolic networks and also to simulate the cell's behavior under distinct types of genetic and environmental perturbations. Metabolic flux analysis (MFA) is widely used to calculate intra-cellular fluxes revealing the degree of pathway commitment in different metabolic processes during steady-state conditions, operating as a valuable tool in the detection of physiological alterations and to describe cell phenotypes.

Since intra-cellular metabolic fluxes cannot be directly measured, it is useful to have methodologies to estimate and calculate fluxes by applying mass balances around intra-cellular metabolites and from experimentally determined nutrient uptake and product secretion rates that can be readily obtained with standard methodologies. However, an accurate estimation of intracellular fluxes is not straightforward as metabolic networks have different alternative metabolic pathways leading to the production of the same metabolites [Bonarius et al., 1997].

^{13}C-isotopic tracing is a technique that can also be used to measure fluxes, where cells are grown on a ^{13}C-labeled carbon substrate, and the ^{13}C-labeling pattern in their proteinogenic amino acids can be determined through nuclear magnetic resonance or mass spectrometry. Through carbon labeling experiments and other knowledge on the relative pathway activity of an organism under certain conditions, it is also possible to obtain flux relationships in the form of ratios between sets of reaction rates [Sauer et al., 1999, McAnulty et al., 2012, Fischer et al., 2004].

This document describes methods regarding the application of MFA to metabolic models, making use of experimental data on the form of flux values for a set of the reactions in the model or, alternatively, flux ratios between sets of reaction fluxes. It is important to notice that this document does not pro-

vide any methods for the processing of fluxomics or metabolomics data in any specific format. Rather, it assumes the availability of general purpose data in the forms of flux values or flux ratios, regardless of their origin, and defines methods to work with those data.

The methods described here are implemented in the software tool *CBFA* that is described in the main paper. This document provides supplementary information detailing the mathematical formulation of the methods and some algorithmic details. The overall description of the software can be found in the main text, while the details of the software use are described in an additional document, a tutorial also available in the software's web site.

2 Basics of constraint-based modeling

It is important to notice that this software is integrated within a larger framework for constraint-based modeling approaches for Metabolic Engineering: OptFlux. As such, all methods described here work over a stoichiometric model that includes information on the set of reactions and metabolites occurring in the cell (or in a given system to be modeled). Other basic details in stoichiometric modeling and model structure can be found in the documentation of OptFlux.

2.1 Metabolite balance

From the stoichiometric metabolic model, a system of balance equations around the intra-cellular metabolites of the cell is built. The balance is made by setting the input flow to be equal to the output flow plus the accumulation of matter. Thus, the variation of the concentration in time for a given metabolite is given by the difference between the input flow in the metabolite and the output flow from it:

$$\frac{dX}{dt} = S.v \tag{1}$$

where X is a vector that contains the concentration of the m metabolites, v is an n dimensional vector with the metabolic fluxes and S is the m x n stoichiometric matrix of the model.

If the cell is assumed to be in a pseudo-steady state condition, it is assumed that there is no accumulation of matter and, therefore, the variation of the concentration of the metabolites will be zero along time and, as such [Heinrich and Schuster, 1996]:

$$S.v = 0 \tag{2}$$

The previous expression defines a set of m algebraic equations, where the fluxes are the n variables of the system. Usually, n is higher than m, specially when dealing with genome-scale metabolic models. This implies that trying to solve the underlying system usually leads to an under-determined system. An alternative is to define appropriate objective functions and use the constraints to restrict the solution space of the system (Figure 1).

2.2 Other constraints

To reduce the solution space of the system, thus approaching determined systems, it is possible to enhance the system with additional constraints originating from different sources (Figure 2). This information can be used to constrain directly the domain of the variables (fluxes), by defining bounds determined from reversibility (thermodynamics), experimental data, genetic and/ or environmental conditions. Alternatively, we can provide relationships between the reaction rates by setting up equalities (or inequalities)

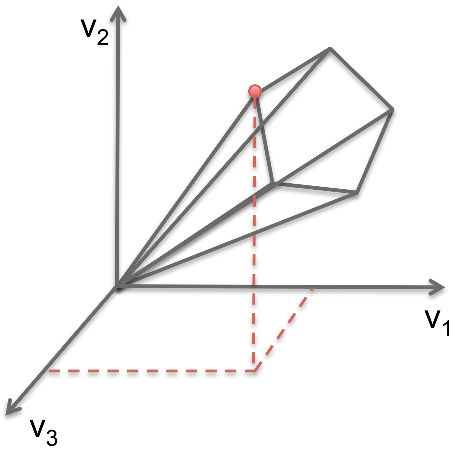

Figure 1: Solution space: allowable solution space of constraint-based modeling. The identified point represents the optimal solution.

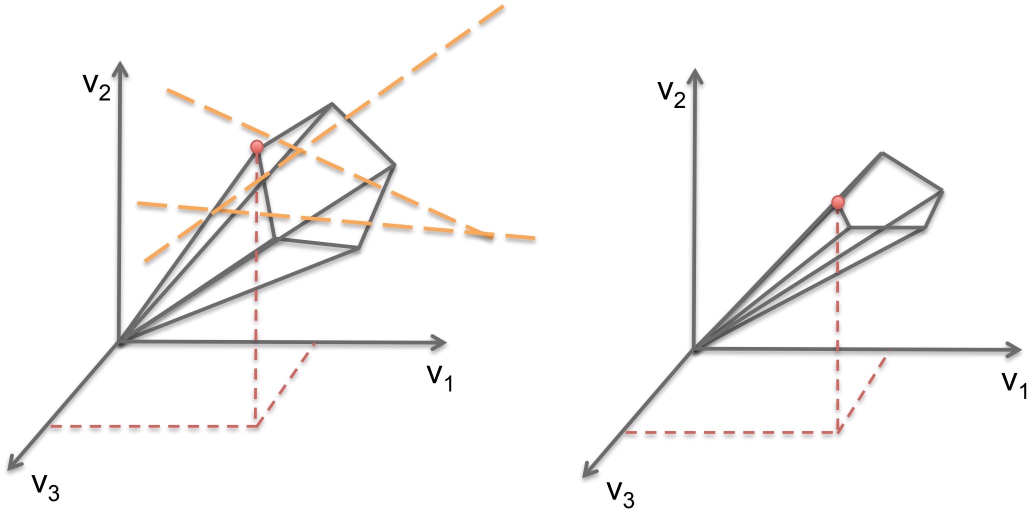

Figure 2: Reduced solution space: the allowable solution space is reduced with the constraints derived from the input data and the optimal solution may be shifted in the new flux cone.

involving reaction flux values, typically from fluxomics data.

From the stoichiometric model and thermodynamics knowledge about reversibility, upper and lower limits can be defined for the fluxes of each reaction. However if there is information about the environmental conditions

of a simulation (e.g. nutrients in the media), this can be used to reduce the domain for the fluxes (Figure 3.B).

More precise bounds can be found from experimental data (e.g. fluxomics) providing measurements for the rates of formation and consumption of some compounds involved in the network, such as extra-cellular substrates (e.g. glucose) and O_2, as well as products (such as CO_2, acetate and lactate). This kind of data can be used to set the fluxes to have a certain value, or to respect a range for specific phenotype simulations (Figure 3.C). In such case, if a set of replicates for the measurements are given for a specific flux, these values can be used to calculate the mean and the margin of error of the flux value and, consequently, reduce the domain of the flux as follows:

$$-E_i + \bar{v}_{m,i} \leq v_i \leq \bar{v}_{m,i} + E_i \quad \forall i \in \mathcal{M} \qquad (3)$$

where $\bar{v}_{m,i}$ is the mean of a set of measurements of the flux i, E_i is the margin of error for the flux i and $\mathcal{M}$ is the set of indexes of the measured fluxes in the model.

When simulating the phenotype of a mutant strain, it is common to define the knockout of a set of genes (or reactions). In such case, the reactions encoded by the genes for which the knockouts were defined (or directly the selected reactions) are constrained to have a flux equal to zero (Figure 3.D). Please check the OptFlux documentation for further details on these kind of simulations and their use for strain optimization purposes.

Another type of constraints that can be incorporated in the system is to define a relationship between the fluxes in the model. These relationships are constructed as flux ratio equations, where it is possible to model the amount of a certain flux (or a set of fluxes) with respect to other fluxes. These metabolic flux ratios constraints are in the form:

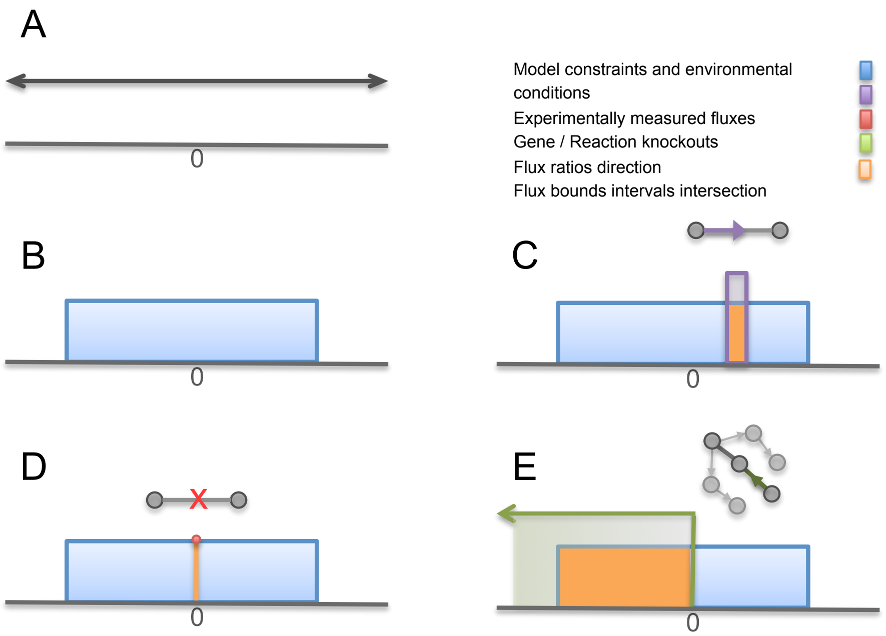

Figure 3: Flux bounds obtained from different type constraints. **A**: the flux has no constraint either on the upper or lower bound. **B**: the domain of the flux is defined by the model or environmental conditions. **C**: the bounds of the flux are set from experimentally measurements of the flux. **D**: the flux is set to have a zero value since it corresponds to a knockout (gene or reaction). **E**: the bounds are configured so that the flux respects the direction (relating to the stoichiometric matrix) in which it appears in the flux ratio constraint

$$\frac{\sum_{i=1}^{n} \kappa_i v_i}{\sum_{j=1}^{n} \kappa_j v_j} = \tau \tag{4}$$

where κ_i, $\kappa_j \in \mathbb{R}$ are constants, v_i and v_j are fluxes, and $\tau \in \mathbb{R}\backslash\{0\}$ is the ratio between the numerator and denominator fluxes. In this case, the operator that relates the fluxes is the equality operator, but it is possible to define inequalities, where the ratio represents a upper or lower bound.

To add this type of constraints to the system, the ratio equations are converted to its linear form:

$$\sum_{i=1}^{n} \kappa_i v_i - \tau \sum_{j=1}^{n} \kappa_j v_j = 0 \tag{5}$$

Some problems arise from this kind of equations. For instance, in a simple case, where a ratio between two fluxes (e.g. $v_1/v_2 = 0.5$) is set, it is expressed that v_1 is constrained to have a value that is half the value of v_2. However, if v_1 is the flux of a reversible reaction, and this reaction is occurring in the negative direction with respect to the stoichiometric matrix, mathematically this ratio will force the flux v_2 to be also negative. Because the ratio is a positive number, both the numerator and denominator of the equation must have the same signal. So, this kind of expressions has an influence in the signal of the fluxes of the solution space. Moreover, if we are dealing with inequality constraints, in the conversion of the expressions to its linear form, if the signal of the flux is not known, it is not possible to determine if the inequality operator will need to be changed or not.

Therefore, either the expression is changed to take into account the absolute value of the fluxes or the direction of the flux has to be set in the expression. If we assume that the direction of the flux is set, this kind of expression will add two types of constraints to the system. Firstly, the expression that constrains the fluxes absolute values is added to the system, and afterwards

the bounds of the fluxes are updated to respect the direction of the flux in the expression (Figure 3.D). These bounds are configured in the form:

$$v_i \geq 0 \quad \forall i \in \mathcal{R}_{pos} \tag{6}$$

where the flux i is in the positive direction and $\mathcal{R}_{pos}$ is the set of indexes of the fluxes in the model that are set to be positive in the metabolic flux ratio constraints.

$$v_i \leq 0 \quad \forall i \in \mathcal{R}_{neg} \tag{7}$$

where the flux i is in the negative direction and $\mathcal{R}_{neg}$ is the set of indexes of the fluxes in the model that are set to be negative in the metabolic flux ratio constraints.

If there is a non empty intersection between the two previous sets, it is necessary to define how to deal with this case in terms of flux bounds. A priority list is defined such that its order defines which constraint will be assumed, if it is not possible to take into account both constraints, set as follows:

1. Gene/ reaction knockouts;
2. Measured fluxes;
3. Ratio fluxes direction;
4. Environmental conditions;
5. Model constraints.

If an overlap of a constraint into another occurs, and it is possible to consider both of them, the domain of the flux is updated with this consideration (orange region in Figure 3.E where the lower bound is defined by the stoichiometry, thermodynamics or environmental conditions and the upper bound is set to zero meaning that the flux is negative in the ratio expression it belongs to).

3 System determination

Since the number of reactions is typically greater than the number of the metabolites in the model, there is a certain degree of freedom in the set of the algebraic equations related to the metabolite balance. Usually, these models are, therefore, under-determined even with the additional constraints obtained as it was introduced in the previous chapter. However, in some cases, this might not be the case, i.e. there may be enough constraints to allow solving the system or even to over-constrain it. In order to determine the type of system, it is necessary to take into account the several inputs and related constraints. Firstly, from the stoichiometric model, the number of reactions and the number of mass balance equations (balanced metabolites) are retrieved (Figure 4).

Since the balance equations have the reaction fluxes as its unknown variables, depending on the number of the equations in the system, it might not be possible to algebraically solve the system if the number of linearly independent equations is less than the number of variables. Indeed, this difference between the number of reactions and the number of mass balance equations defines the number of degrees of freedom in the model:

$$\#\begin{array}{c}\text{model degrees}\\ \text{of freedom}\end{array} = \#\,reactions - \#\begin{array}{c}\text{linearly independent}\\ \text{balance equations}\end{array} \tag{8}$$

However, it is possible to reduce this number through the consideration of the other constraints mentioned in the previous section. If experimentally measured fluxes are given with exact values, i.e., there is no margin of error from the measurements replicates, each flux can be set to the measured value, thus possibly reducing the number of degrees of freedom, as the number of unknowns decreases.

If a margin of error exists for a flux, the measurements are still used but they do not set a fixed value for the flux, but instead a range of possible values (Eq.3). In this case, the flux still has to be calculated, and the number of degrees of freedom is not reduced (although the new constraints will limit the feasible space).

It is also possible to decrease the number of degrees of freedom by adding gene/ reaction knockouts. In these cases, the flux values for the affected reactions are set to be equal to zero, and consequently decreasing the number of unknown variables of the system by a value equal to the number of affected reactions.

When a set of metabolic flux ratios is given, the equality ratios define new linear constraints to add to the ones coming from the model and, therefore, they are likely to reduce the degrees of freedom of the model (by a number equal to the number of linearly independent expressions they add to the system).

As it happens with the experimentally measured fluxes, the inequality equations are not used to reduce the degrees of freedom, though they are used to constrain the solution space of the system.

As it will be explained on a posterior section of this document (section 4.2), to solve the system algebraically, the stoichiometric matrix of the model is extended with the flux ratios and partitioned, obtaining a smaller matrix, S_c corresponding to the unknown fluxes, i.e. fluxes that are neither measured nor deleted through the application of knockouts in the case of mutant strains. For now, it is important to mention that this matrix is the one that is used to calculate the real degrees of freedom of the configured system from the input data. Therefore, the degrees of freedom of the system can be obtained as:

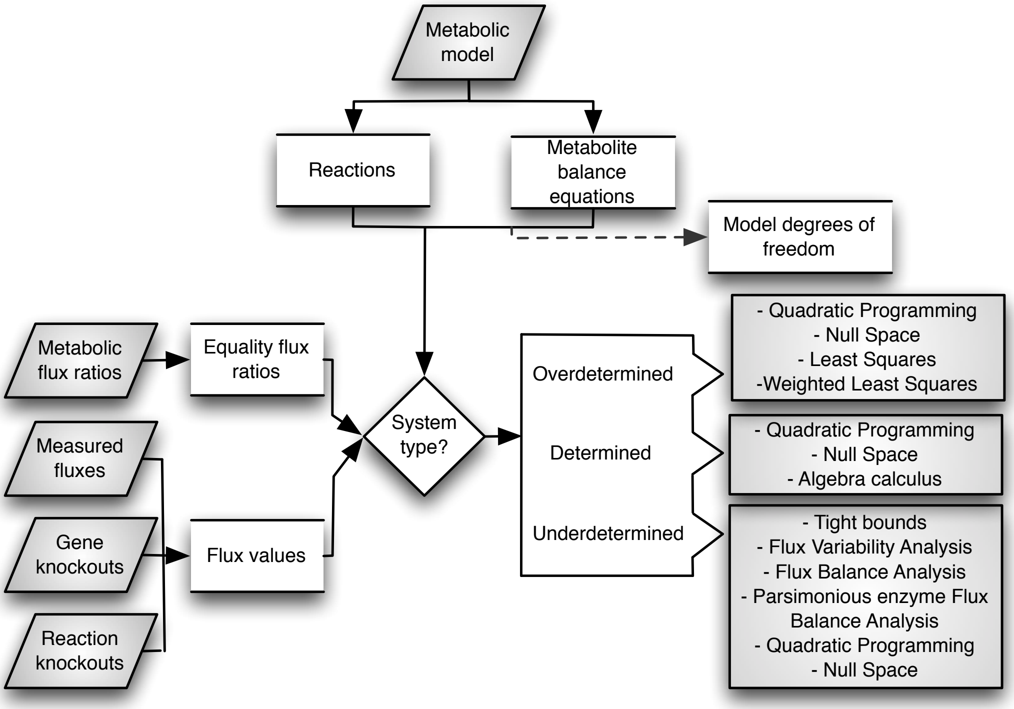

Figure 4: Model degrees of freedom: the type of the system is inferred from the degrees of freedom of the model and the constraints given to the system. The parallelogram shapes represent the inputs of the system and the light grey rectangles are the methods that can be applied to each type of system.

$$\# \begin{array}{c}\text{system degrees}\\ \text{of freedom}\end{array} \;=\; \#\, unknown\, reactions \;-\; r_{S_c} \qquad (9)$$

where r_{S_c} stands for the rank of the matrix S_c.

Although the existence of flux measurements and knockouts may reduce the number of unknowns of the system, because they depend on the r_{S_c} matrix, there is not a proportional decrease, i.e. the decrease in the degrees of freedom may be less than the number of equality constraints introduced. This happens since the elimination of the columns (corresponding either to measured fluxes or to deleted reactions) from the stoichiometric matrix may result on the appearance of new linearly dependent rows, thus limiting the reduction in the overall degrees of freedom.

When considering all possible constraints for a given simulation, the number of degrees of freedom is calculated taking into account the original model, the environmental constraints, the knockouts defined (if any) and all constraints coming from measured data. If the overall number of degrees of freedom is greater than zero, the system is said to be underdetermined. This is the most common case, typically obtained when dealing with large-scale models (e.g. genome-scale). On the other hand, in the particular case where the number of degrees of freedom equals zero, the information in the system is enough to calculate all the unknowns, and the system is called determined. In the more general case in which the additional constraints reduce the number of degrees of freedom by a value larger than the original one (i.e. the overall calculation of the degrees of freedom would be negative), the system is said to overdetermined and the additional data can be used to better fit the unknowns. The overall process of system determination is provided in Figure 4.

4 Methods and formulations

Depending on the type of the designed system, different approaches can be used to determine the unknown fluxes. When the system is under-determined, optimization methods to search for an optimum solution in the constrained solution space are used. In the cases where the system is determined, algebraic methods can be directly used to solve the system and calculate the unknowns (flux values). If the system is over-determined, algebraic methods are used to fit the variables of the system to the data. Although there is this separation between the methods that are available depending on the type of the system, the null space method and quadratic programming optimization are formulated to be applied for all types of systems.

From the different inputs of the system, it is possible to organize the data in classes of data types (Figure 5). The stoichiometric matrix is built from the metabolic model where every row of the matrix is related to the mass balance of an intra-cellular metabolite. The flux bounds include the set of lower and upper limits of reaction rates given by the model, the flux limits related to environmental conditions, and also the intervals defined by the experimentally measured fluxes according to the mean and margin of error of the given replicates. The set of flux values comprehends the fluxes that must have zero values originating from the knockouts and also the fixed measured fluxes, i.e., when the margin of error is not considered. Finally, the list of metabolic flux ratios is split into a set for the equality equations and another set for the inequality equations. Figure 5 provides a schematic representation of the data types and available methods.

4.1 Optimization methods

This section describes the formulation of the optimization methods, which estimate the flux values through the definition of an objective function and

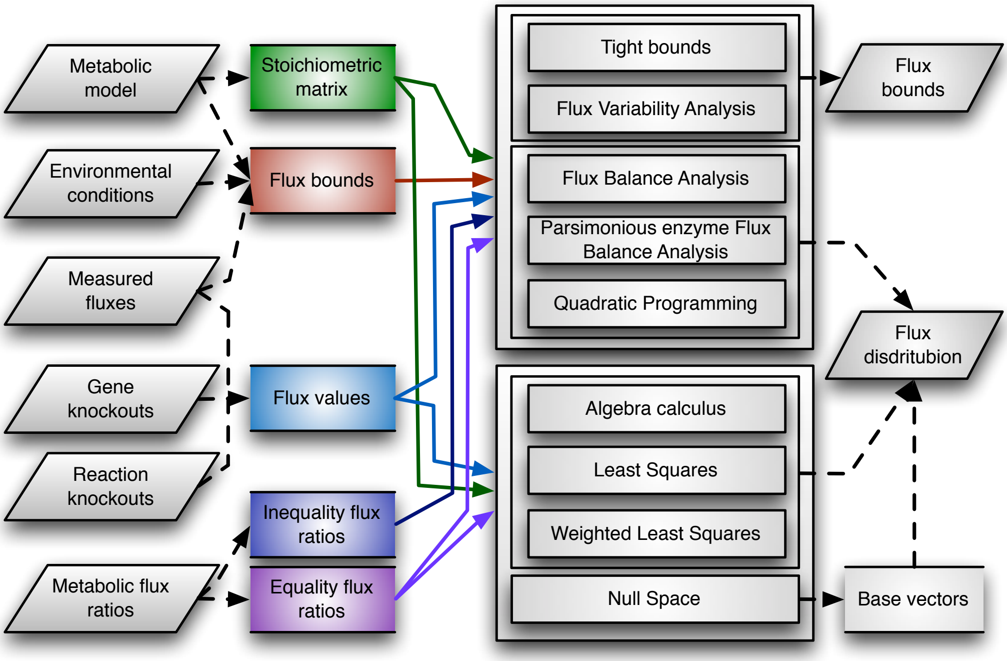

Figure 5: Methods inputs and outputs: the inputs of the system are partitioned into different classes of data types that are used by the MFA methods. The parallelogram shapes on the left and on the right represent the inputs and outputs, respectively, of the system. The colored rectangles denote the classes of data types.

using all the inputs of the system to constrain the space of admissible solutions.

4.1.1 Flux balance analysis

Flux balance analysis (FBA) [Papoutsakis, 1984] is a mathematical modeling approach widely used to quantitatively simulate the values of metabolic fluxes of a certain organism under a set of physiochemical conditions. It is formulated as a linear programming (LP) problem that maximizes (or minimizes) a configured objective function, such as the biomass flux (an artificial flux representing the growth rate). This approach searches for the maximum (or minimum) value of the objective function that respects the constraints that are configured from the input data:

$$
\begin{aligned}
\text{maximize/minimize} \quad & v_{objective} \\
\text{subject to:} \quad & S.v = 0 \\
& \textstyle\sum_{i=1}^{n} \kappa_i v_i - \tau \sum_{j=1}^{n} \kappa_j v_j = 0 \\
& Lb_i \leq v_i \leq Ub_i && \forall i \notin \mathcal{M} \cup \mathcal{K} \\
& -E_i + \bar{v}_{m,i} \leq v_i \leq \bar{v}_{m,i} + E_i && \forall i \in \mathcal{M} \\
& v_i = 0 && \forall i \in \mathcal{K} \\
& v_i \geq 0 && \forall i \in \mathcal{R}_{pos} \\
& v_i \leq 0 && \forall i \in \mathcal{R}_{neg}
\end{aligned}
$$

where S is the m x n stoichiometric matrix of the model, v is an n dimensional vector with the metabolic fluxes where v_i is the ith flux of the model, Lb_i and Ub_i are the upper and lower bounds, respectively, of the fluxes, $\bar{v}_{m,i}$ is the mean of a set of measurements of the flux i (or a fixed value if there is only one measurement of the flux), E_i is the margin of error for the flux i (or

zero if there is only one measurement of the flux), $\mathcal{M}$ is the set of indexes of the measured fluxes in the model, $\mathcal{K}$ is the set of indexes of the fluxes with knockouts and $\mathcal{R}_{pos}$ and $\mathcal{R}_{neg}$ are the set of indexes of the fluxes that are set be positive and negative, respectively, in the metabolic flux ratio constraints.

The lower and upper bounds of the variables are obtained as previously described and added to the problem, where the equality ones are configured to have the same value for the lower and upper bounds. With respect to the equations that translate the metabolic flux ratios, here both the equality and inequality constraints are used, by adding the linear constraints to the problem.

4.1.2 Parsimonious enzyme usage flux balance analysis

In the parsimonious enzyme usage flux balance analysis (pFBA) there is the assumption that, under growth pressure, the strains that process the growth rate most rapidly and efficiently, with the minimum enzyme usage, are selected. It is possible to approximate this theory through the optimization of the growth rate using FBA, and afterwards minimizing the metabolic fluxes related to all reactions in the model [Lewis et al., 2010]. This assumption has been the motivation for the formulation of an MFA method that searches for the minimal sum of all fluxes in the metabolic network that contributes for an outlined objective function, such as the maximization of the biomass, or another target compound.

This is accomplished in two sequential steps: first an optimization of the objective function is performed through the application of the FBA-based MFA method, outlined in the previous section, and in the second step the obtained objective value is fixed (as an additional constraint) and a new linear programming problem is configured to minimize the sum of all fluxes, keeping all other constraints unchanged. The overall formulation is therefore the following:

$$
\begin{array}{llr}
\text{minimize} & \sum_{i=1}^{n} |v_i| & \\
\text{subject to:} & v_{objective} = \alpha \ \max/\min \ v_{objective} & \alpha \in \mathbb{R}^{+} \\
& S.v = 0 & \\
& \sum_{i=1}^{n} \kappa_i v_i - \tau \sum_{j=1}^{n} \kappa_j v_j = 0 & \\
& Lb_i \leq v_i \leq Ub_i & \forall i \notin \mathcal{M} \cup \mathcal{K} \\
& -E_i + \bar{v}_{m,i} \leq v_i \leq \bar{v}_{m,i} + E_i & \forall i \in \mathcal{M} \\
& v_i = 0 & \forall i \in \mathcal{K} \\
& v_i \geq 0 & \forall i \in \mathcal{R}_{pos} \\
& v_i \leq 0 & \forall i \in \mathcal{R}_{neg}
\end{array}
$$

where $v_{objective}$ is the optimized objective flux, α is a relaxing coefficient to be applied to the optimized objective and all other symbols take the meaning explained in the previous section.

Because the formulated objective function is non-linear, due to the absolute values of the fluxes, it has to be rearranged in order to perform linear programming to solve the problem. Therefore, each flux variable is decomposed in two different variables, and the following set of constraints are added to the problem:

$$
\begin{aligned}
& v_i + v_i^{-} - v_i^{+} \\
& v_i^{+} \geq 0 \\
& v_i^{-} \geq 0
\end{aligned} \tag{10}
$$

where v_i^{+} and v_i^{-} correspond to the positive and negative direction of the flux v_i, respectively. To eliminate the absolute values of the objective function,

each of it is replaced as follows

$$|v_i| = v_i^+ + v_i^- \tag{11}$$

Since the formulation is a minimization problem, the simplex algorithm will ensure that at least one of the directions of each flux will be equal to zero, and the values of the two directions of the flux v_i are obtained as:

$$\begin{cases} v_i > 0, v_i^+ = v_i \ \ and \ \ v_i^- = 0 \\ v_i < 0, v_i^+ = 0 \ \ and \ \ v_i^- = v_i \\ v_i = 0, v_i^+ = 0 \ \ and \ \ v_i^- = 0 \end{cases} \tag{12}$$

4.1.3 Quadratic programming

When a set of flux measurements exists, it is possible to formulate a problem that estimates the network fluxes in order to minimize the difference between the measured values and the calculated ones, keeping the other constraints of the system active.

This approach is similar to the variant of FBA called minimization of metabolic adjustment (MOMA) [Segre et al., 2002], which has the assumption that mutant strains show flux distributions that are as close as possible to the wild-type optimum. Here, instead the approximation to a reference flux distribution, the fluxes that have been measured are selected to be approximated.

Thus, the objective function of this optimization problem can be defined as the minimization of the sum of the squares of the differences between the measured and the calculated fluxes, as given next:

$$\text{minimize} \sum_{i=1}^{n} (v_{c,i} - \bar{v}_{m,i})^2 \tag{13}$$

where n is the number of measured fluxes, $v_{c,i}$ is the ith calculated flux and $\bar{v}_{m,i}$ is the mean of a set of measurements of the flux i or a fixed value if there is only one measurement of the flux.

Since all constraints are linear and the objective function is quadratic, this configures a problem of quadratic programming (QP) optimization, where the objective function can be converted to the form:

$$\text{minimize} \frac{1}{2} v_c^T H v_c - 2\bar{v}_m^T v_c + \sum_{i=1}^{n} \bar{v}_{m,i}^2 \tag{14}$$

where v_c is an n-dimensional row vector with the calculated fluxes, $\bar{v}_m$ is an n-dimensional row vector with the mean of the measured fluxes (or fixed values if there is only one measurement of the fluxes), H is an $(n \times n)$ symmetric matrix, being n is the number of measured fluxes. The scalar multiplication $-2\bar{v}_m$ represents the coefficients of the linear terms in the objective function and H describes the coefficients of the quadratic terms. In this optimization problem, the H matrix is the identity matrix multiplied by 2:

$$H = 2.I \tag{15}$$

where I is an $(n \times n)$ identity matrix. Therefore, the objective function can be simplified by removing the scalar 1/2 and the H matrix from the expression, and the equation 14 can be rewritten as:

$$\text{minimize}\ v_c^T v_c - 2\bar{v}_m^T v_c + \sum_{i=1}^{n} \bar{v}_{m,i}^2 \tag{16}$$

Regarding the constraints, its formulation is similar to the previous methods, with a slight difference since the measured fluxes are not added to the constraints rows (they are used instead in the objective function in this case). The overall formulation is the following:

$$
\begin{aligned}
\text{minimize} \quad & v_c^T v_c - 2v_m^T v + \sum_{i=1}^{n} \bar{v}_{m,i}^2 \\
\text{subject to:} \quad & S.v = 0 \\
& \textstyle\sum_{i=1}^{n} \kappa_i v_i - \tau \sum_{j=1}^{n} \kappa_j v_j = 0 \\
& Lb_i \leq v_i \leq Ub_i && \forall i \notin \mathcal{M} \cup \mathcal{K} \\
& v_i = 0 && \forall i \in \mathcal{K} \\
& v_i \geq 0 && \forall i \in \mathcal{R}_{pos} \\
& v_i \leq 0 && \forall i \in \mathcal{R}_{neg}
\end{aligned}
$$

where all the relevant symbols have been presented above. In this formulation there is a constant term in the objective function related to the sum of the squares of the flux measurements, that can be dropped from the formulation when the problem is set up to be solved [1].

4.1.4 Tight bounds calculation

The tight bounds method uses a different approach from the previous ones, since it does not return a flux distribution that better suits an objective function, but instead it computes the lower and upper limits for every non free flux of the system and thus characterizes the boundaries of the system under certain conditions.

[1] Quadratic programming problems can be solved with some specific solvers. Please refer to the tutorial document (available on `http://www.optflux.org/cbfa`) to check the solvers that are supported in *CBFA*.

The FBA-based MFA method allows configuring optimization problems optimizing specific fluxes. This feature is used here, for every flux of a reaction in the model that has neither been experimentally measured nor has an associated knockout, to calculate both its minimum and maximum values under the same constraints. The result of each minimization and maximization optimization process is taken as the lower and upper bound, respectively, of this flux.

The formulation for all minimization and maximization problems uses the same constraints as defined in the FBA and pFBA-based method, given by:

$$
\begin{aligned}
t_{Lb,i} = \text{minimize} \quad & v_i & \forall i \notin \mathcal{M} \cup \mathcal{K} \\
t_{Ub,i} = \text{maximize} \quad & v_i & \forall i \notin \mathcal{M} \cup \mathcal{K} \\
\text{subject to:} \quad & S.v = 0 & \\
& \textstyle\sum_{i=1}^{n} \kappa_i v_i - \tau \sum_{j=1}^{n} \kappa_j v_j = 0 & \\
& Lb_i \leq v_i \leq Ub_i & \forall i \notin \mathcal{M} \cup \mathcal{K} \\
& -E_i + \bar{v}_{m,i} \leq v_i \leq \bar{v}_{m,i} + E_i & \forall i \in \mathcal{M} \\
& v_i = 0 & \forall i \in \mathcal{K} \\
& v_i \geq 0 & \forall i \in \mathcal{R}_{pos} \\
& v_i \leq 0 & \forall i \in \mathcal{R}_{neg}
\end{aligned}
$$

where $t_{Lb,i}$ and $t_{Ub,i}$ are the tight bounds of the flux i and the other symbols take the meaning defined in the previous cases.

Flux variability analysis

The approaches based on LP problems can have multiple solutions that satisfy all the constraints and, nevertheless, have the same optimal value for the objective function. To achieve the characterization of alternative solutions that satisfy all the constraints and allow for the same optimal objective value,

flux variability analysis (FVA) can be applied to determine the admissible ranges of flux values under optimality [Mahadevan et al., 2003].

This method is a slight variation of the tight bounds method. First, it is performed a simulation related to the objective function through the application of the FBA method. From this simulation, the solution with the flux distribution is kept and a flux is selected to be fixed in the calculation of the tight bounds. It is also possible to configure the value of the fixed flux to be a percentage of its value (typically varying from 0 to 100%) in the wild-type strain. The only difference in respect to the tight bounds formulation, is the addition of the following constraint:

$$v_f = \rho \max/\min v_f \quad , \quad \rho \in \mathbb{R}^+ \tag{17}$$

where f is index of the fixed flux in the objective solution and ρ is the percentage of the fixed flux value.

4.1.5 Robustness Analysis

Robustness analysis is a method that enables to investigate how an objective function changes in response to variations in the optimal fluxes of specific reactions of the metabolic model [Edwards and Palsson, 2000, Palsson, 2006]
.

A first simulation is performed to determine the optimal flux distribution for a defined optimization problem (e.g. maximization of the growth rate). The result of this simulation is kept as the control distribution. For each reaction selected to perform the robustness analysis, its flux is varied from 0 to 100% of its value in the control distribution, and the optimization problem is solved with this additional constraint. The result is a set, for each selected flux, containing the resulting objective values for the control flux variation.

The formulation to maximize/minimize an objective function for each control flux variation is given as follows:

$$
\begin{aligned}
\text{maximize/minimize} \quad & v_{objective} & \\
& v_i = \rho \; v_{control,i} & 0 \leq \rho \leq 100, i \in \mathcal{C} \\
& S.v = 0 & \\
& \textstyle\sum_{i=1}^{n} \kappa_i v_i - \tau \sum_{j=1}^{n} \kappa_j v_j = 0 & \\
& Lb_i \leq v_i \leq Ub_i & \forall i \notin \mathcal{M} \cup \mathcal{K} \\
& -E_i + \bar{v}_{m,i} \leq v_i \leq \bar{v}_{m,i} + E_i & \forall i \in \mathcal{M} \\
& v_i = 0 & \forall i \in \mathcal{K} \\
& v_i \geq 0 & \forall i \in \mathcal{R}_{pos} \\
& v_i \leq 0 & \forall i \in \mathcal{R}_{neg}
\end{aligned}
$$

where $\mathcal{C}$ is the set of indexes of the fluxes that are selected for the robustness analysis, $v_{control,i}$ is the control flux for which the problem is formulated and ρ is the percentage to vary the control flux.

4.2 Algebraic methods

In the methods described in this section, the metabolic flux ratio equations are added to the ones coming from the stoichiometric matrix and mass balance equations. However, bound constraints are not incorporated in the rows of the stoichiometric matrix, and therefore only equality equations are considered in the following methods. Therefore, flux ratios given by inequalities are ignored in these methods.

Equation 5 is used to convert the ratios to its linear form and these are added as new rows of the stoichimetric matrix, where the coefficients of the

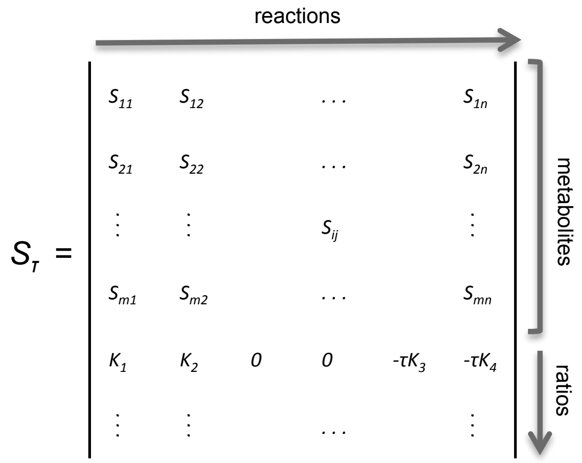

Figure 6: Extended stoichiometric matrix: The stoichiometric matrix is extended with the linear ratio equations. The columns represent the reactions of the model, the first rows correspond to metabolite balance equations of the system and the last rows represents the coefficients of the equality metabolic flux ratios.

fluxes in the ratio equations, and its product to the negative of the ratio in the equation, are added to the corresponding reaction column (Fig 6). The balance equation in Eq. (2) is redefined accordingly:

$$S_{\tau}.v = 0 \tag{18}$$

where S_{τ} is an $(m+r)$ x n extended stoichiometric matrix with the last r rows corresponding to the ratio equations linear form, and v is an n dimensional vector with the metabolic reaction rates.

4.2.1 Algebraic calculations

If the extended matrix has no linearly dependent rows ant the number of flux measurements equals to the difference between the number of reactions and the number of balance equations, the solution of the system is unique

and it can be obtained by solving the linear system of equation 18. This is accomplished through the separation of the measured fluxes from the non-measured ones in the vector v. For this purpose, the coefficients in the extended stoichiometric matrix S_τ have to be partitioned as well. Therefore, equation 18 can be reformulated as:

$$S_m v_m + S_c v_c = 0 \tag{19}$$

where S_m and S_c represents the partitioning of the matrix S_τ with the columns corresponding to the stoichiometric coefficients of the fluxes that have been measured and the columns of the non-measured fluxes, respectively, and v_m and v_c are the vectors of the measured and unknown fluxes, respectively.

If S_c is an n-by-n square matrix, n equals to the number of metabolites plus the number of equality ratio constraints, and also equals to the number of unknown fluxes. In such case, if S_c is not a singular matrix, the matrix can be inverted and the previous equation can be rewritten such that the vector of the unknown reactions rate can be obtained from the stoichiometric matrix rearrangement and the experimentally measured fluxes:

$$v_c = -{S_c}^{-1} S_m v_m \tag{20}$$

However, if S_c is an m-by-n non-square matrix, it cannot be inverted and the system can not be solved from the above equation. Therefore, the solution of the equation 20 is rather found by the application of the Moore-Penrose pseudoinverse of S_c [Penrose, 1955]:

$$v_c = -{S_c}^{+} S_m v_m \tag{21}$$

where ${S_c}^+$ is the Moore-Penrose pseudoinverse of S_c.

Because the number of equations in S_c (m) will be less than the number of the unknown fluxes (n), the Moore-Penrose pseudoinverse of S_c is obtained through the calculation of the right inverse of S_c. Thus, equation (21) can be rewritten in the form:

$$v_c = -\left(S_c({S_c}^T S_c)^{-1}\right) S_m v_m \tag{22}$$

4.2.2 Least squares

If the system is overdetermined, the additional information can be used to test the consistency of the overall balances and the accuracy of the flux measurements [Stephanopoulos et al., 1998]. In this scenario, equation 21 can also be used. However, since in this case the number of rows in the S_c matrix is greater than the number of its columns, the Moore-Penrose pseudoinverse of S_c is obtained through the calculation of the left inverse of S_c, and equation 21 can be rephrased as:

$$v_c = -\left((S_c {S_c}^T)^{-1} S_c\right) S_m v_m \tag{23}$$

This equation represents the least square estimation of the unknown reactions fluxes.

4.2.3 Weighted least squares

Since experimentally measured data usually have associated errors, the previous method is not as accurate as it could be, because it does not assume that there is a variance for the residuals of the measured fluxes. If informa-

tion about the variance on the flux measurements exists, this variance can be used to weight the least squares fitting. Consequently, the best weighted estimation of the measured fluxes on an over-determined system can be obtained by:

$$\hat{v_m} = \left(I - E(\sigma\sigma^T){R_r}^T\Psi^{-1}R_r\right)v_m \tag{24}$$

where I is the identity matrix, Ψ is the m x m variance-covariance matrix for the residuals of the measured fluxes and R_r is a reduced matrix of the redundancy matrix R. [Wang and Stephanopoulos, 1983] show that the estimates for the measured reaction rates results in a smaller standard deviation, compared to the the direct use of the measurements, making the estimates likely to be more reliable to calculate the non measured rates. The variance-covariance matrix can be obtained as:

$$\Psi = R_r E(\sigma\sigma^T)R_r^T \tag{25}$$

where $E(\sigma\sigma^T)$ is an m x m diagonal matrix, called measured flux variance matrix, such that the element in the i-th row and in the i-th column correspond to the variance of the flux i [Madron et al., 1977]:

$$E(\sigma\sigma^T) = \begin{bmatrix} \sigma_0^2 & 0 & \cdots & 0 \\ 0 & \sigma_1^2 & & 0 \\ \vdots & & \ddots & \vdots \\ 0 & 0 & \cdots & \sigma_m^2 \end{bmatrix} \tag{26}$$

Moreover, R is the redundancy matrix that conveys the relations between all the measured reaction fluxes. A zero column in R indicates that the other measured rates do not have significant expression for the correspond-

ing measured flux, i.e., it is not balanceable. Thus, the columns of matrix R can be inspected to identify the columns of the matrix R that can be used to distinguish between the balanceable reaction rates from the non-balanceable ones. Hereupon, the redundancy matrix can be expressed as [der Heijden et al., 1994]:

$$R = S_m - S_c {S_c}^{+} S_m \tag{27}$$

However, the redundancy matrix contains redundant equations related to the measured reaction rates. If the linearly dependent rows of R are removed, a reduced redundancy matrix (R_r) is obtained with rank(R) independent equations, that must be satisfied to calculate the nom-measured rates from the measured rates.

Finally, the non-measured reaction rates can be estimated through the substitution of $\hat{v_m}$ from equation 24 into equation 23:

$$v_c = -((S_c {S_c}^T)^{-1} S_c) S_m (S_c \Psi^{-1} {S_c}^T)^{-1} S_c \Psi^{-1} v_m \tag{28}$$

4.2.4 Null space

This method handles all the types of systems that can be configured from the stoichiometric metabolic model and the input data. It is based on the nullspace of the extended stoichiometry matrix (S_τ) that corresponds to the coefficients of the reactions on the metabolite balance equations of the metabolites and the coefficients of the reactions on the equality metabolic flux ratios.

As it has been shown on Eq 2 the metabolite balance is a system of linear equations that forces the rates of consumption and production of a metabolite

to be equal. The most trivial solution to this system is the zero vector which would represent the thermodynamic equilibrium. However, this is not the interesting solution when analysing flux distributions. Since the cell must have degrees of freedom due to the much higher number of reactions in its biochemical networks representation relating to the nodes (metabolites), several different flux distributions usually fulfil the system of Eq 2.

Algebraically, all possible solutions of the system are encompassed in the null space of S_τ [Strang, 1980]. Eq. 2 has $n - rank(S)$ linearly independent solutions (which define the dimension of the null space) that can be organized in a kernel matrix (K) that encompasses the base vectors that span the null space of S_τ. Therefore, the flux distributions into the null space of S_τ can be configured by linear combinations (b) of the columns in (K) as shown in:

$$v = K\beta \tag{29}$$

where K is an $n \times r$ matrix, where n defines the number of fluxes in the model and r is equal to the difference between n and the rank of the stoichiometric matrix. Also, β defines a column vector with r positions, each corresponding to the free variables of the system.

To find the null space of S_τ, the reduced echelon form of S_τ ($ref(S_\tau)$) is obtained. The solution for $ref(S_\tau)\ v = 0$ is the same as for the systems $S_\tau v = 0$ and $Uv = 0$ (U is the upper triangular factor obtained from LU decomposition). Here, the free variables are defined as the variables that do not correspond to columns in $ref(S_\tau)$ with pivot entries (the first non zero entries in their rows). To assign general solutions to $ref(S_\tau)\ v = 0$ (and consequently, to the Eq. 2), one may assign arbitrary values to the free variables. Accordingly, there are r-infinity solutions where the complete solution set is a combination of the special solutions corresponding to the free and independent variables:

$$v = v_{f,1} \begin{bmatrix} c_{1,0} \\ c_{1,1} \\ \vdots \\ c_{1,n} \end{bmatrix} + v_{f,2} \begin{bmatrix} c_{2,0} \\ c_{2,1} \\ \vdots \\ c_{2,n} \end{bmatrix} + \cdots + v_{f,k} \begin{bmatrix} c_{k,0} \\ c_{k,1} \\ \vdots \\ c_{k,n} \end{bmatrix} \tag{30}$$

where v is an $n \times 1$ column vector corresponding to the reaction fluxes, $v_{f,i}$ is the free variable i (the ith position on the vector β in Eq. 29) and each vector c_i multiplied by the free variables corresponds to the i special solution, i.e., the ith base vector of the null space of S_τ.

Moreover, usually the kernel matrix is not unique and not every qualitative different flux distribution might be represented by the base vectors in K [Szallasi et al., 2006]. Nevertheless, the kernel matrix can be used to detect strictly detailed balanced reactions, if the corresponding rows have only zero values [Heinrich and Schuster, 1996]. Also, the occurrence of coupled/correlated reaction sets, that represent a set of reactions that operate with a fixed ratio in their rates [Pfeiffer et al., 2001], such as reactions in the same linear pathway, can be identified from the null-space matrix, since the rows of the reactions will only differ by a scalar. Moreover, in the presence of a system whose reactions are completely disconnected or its fluxes are not related to the other fluxes in the network, sub-networks can be identified if the kernel is block-diagnosable [Heinrich and Schuster, 1996, Szallasi et al., 2006].

Thus, although a single point in the null space of S_τ is commonly not possible to be obtained to translate a solution that reflects the flux distribution of a given experiment, due to the more likely occurrence of undetermined systems, the null space of the stoichiometric matrix can be used to characterize the configured systems.

After obtaining the null space of the stoichiometric matrix (K), the number of unknowns of the system is said to be equal to the number of base

vectors in K (number of free variables in Eq. 29). A new matrix (K_m^T) is obtained where its columns refer to the lines in K that correspond to measured fluxes. Therefore, the number of rows and columns in K_m^T will be equal to the number of unknowns and measurements, respectively. This matrix will be decomposed with LU decomposition and its upper triangular factor is retrieved and stored in a matrix U and its lower triangular factor is stored in a matrix L. Since the decomposition might need to perform some row and column permutations, these are stored to map the rows and columns to the original indexes.

The rank of K_m^T (r_{K_m}) is always less or equal to the number of measurements and less or equal to the number of unknowns. Some conclusions can be made if r_{K_m} is not equal to the number of unknowns:

- the matrix K_m^T has a null space;
- K_m^T is rank deficient, and its rank deficiency is equal to the difference between the number of unknowns and its rank;
- there exists a number of linearly independent solutions equal to the rank deficiency plus one.

Principal flux distribution

To deal with the rank deficient case, a new smaller system that contains a unique solution is defined. The r_{K_m}-first rows of the upper factor of K_m^T are copied to a new matrix U_s, and an invertible leading matrix ($U_{leading}$) is defined by the r_{K_m}-first linearly independent rows of the transposed matrix of U_s, and the vector β of the free fluxes in Eq. 29 is obtained after solving the following systems:

$$U_{leading}\gamma_s = v_{ms} \tag{31}$$

$$L^T y = \gamma \tag{32}$$

where v_{ms} is a vector with the r_{K_m}-first measured fluxes, with the original indexes permuted to the K_m^T after its LU decomposition. The vector γ_s in Eq 31 is obtained through forward substitution, solving the lower triangular system, and y is obtained from back substitution by solving the upper triangular system. The number of elements of γ is equal to the number of unknowns and its r_{K_m}-first positions are obtained from the vector v_{ms}. The vector β is finally obtained by indexing the elements in y with respect to the permutations performed during the LU decomposition of K_m^T. Note that the indexes of the rows in K_m^T matches the row indexing in K and therefore, Eq. 29 can be used to compute the values of the fluxes.

However, before computing the vector y, a verification is done relating to the vector γ_s. From this vector, the measured fluxes are computed from the above equation bellow, and afterwards compared with the actual measured fluxes (v_m).

$$v_{m,computed} = U_s^T.\gamma_s \tag{33}$$

If the sum of the module of the difference between the elements in $v_{m,computed}$ and the elements in v_m is greater than a given value ϵ, the vector γ_s is recomputed by least squares approximation:

$$U_s U_s^T \gamma_s = U_s v_m \tag{34}$$

Alternative flux distributions

If K_m^T is rank deficient, alternative solutions are computed. Therefore, the number of unknowns and the rank of the matrix K_m^T (r_{K_m}) will dictate the

number of alternative solutions of the system. As it was stated before, the difference between these values reflects the rank deficiency of the system and the method will return rank deficiency+1 alternative solutions. To calculate these solutions, an iterative process is performed by initializing a new vector of zeros for γ and giving the value one to each position, starting from the index r_{K_m}. Thus, in each iteration γ, we will not have a zero value in only one position, and this is used to obtain the β vector and posteriorly to calculate the alternative flux distribution, as in the calculation of the principal flux distribution, through the equations 32 and 29. Each value obtained in this step is afterwards summed to the corresponding flux value in the previously obtained principal solution.

References

[Bonarius et al., 1997] Bonarius, H. P. J., Schmid, G., and Tramper, J. (1997). Flux analysis of underdetermined metabolic networks: the quest for the missing constraints. *Trends in Biotechnology*, 15(8):308–314.

[der Heijden et al., 1994] der Heijden, R., Heijnen, J. J., Hellinga, C., Romein, B., and Luyben, K. (1994). Linear constraint relations in biochemical reaction systems: I. Classification of the calculability and the balanceability of conversion rates. *Biotechnology and bioengineering*, 43(1):3–10.

[Edwards and Palsson, 2000] Edwards, J. S. and Palsson, B. O. (2000). Robustness analysis of the escherichiacoli metabolic network. *Biotechnology Progress*, 16(6):927–939.

[Fischer et al., 2004] Fischer, E., Zamboni, N., Sauer, U., and Others (2004). High-throughput metabolic flux analysis based on gas chromatography-mass spectrometry derived 13C constraints. *Analytical biochemistry*, 325(2):308.

[Heinrich and Schuster, 1996] Heinrich, R. and Schuster, S. (1996). *The regulation of cellular systems*, volume 416. Chapman & Hall New York.

[Lewis et al., 2010] Lewis, N. E., Hixson, K. K., Conrad, T. M., Lerman, J. A., Charusanti, P., Polpitiya, A. D., Adkins, J. N., Schramm, G., Purvine, S. O., Lopez-Ferrer, D., and Others (2010). Omic data from evolved E. coli are consistent with computed optimal growth from genome-scale models. *Molecular systems biology*, 6(1).

[Madron et al., 1977] Madron, F., Veverka, V., and Vaněček, V. (1977). Statistical analysis of material balance of a chemical reactor. *AIChE Journal*, 23(4):482–486.

[Mahadevan et al., 2003] Mahadevan, R., Schilling, C. H., and Others (2003). The effects of alternate optimal solutions in constraint-based genome-scale metabolic models. *Metabolic engineering*, 5(4):264.

[McAnulty et al., 2012] McAnulty, M. J., Yen, J. Y., Freedman, B. G., and Senger, R. S. (2012). Genome-scale modeling using flux ratio constraints to enable metabolic engineering of clostridial metabolism in silico. *BMC systems biology*, 6(1):42.

[Palsson, 2006] Palsson, B. O. (2006). Systems biology: properties of reconstructed networks.

[Papoutsakis, 1984] Papoutsakis, E. T. (1984). Equations and calculations for fermentations of butyric acid bacteria. *Biotechnology and bioengineering*, 26(2):174–187.

[Penrose, 1955] Penrose, R. (1955). A generalized inverse for matrices. In *Mathematical Proceedings of the Cambridge Philosophical Society*, volume 51, pages 406–413.

[Pfeiffer et al., 2001] Pfeiffer, T., Schuster, S., and Bonhoeffer, S. (2001). Cooperation and competition in the evolution of ATP-producing pathways. *Science*, 292(5516):504–507.

[Sauer et al., 1999] Sauer, U., Lasko, D. R., Fiaux, J., Hochuli, M., Glaser, R. W., Szyperski, T., Wüthrich, K., and Bailey, J. E. (1999). Metabolic flux ratio analysis of genetic and environmental modulations of Escherichia coli central carbon metabolism. *Journal of bacteriology*, 181(21):6679–88.

[Segre et al., 2002] Segre, D., Vitkup, D., and Church, G. M. (2002). Analysis of optimality in natural and perturbed metabolic networks. *Proceedings of the National Academy of Sciences*, 99(23):15112–15117.

[Stephanopoulos et al., 1998] Stephanopoulos, G., Aristidou, A. A., Nielsen, J. H. i., and Nielsen, J. (1998). *Metabolic engineering: principles and methodologies*. Academic Press.

[Strang, 1980] Strang, G. (1980). Linear Algebra and its Applications.

[Szallasi et al., 2006] Szallasi, Z., Stelling, J., and Periwal, V. (2006). *System modelling in cellular biology*. MIT Press, Cambridge, MA.

[Wang and Stephanopoulos, 1983] Wang, N. S. and Stephanopoulos, G. (1983). Application of macroscopic balances to the identification of gross measurement errors. *Biotechnology and bioengineering*, 25(9):2177–2208.
