# Supplementary material for: CBFA: phenotype prediction integrating metabolic models with constraints derived from experimental data
Source: BMC Syst Biol. 2014 Dec 3;8:123. doi: 10.1186/s12918-014-0123-1 (PMC4263207; doi:10.1186/s12918-014-0123-1)
Supplement: Additional file 3: — CBFA tutorial. [file 12918_2014_123_MOESM3_ESM.pdf]

CBFA
Tutorial

CBFA is an open-source software created by BiSBII (Bioinformatics and Systems Biology Interdisciplinary Initiative), a joint initiative by

> **IBB/CEB** – Institute for Biotechnology and Bioengineering Centre of Biological Engineering (University of Minho)
> **CCTC** - Computer Science and Technology Center (University of Minho)

List of acronyms

FBA Flux Balance Analysis

pFBA Parsimonious enzyme usage Flux Balance Analysis

FVA Flux Variability Analysis

LP Linear Programming

QP Quadratic Programming

Contents

1 General overview of the application **2**

2 Configuring the solver to be used **3**

3 Creating a project **4**
- 3.1 Step 1 - Project name and format 5
- 3.2 Step 2 - Model file selection 6
- 3.3 Step 3 - Drains and external metabolites 6
- 3.4 Step 4 - Biomass growth 8

4 Creating environmental conditions **9**

5 Experimentally measured fluxes **10**
- 5.1 Create measured fluxes 11
- 5.2 Import measured fluxes 14

6 Metabolic flux ratios **16**
- 6.1 Creating flux ratios 16
- 6.2 Importing flux ratios 20

7 Performing flux analysis with underdetermined systems **23**
- 7.1 Flux balance analysis approach 25
- 7.2 Parsimonious Flux balance analysis approach 29
- 7.3 Tight bounds approach 30
- 7.4 Flux Variability Analysis approach 32
- 7.5 Quadratic Programming approach 33

8 Robustness analysis **36**

9 Performing flux analysis for mutant simulation **38**
- 9.1 Mutant flux analysis - reaction deletions 38
- 9.2 Mutant flux analysis - gene deletions 40

10 Solving the systems algebraically **42**

11 Performing null space-based flux analysis **46**

12 Visualizing the flux distribution of the flux analysis methods **48**

1 General overview of the application

The first step, if you haven't already installed the *CBFA* software, is to install the OptFlux framework. For that, please refer to the instructions on the website `http://www.optflux.org`. This plug-in is fully available in the standard OptFlux installation in the most recent versions[1]. You should read the general OptFlux tutorial to understand better how to work with the main concepts of the framework user interface.

After launching the software you will be presented with the layout depicted in the Figure 1 below. Most of the *CBFA* main features and operations will be accessible to you either through the **Menu** or the **Toolbar**. Your objects i.e., the project, metabolic models, environmental conditions, experimentally measured fluxes, flux analysis results, etc., will always be placed in the **Clipboard** area on the left. These objects are organized by their data types that determine the way they can be visualized and also the specific operations that can be launched (available through the context menu obtained right-clicking specific objects in the clipboard). The **Visualization Area** is the area where you can examine the contents of those objects in detail. When you click on a data type, the different views for that object will be available in this area. If more than one view exists you can select the active view through the tabs in the bottom.

Click around to get familiar with it and, after that, jump to the next step. In this document, we will cover the major operations of *CBFA*, their configurations and results. Over the next sections, the configuration of experimental inputs, the different alternatives to perform flux analysis simulations and their visualization will be addressed in detail.

[1]If your version is older (lesser or equal than 3.0.6) please use the OptFlux update features to make sure you have the most recent versions of all plug-ins and, in this case, explicitly install the *CBFA* plug-in

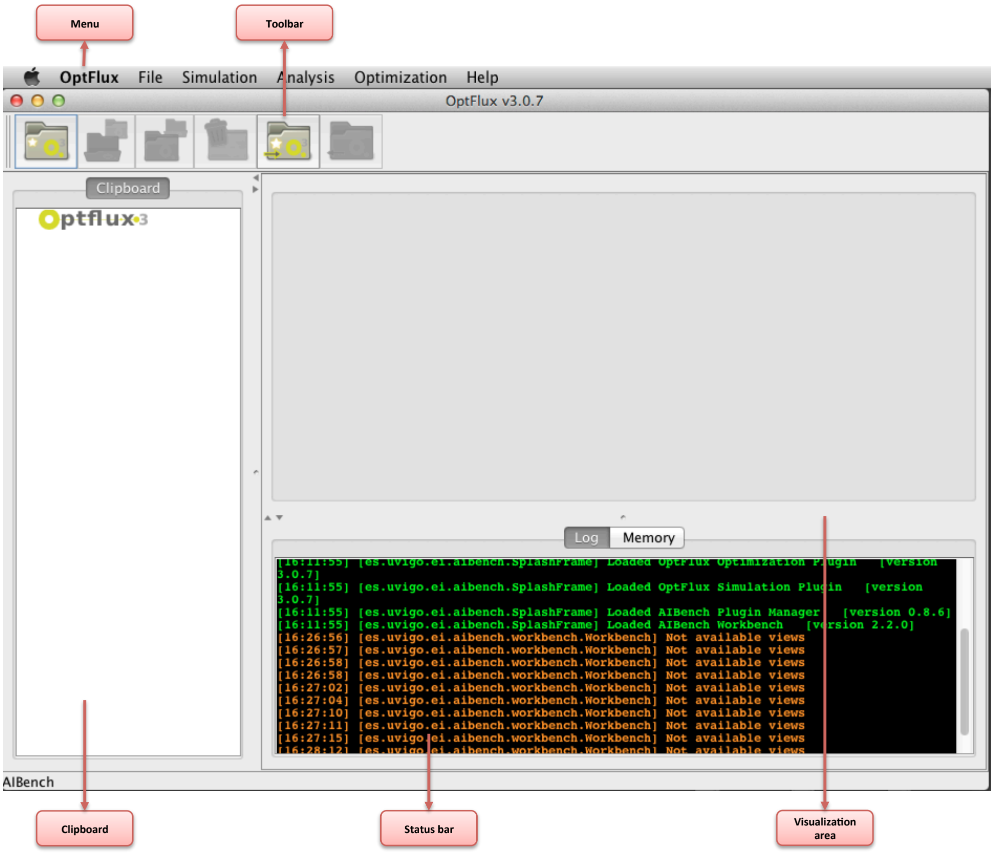

Figure 1: General view of the software's interface

2 Configuring the solver to be used

For the optimization methods, *CBFA* provides the possibility to choose between three solvers:

- *GNU Linear Programming Kit* (GLPK);
- *IBM ILOG CPLEX*;
- Coin-or linear programming (CLP);

To configure the solver to be used in the optimizations, select the **Preferences** option available through the *File / Help* menu (Figure 2).

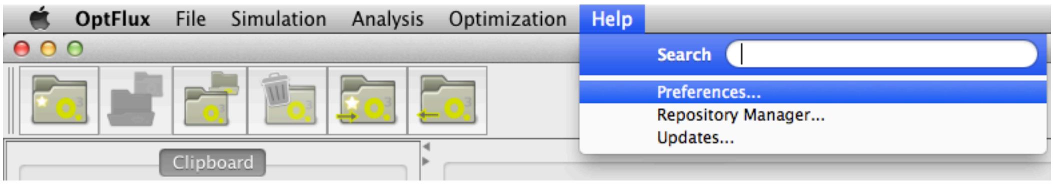

Figure 2: Solver selection - menu option

After selecting the option "Simulation" on the left panel of the interface that is prompted, the available solvers are made accessible on the right-hand combo boxes (Figure 3). Here, the user should check the solvers for linear programming and Quadratic Programming (QP). If the user has access to CPLEX, this should be the chosen one (note that in this case, OptFlux needs to restart for the configuration to take effect). For QP tasks, GLPK cannot be used.

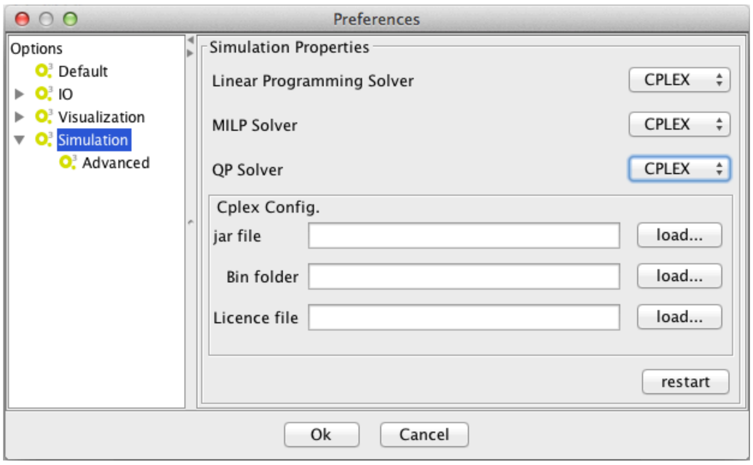

Figure 3: Solver selection - operation dialogue

3 Creating a project

To begin the creation of a new project, you have to start the **New Project** wizard available through the *File Menu* or the *Toolbar*.

It is possible to create a new project from several different sources / formats. However, in this tutorial, we will cover only the option to create a project from a file in the SBML format. To check how to create a project from a different format, please refer to the *Optflux 3*'s *how to's* pages in `http://www.optflux.org`.

Thus, all the operations shown in this document will be performed over SBML-formatted models. SBML is an XML dialect created for the representation of Systems Biology models. Check the site `www.sbml.org` to learn more about this format. To follow the steps in this section, you need to download the file *ecoli-core-model.xml*, available from `http://www.optflux.org/cbfa/ecoli-core-model.xml` and save it to a directory of your choice. This is a simplified model of the metabolism of *Escherichia coli*, proposed in [Orth et al., 2010]. It is also available in the web site `http://gcrg.ucsd.edu/Downloads/EcoliCore`.

The creation of a project from an SBML file is accomplished in four steps listed below.

3.1 Step 1 - Project name and format

After you have started the **New Project** wizard, you will be prompted with a panel where you have to type the name of the project you will be working with. This name has to be different from the projects that already exist on the **Clipboard**. Also, you have to choose the format of the model that you want to load (in this case the SBML format) as shown in Figure 4. After that, click on *Next*.

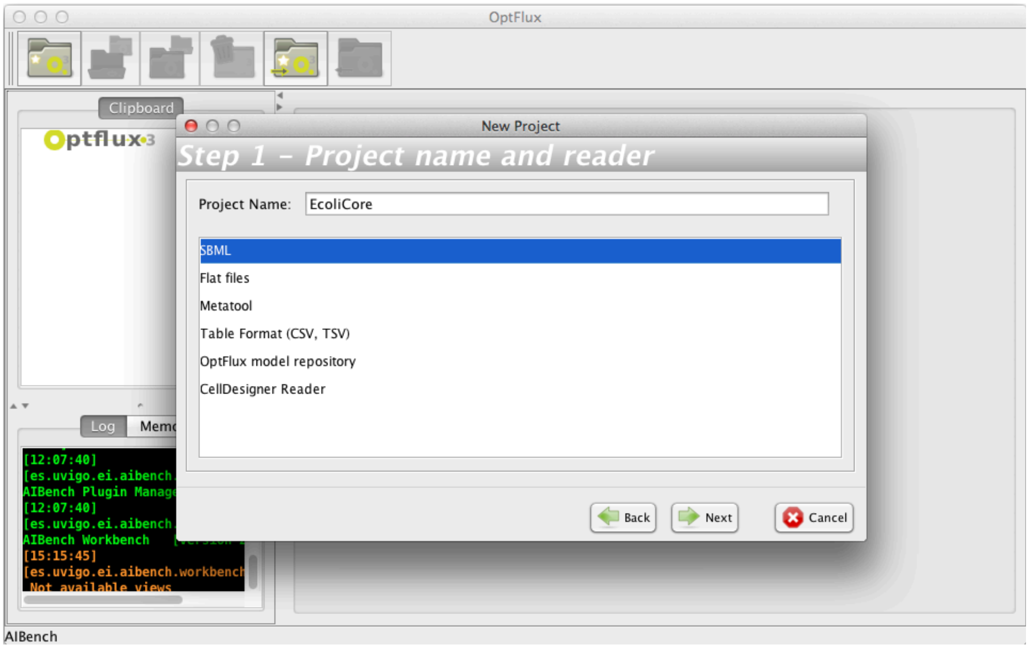

Figure 4: Create project - file format selection

3.2 Step 2 - Model file selection

In the second step, you must select the SBML file to load (Figure 5). Click on the *Find* button, search for the file and click on *Open*. In this example, the file is the one provided as stated above (*ecoli-core-model.xml*). Click on *Next*.

3.3 Step 3 - Drains and external metabolites

The third step is relative to the extra-cellular environment and identification of drains (Figure 6). This provides a set of options depending on the file format. *CBFA* will automatically try to find the appropriate method using heuristics. In this case, it will propose to identify external metabolites using a regular expression, by considering all metabolites whose id ends with "_b". These will be removed from the model. In this case, this is indeed the best

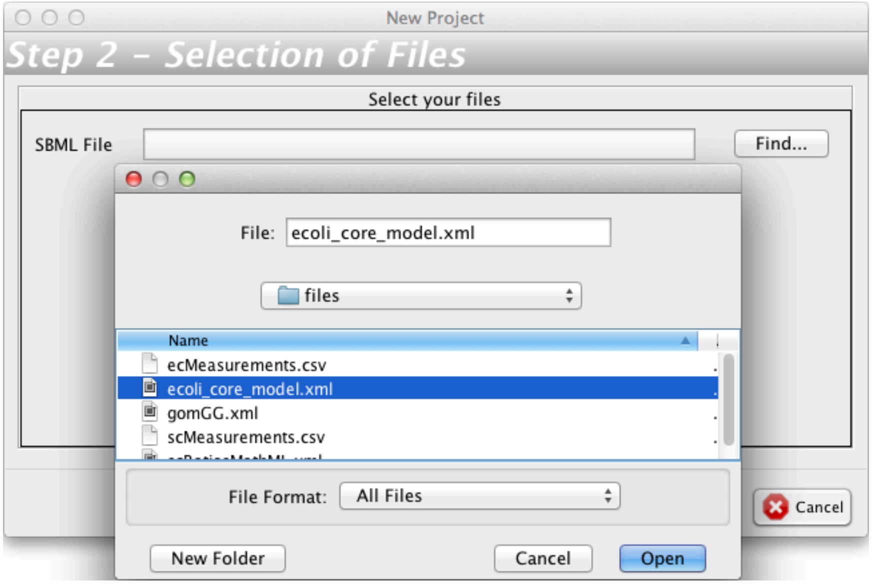

Figure 5: Create project - model file selection

option, and therefore you can accept *CBFA*'s suggestion and proceed. Click on *Next* for the last step.

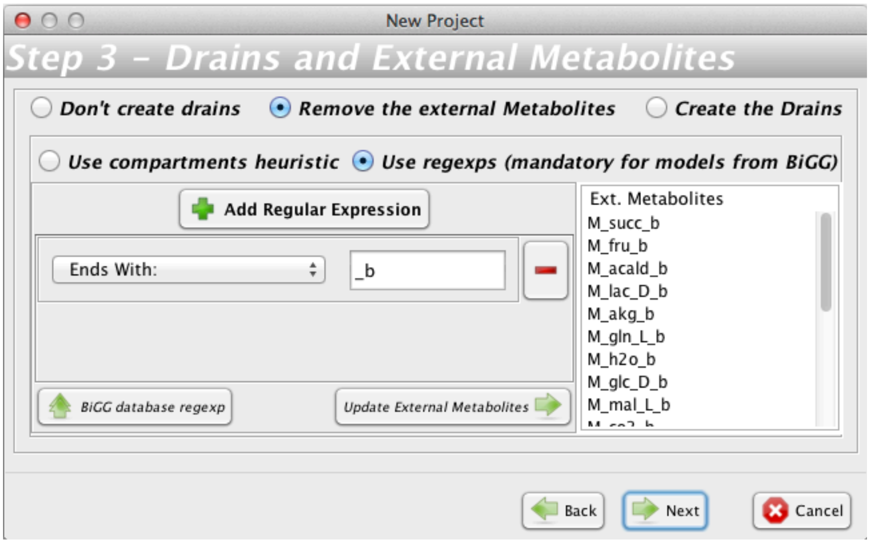

Figure 6: Create project - drains and external metabolites configuration

3.4 Step 4 - Biomass growth

In the fourth step, *CBFA* automatically tries to find the biomass growth associated flux (Figure 7), since this information is essential for the simulation procedures. A heuristic method will automatically identify the appropriate reaction as it is easy to check. Click on *Finish* to conclude the project creation.

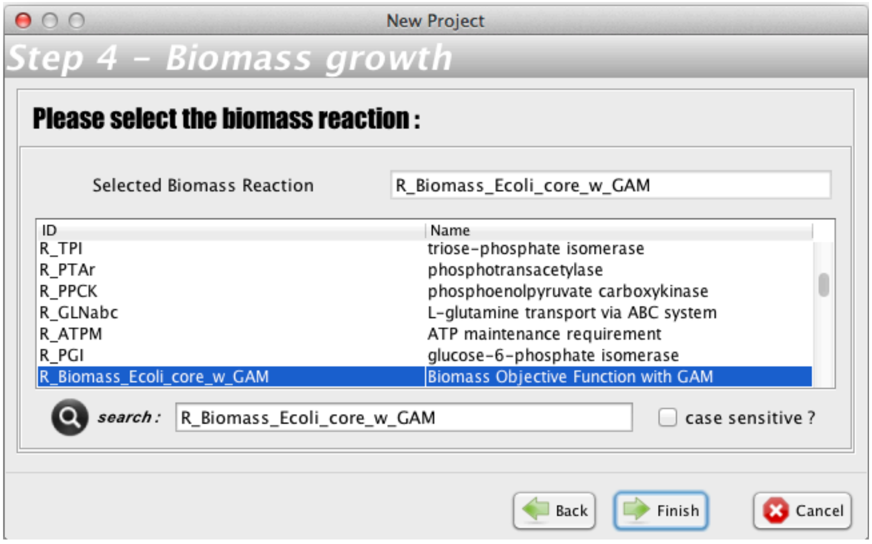

Figure 7: Create project - selection of the biomass reaction

After these steps, you can easily check that a new instance is presented on the **Clipboard** that corresponds to the project that you have created (Figure 8). As you can see, an object of the Project data type is a nested object that contains other nested data types. The core data type is named "Metabolic Model. Within this data type, one can access information about reactions (internal and drains), metabolites and also the stoichiometric coefficients in a human-readable fashion. Also, genes and gene rules (specifying gene-rule associations) are shown if the model contains such information. Because all operations are performed in the context of a given project, every data type that is generated as an output of an operation will be attached to the project. It is also important to notice that a project contains a single model that will be the core of this project, being used in all subsequent operations.

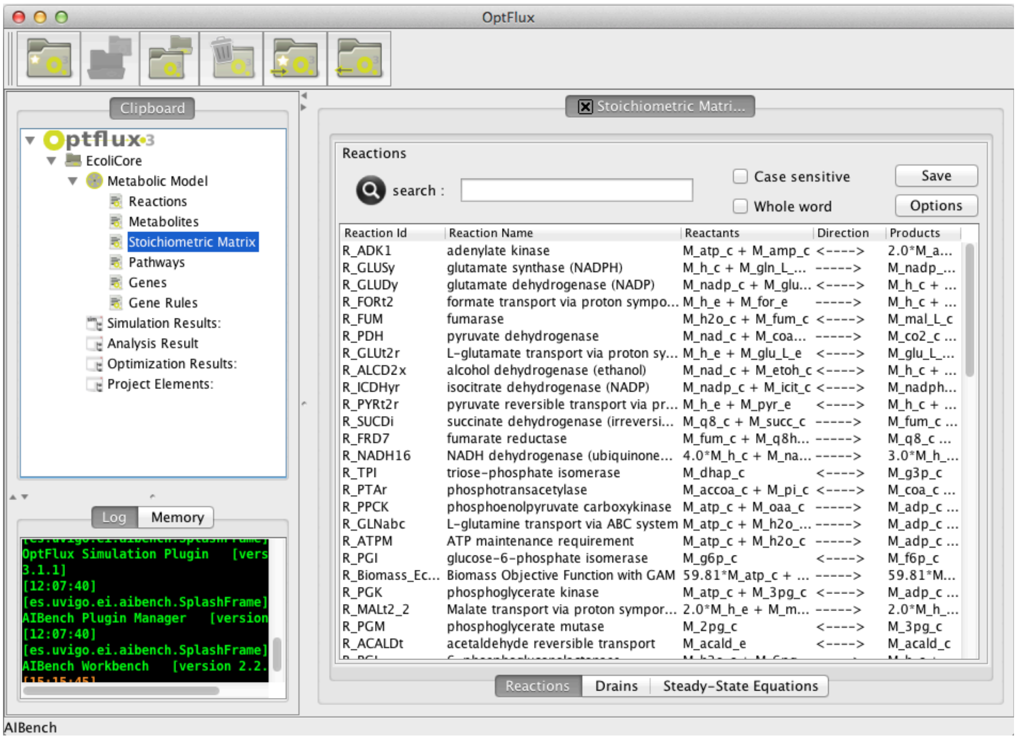

Figure 8: Project data type view

4 Creating environmental conditions

Environmental conditions can be used to define the bounds of uptake fluxes, i.e. the rates at which metabolites are consumed from the medium. Thus, these objects can be used to define media for the growth of the cells. Environmental conditions are created in the option **Environmental Conditions** available through the *File / Create* menu, as shown in Figure 9.

The interface allows you to select drain reactions and define their limits. First, you have to select the project in which the environmental conditions should be created. If there is more than one project on the clipboard, you can click on the combo box on the *Select Project* panel and select the desired project. This panel is common to every operation in this software, since, as stated before, all the tasks are performed over the core model in the project.

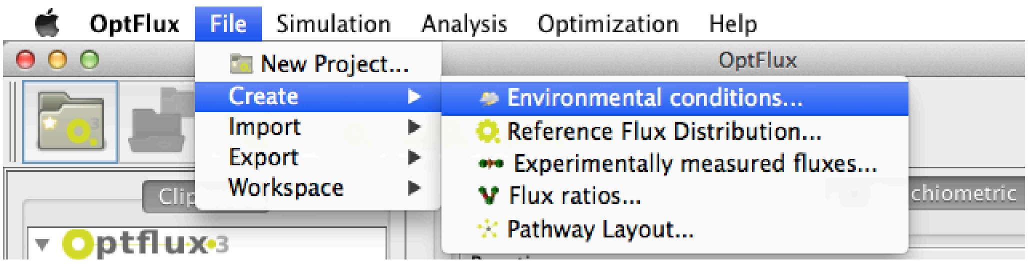

Figure 9: Environmental conditions definition - menu option

Whenever you change the project, the information that is project specific will be updated in the operation panels.

The upper part allows the selection of the reactions (easing its search), while the bottom part allows to add these constraints to the environmental condition, specifying the lower and upper bounds. The list in the bottom part of the interface keeps the reactions and their limits as they are added to the environmental conditions. In the example, the environmental condition will define anaerobic conditions (since the lower limit for oxygen uptake is 0) and the maximum uptake rate for glucose is set to 20, instead of the original 10 (Figure 10).

The object corresponding to the configured environmental conditions is added to the project in the clipboard, under the general data type "Project Elements" as illustrated in Figure 11. Here, every instance of an environmental condition data type is grouped and numbered by the order by which it is created. Again, if you select the object you have just created, a view will be displayed on the visualization area, as shown in Figure 11.

5 Experimentally measured fluxes

If you want to define more precise bounds, found from experimental data, on the rates of formation and consumption of some compounds involved in the

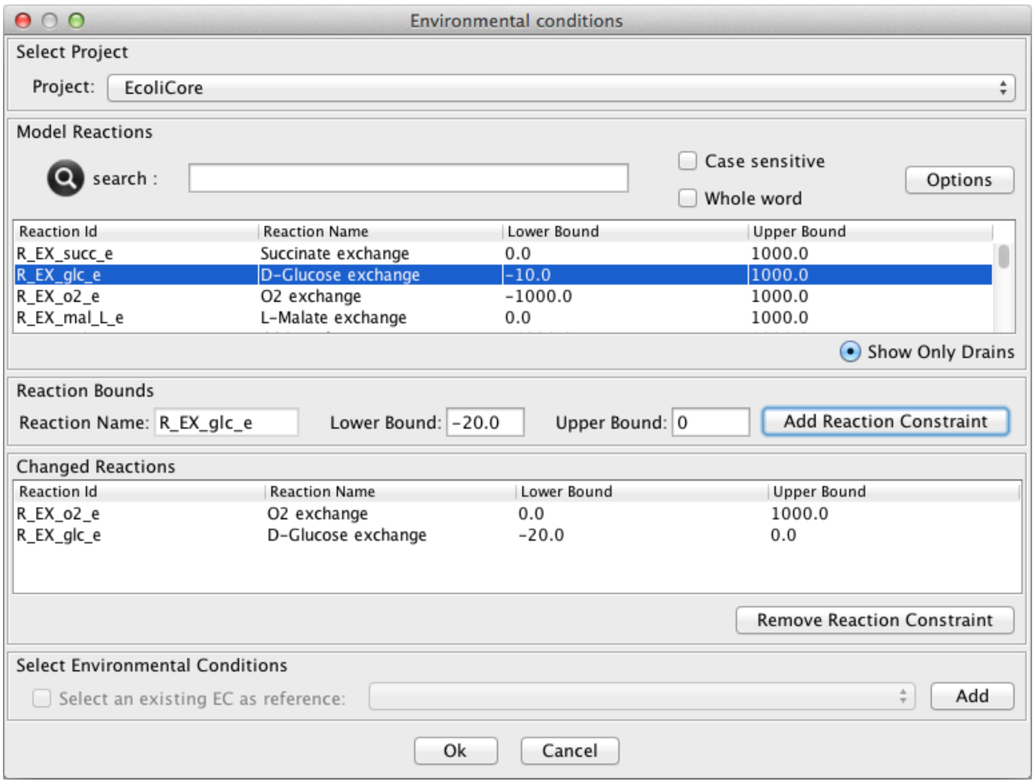

Figure 10: Environmental conditions definition - operation dialogue

network, such as extracellular substrates (e.g. glucose), it is possible to set up flux measurements through the operation **Experimentally measured fluxes** available on the *File / Create* menu (Figure 12). This operation allows to set the fluxes to have a certain measured value or to respect a range (e.g. computed from measured values with available errors).

5.1 Create measured fluxes

First, as in the creation of environmental conditions, select the project in which you want to add the flux measurements.

In this tutorial, although we have defined the glucose uptake bounds, we are going to force the glucose (id *R_EX_glc_e*) uptake rate to be a fixed value

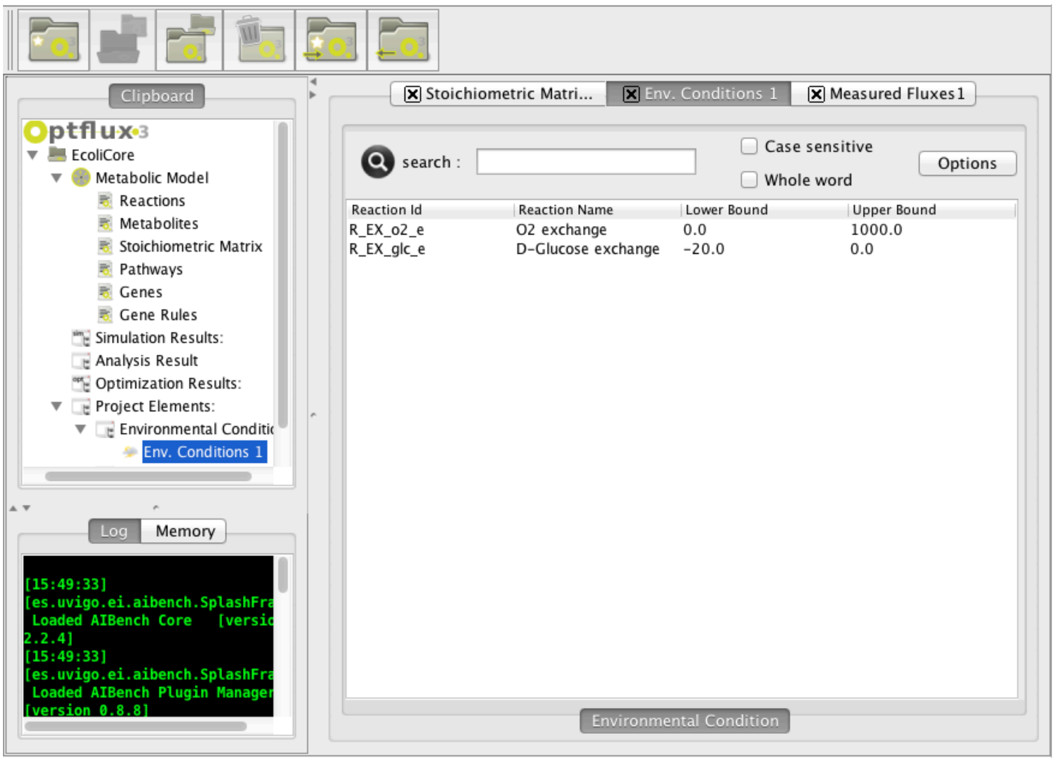

Figure 11: Environmental conditions - data type view

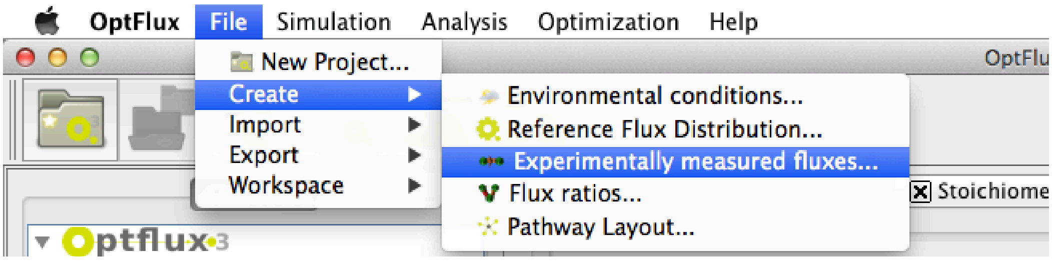

Figure 12: Measured fluxes operation - menu option

equal to -20. Since the list of reactions in the model can be extensive, you can search for the reaction and the table will be filtered to show only the reactions names that match the typed expression. Now, let us define two more flux measurements (*R_EX_mal_L_e* and *R_EX_pyr_e* that will be set to 0) as shown in Figure 13.

Let's assume that there are no replicates for the flux measurements. Thus,

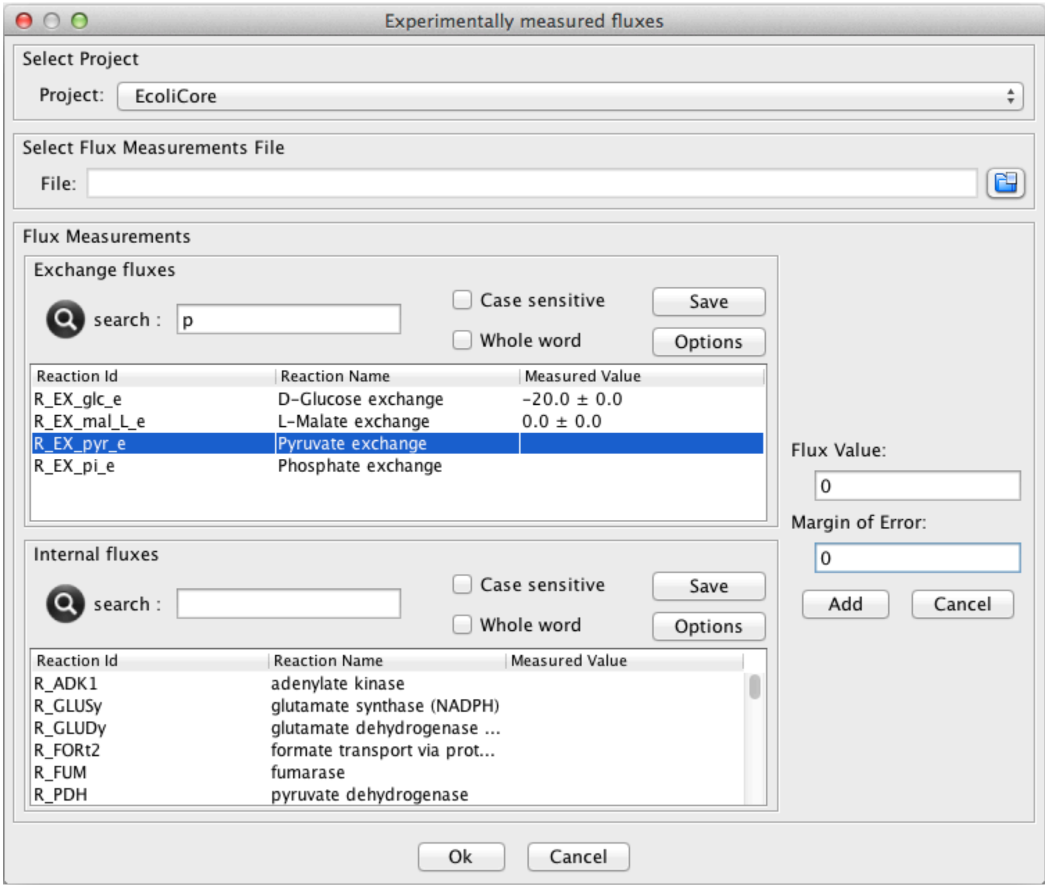

Figure 13: Create measured fluxes - operation dialogue

when you select the row on the table that corresponds to the reaction with a measurement, two boxes are presented on the right side of the panel where it is possible to set the flux value and the margin of error or confidence interval (0 in this case). After setting the values for the three fluxes referred above, click on the *Ok* button, and the object with the measured fluxes is added to the list of objects with data type “Measured Fluxes” in the clipboard under the “Project Elements” (Figure 14).

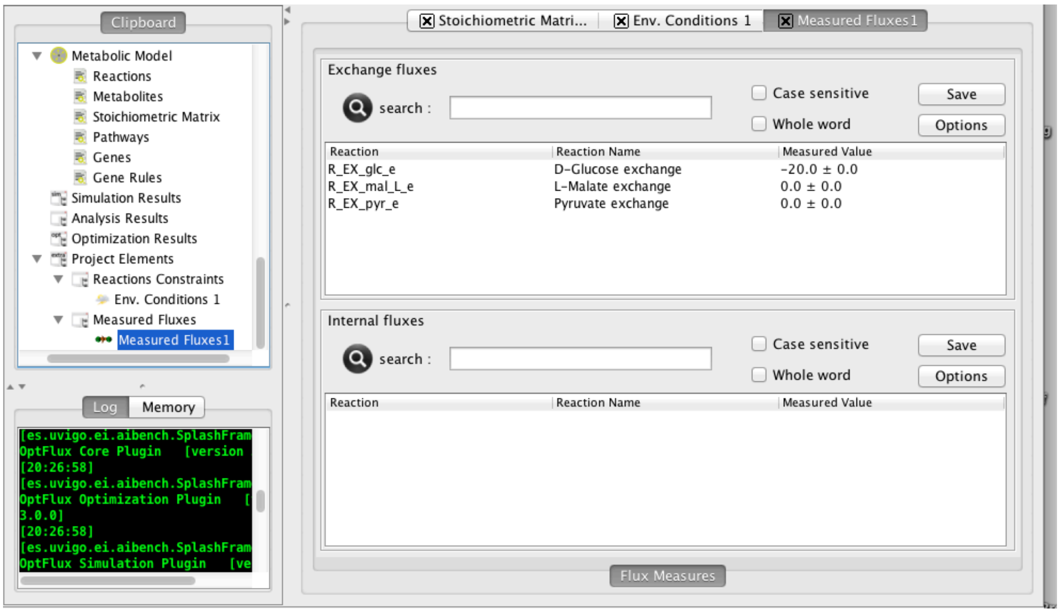

Figure 14: Created measured fluxes - data type view

5.2 Import measured fluxes

To import a set of flux measurements, click on the button for searching files and select the file with the measurements. Such files should be in the CSV (comma-separated values) format and you can select the proper column separator on the top of the dialogue (Figure 15). As you can check, there are two radio buttons to select the type of file:

- *Flux values list*: the first column corresponds to the reaction id, and a set of replicate measurements of the flux are given in the remaining columns (if there is only one measurement, each row will contain only two columns);
- *Flux value and margin of error*: the fist column identifies the reaction, the second column is the mean of the flux measurements and the third column contains the margin of error for the flux.

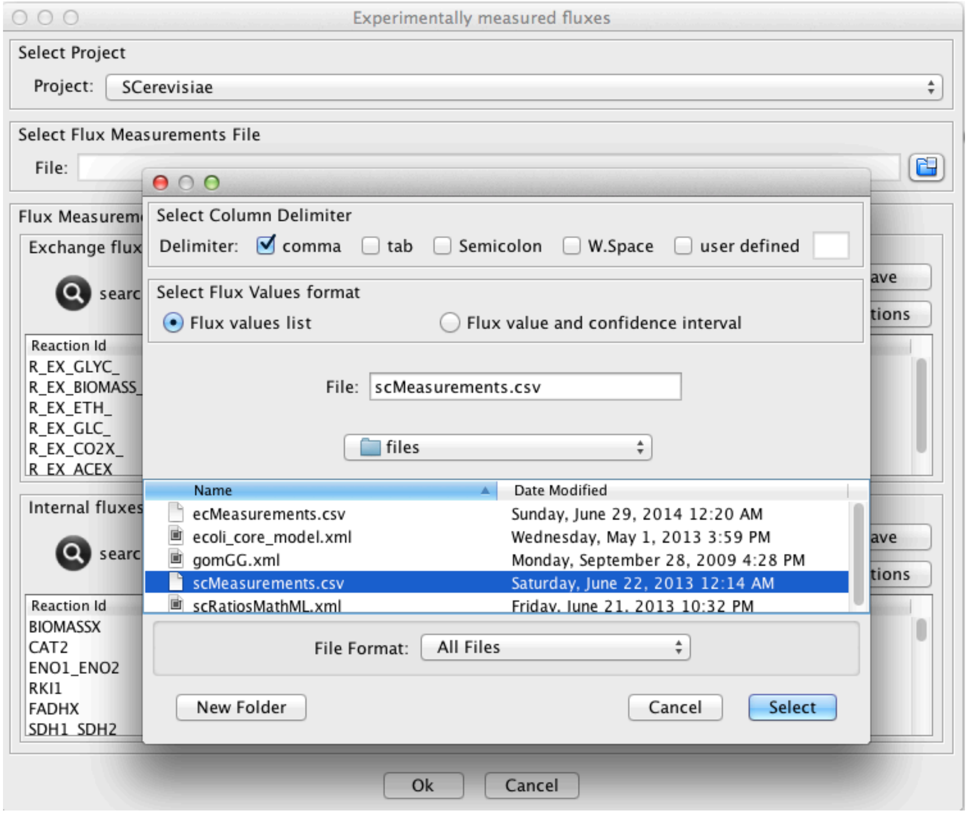

Figure 15: Import measured fluxes - operation dialogue

In this example we are going to import a file of the first type, with the columns separated by the comma character, where there is only one measurement of the fluxes (please download the file in `http://www.optflux.org/cbfa/scMeasurements.csv`). After selecting the file, the table with the flux list will be updated with the uploaded measurements.

The result of the operation is added as an object to the clipboard with the id "Measured Fluxes2" under the "Measured Fluxes" list (Figure 16).

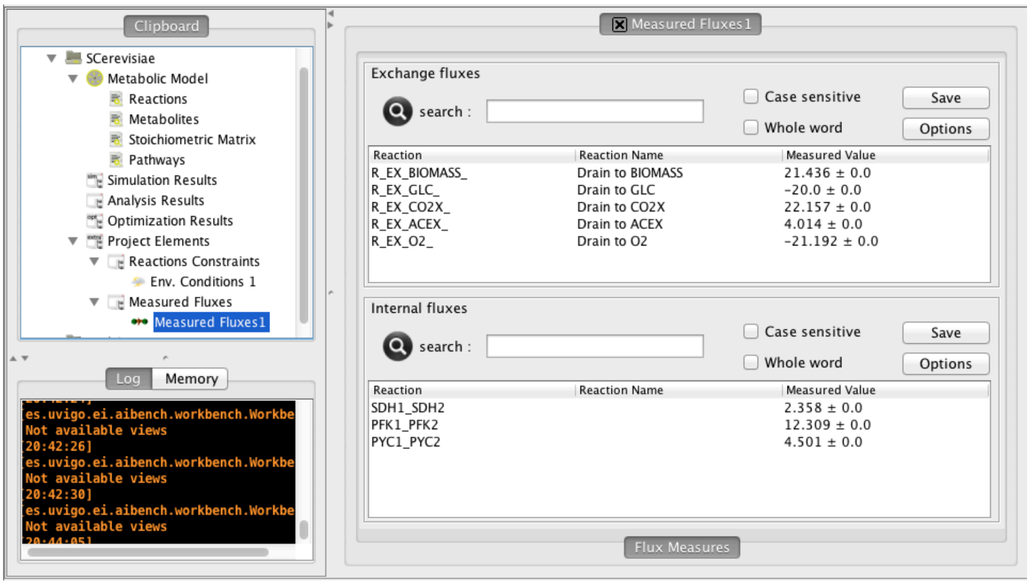

Figure 16: Imported measured fluxes - data type view

6 Metabolic flux ratios

It is also possible to set up metabolic flux ratios that are used as constraints to the flux analysis methods of the software. In order to do that, mathematical expressions can be defined using a set of flux values and converted afterwards by the software to its linear form. You can either create new flux ratios or import existing ones from raw text or MathML formatted files.

6.1 Creating flux ratios

In order to create a set of metabolic flux ratios, you must select the option **Flux ratios** from the *File / Create* menu (Figure 17).

The software accepts both equality and inequality ratio expressions which have the following format:

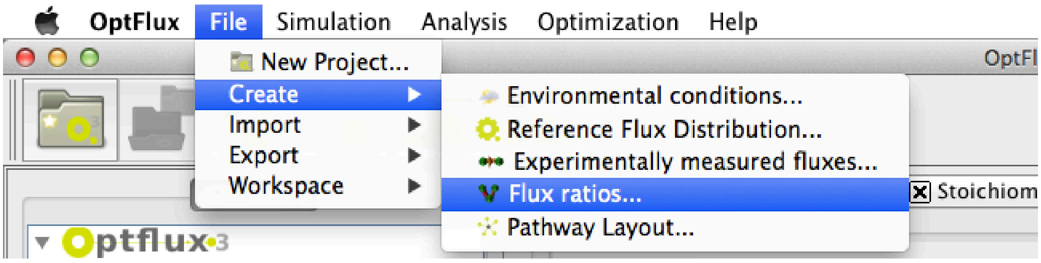

Figure 17: Create flux ratios operation - menu option

<numerator terms> / <denominator terms> <comparator> <ratio value>

The numerator and denominator terms represent sum and subtraction operations over fluxes. Note that it is assumed that every operation on each side is performed before the division (assume there are enclosing brackets surrounding the two expressions). Moreover, it is possible to add coefficients to the fluxes, by just adding a real number before the reaction id. The comparator can be either the equality or an inequality comparator, and the ratio value is a real number.

To illustrate this feature, lets create two flux ratios. The first one is an equality equation that translates the fraction of oxaloacetate originating from phosphoenolpyruvate given by:

$$\frac{R\_PPC}{R\_PPC + R\_MDH} = 0.7$$

where in the numerator there is the flux corresponding to the phosphoenolpyruvate carboxylase reaction and in the denominator there is the sum of the phosphoenolpyruvate carboxylase and malate dehydrogenase reaction rates.

The second ratio is an inequality expression that represents the upper bound for the fraction of oxaloacetate derived through the glyoxylate shunt:

$$\frac{R\_MALS}{R\_MALS + R\_FUM + R\_PPC - R\_ME1} \leq 0.35$$

where the numerator contains the malate synthase reaction (*R_MALS*) and the denominator is the sum of the fluxes through the malate synthase, phosphoenolpyruvate carboxylase (*R_PPC*) and fumarase (*R_FUM*) reactions, subtracted by the rate of conversion of the tricarboxylic acid intermediates into gluconeogenic precursors via malic enzyme (*R_ME1*).

In Figure 18, the interface to manually create this kind of expressions is illustrated. In this case, the first expression is already added to the "Flux Ratio Constraints" as you can see at the bottom-right panel. This list keeps the ratios that the user is creating, so a set of ratios can be created in the same operation.

When an expression is configured, it is shown on the "Flux Ratio Expression" panel. The reaction identifiers are searchable on the left-side panel and whenever a reaction is selected, it is added to the "Flux Ratio Expression". Also, if the selected reaction is a reversible reaction, the *Negative flux* button is made enabled, so it can be set to be negative, relative to the stoichiometric matrix, otherwise the button is not enabled. The operators are attached to the expression by clicking on the corresponding button at the operators panel, and the same applies to the comparator symbols. To add coefficients to the fluxes, or to set the ratio value, click on the digits button, and the operators panel will be switched to the digits panel and the decimal values can be configured, as shown in Figure 19. To show back the operators panel, you have to click again on the digits button.

It is always possible to undo some operations, returning to previous stages when building the expressions, so you do not need to start it all over again. It is also useful to add a description for the ratios that are being configured, so, although it is optional, you can do it on the *Description* text box. To update the *Flux Ratio Constraints* list with the ratio that you have configured, click

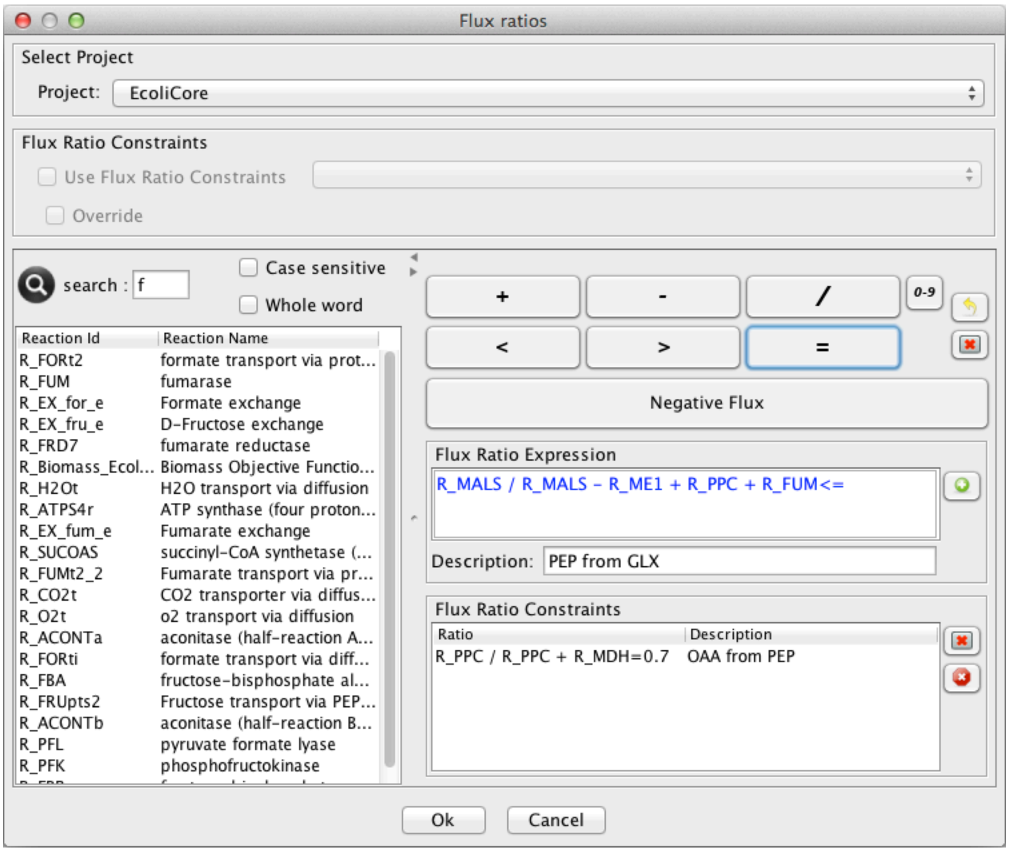

Figure 18: Create flux ratios - operation dialogue

on the *add* button. Here, if the expression does not respect the syntax stated above, it is not possible to add the ratio to the constraint list and a warning will be displayed. Only the ratio equations on the list will be created when the *Ok* button is selected. When it is done, a new object is added to the clipboard (Figure 20), under the project's 'Flux Ratio Constraints" list.

If you click on the created flux ratios object (named "Flux Ratio Constraint 1") you can check the ratios that you have configured on the visualization area, and also verify the linear form of the ratio equations.

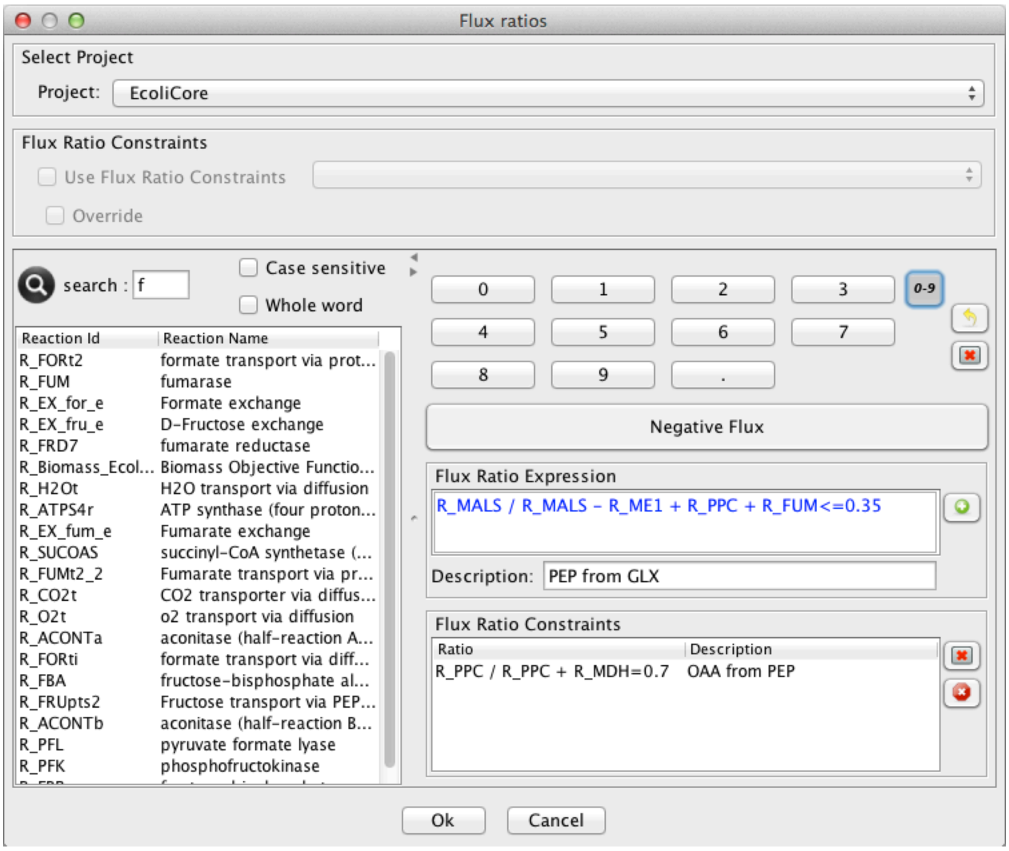

Figure 19: Create flux ratios operation - digits panel

6.2 Importing flux ratios

Now let's extend the flux ratios that we have created to encompass two more flux ratios that will be useful in posterior sections of this tutorial. But now, we are going to load the ratios from an existing file, so we do not need to configure them in the interface showed in the previous subsection. To select the proper operation, pick the option **Flux ratios** from the *File / Import* menu (Figure 21).

It is possible to load either text files with the raw expressions written, or in a more generic format for mathematical expressions, MathML (`http://www.w3.org/Math/`). In this example we are going to load flux ratios that

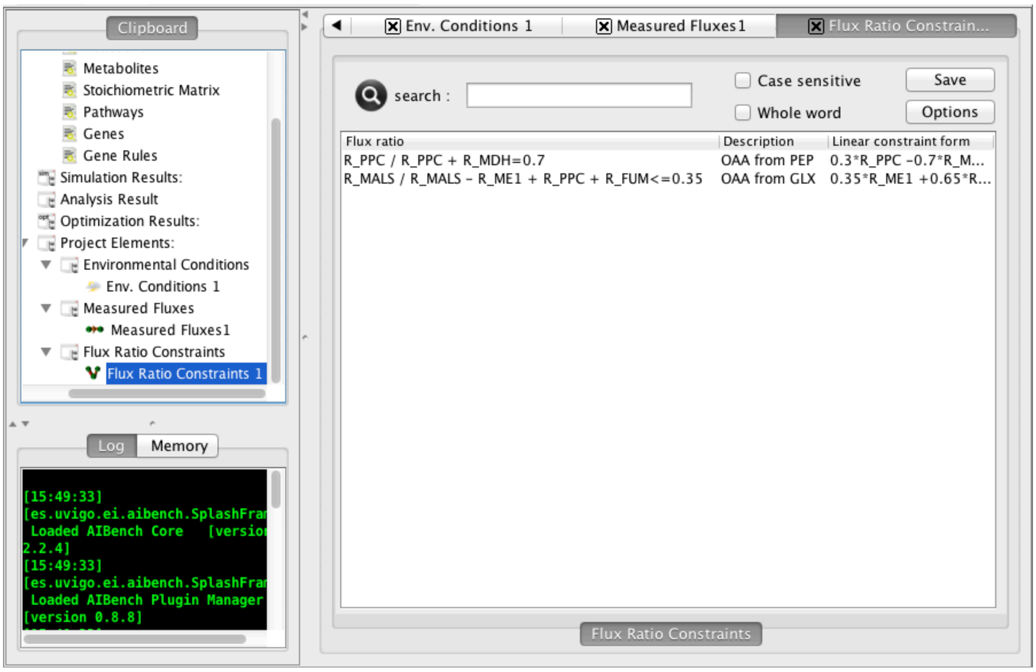

Figure 20: Created flux ratios - data type view

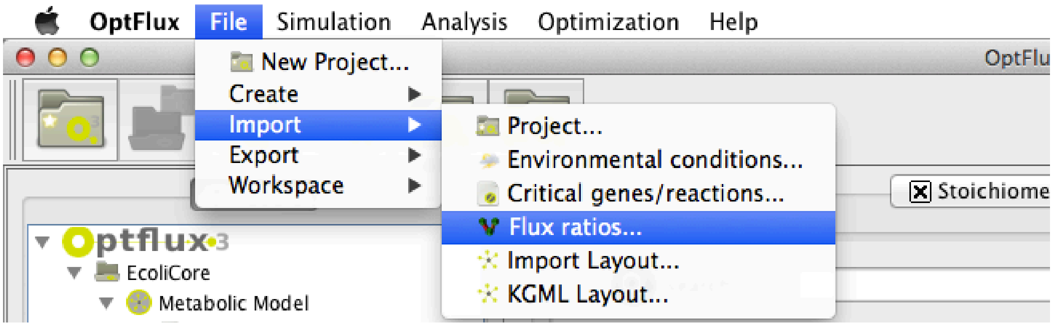

Figure 21: Import flux ratios operation - menu option

are in a MathML-formatted file (please download it in `http://www.optflux.org/cbfa/scRatiosMathML.xml`). The file contains two simple ratios that express the following two flux equalities:

$$\frac{IDP1}{MDH1} = 2 \Leftrightarrow IDP1 - 2\,MDH1 = 0$$

$$\frac{FUM1}{ACO1} = 0.5 \Leftrightarrow FUM1 \ - 0.5 \ ACO1 \ = \ 0$$

If you want to use some flux ratios that have been previously created and extend them with new flux ratios, you must select the desired data type and the existing flux ratios will be added to the "Flux Ratio Constraints" list. However, do not override the data type, if the objective is to create a new instance with the previous ratios and the new ones.

In this example we are not going to extend some existing ratios, but will create a new data type with the ratios loaded from a file only. To select the file, click on the *MathML file* button (Figure 22) and search for the file (in this case, the one you have downloaded).

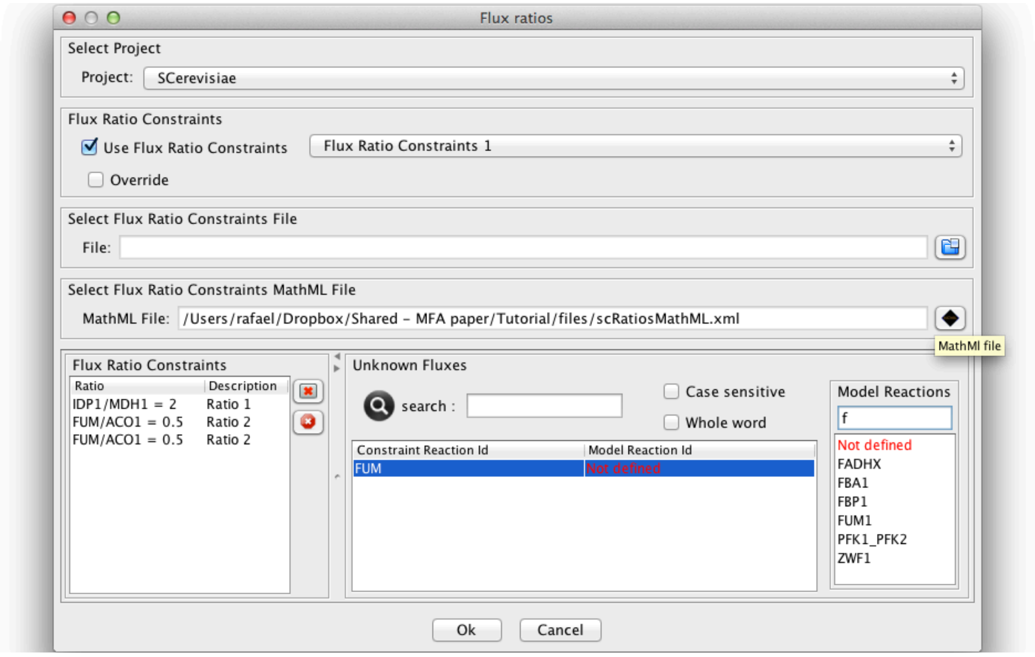

Figure 22: Import flux ratios - operation dialogue

The ratios of the file (*Ratio1* and *Ratio2*) are loaded and added to the "Flux Ratio Constraints" list. As you can see, there is a reaction id in the loaded flux ratios that does not match any reaction in the model. In such cases, the reactions that do not have a matched id are listed on the "Unknown Fluxes"

panel. To associate a proper reaction id to the unknown flux, click on the corresponding row and a panel will be displayed with the model reactions, where you can select the reaction that corresponds to the unknown flux. In this case select the *FUM1* id and click the *Ok* button.

This will create an object containing the four ratios that will be added to the clipboard with the name "Flux Ratio Constraints 2" (Figure 23). If you select it, you will be able to visualize the four ratios and see that the reaction id of the unknown flux is updated in the linear form of the corresponding ratio.

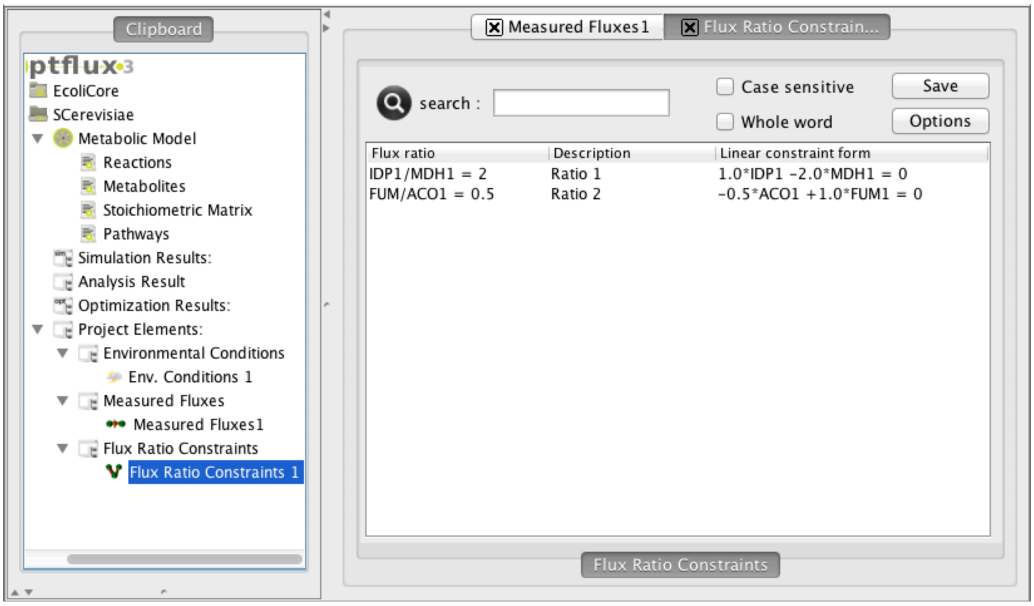

Figure 23: Imported flux ratios - data type view

7 Performing flux analysis with underdetermined systems

We have defined some data types that could express experimental data and are now available to be used to constrain the flux analysis methods in the

simulation operations that we will now address. Here, we will show how to run these methods but we have created a thorough description of their algorithms and formulations in a separate document available in the web site. All the implemented methods follow the same scheme, thus we have decided to encompass all the methods in the same interface, except for the knockout simulations.

To run an flux analysis method, you can select the option **Flux Analysis** on the *Simulation / Flux Analysis* menu as shown in Figure 24.

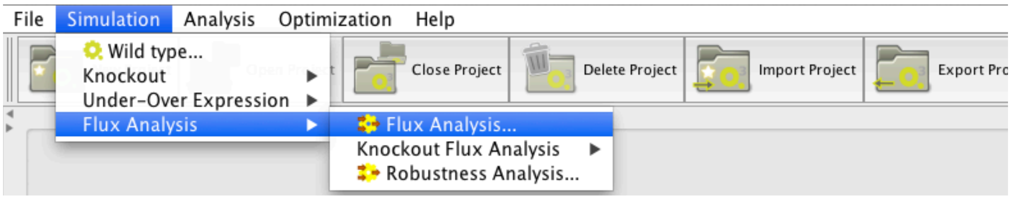

Figure 24: Flux analysi menu option

When this option is selected, the dialogue shown in Figure 25 is displayed. In the following subsections, you will be prompted with this interface so it is reasonable to explain how its panels are organized. On panel **A** it is possible to select the general inputs of the flux analysis methods. From these inputs, the degrees of freedom are calculated and the type of system is assessed. A summary of the relevant statistics leading to the decision are shown in the panel **B**.

Depending on the type of system, different approaches can be selected to perform the flux analysis process. Thus, the approaches that are shown on the box **C** will change if the type of the system is modified when the inputs are updated. The area on box **D** is filled with the options that configure the flux analysis method with respect to the selected approach.

In the following subsections, you should select to use the “Measured Fluxes1” object on the “Select Measured Fluxes” panel and the “Flux Ratio Constraints 1” object on the “Select Flux Ratio Constraints” panel.

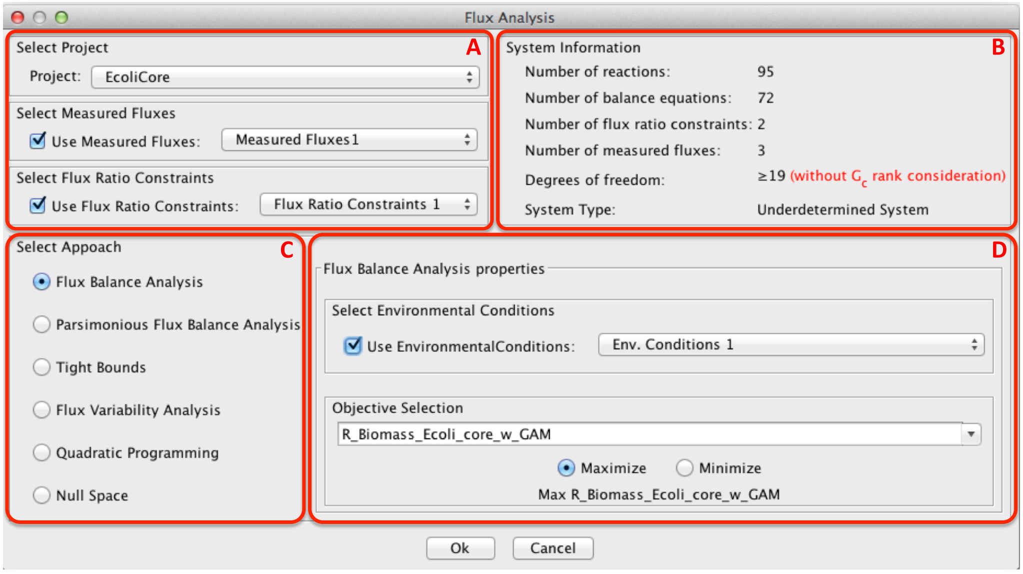

Figure 25: flux analysis operation general view

7.1 Flux balance analysis approach

After selecting the measured fluxes and the flux ratios, you can check, in the "System Information" panel, that the current system is undetermined. Therefore, one option is to perform the Flux Balance Analysis (FBA) based flux analysis method. To do that, select the "Flux Balance Analysis" approach and in the panel D you will find the FBA properties to be configured (Figure 26).

As it is shown, it is possible to select environmental conditions and also to configure the objective function of the method. In this example, select the environmental conditions that you have created before ("Env. Conditions 1"), select the biomass flux (*R_Biomass_Ecoli_core_w_GAM*) in the searchable text field and set it to be a maximization problem.

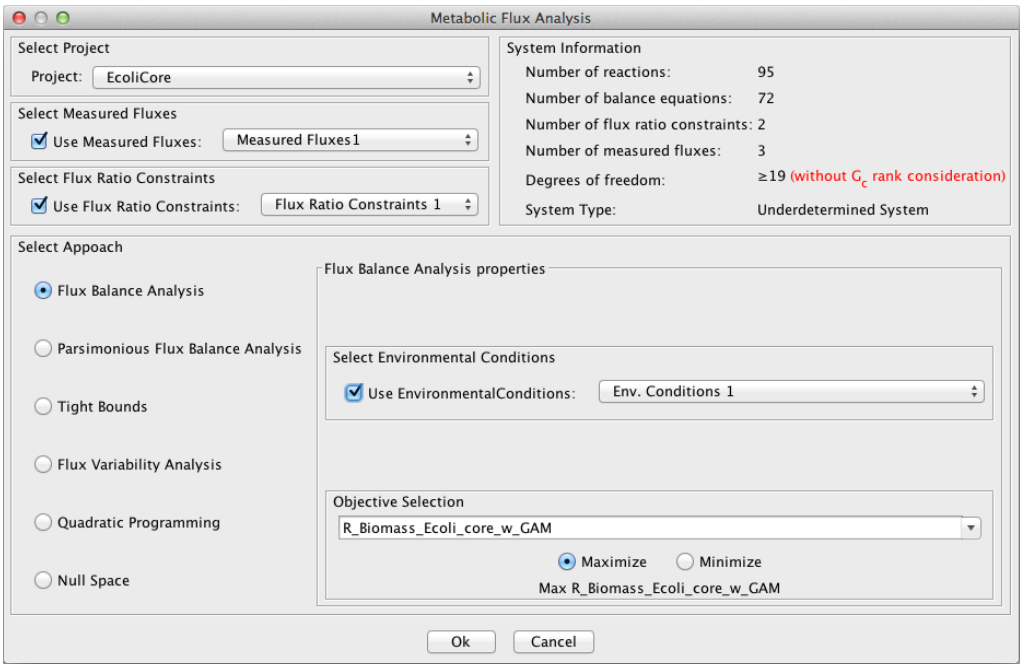

Figure 26: FBA-based flux analysis - operation dialogue

At this point, you have configured all the settings to run the FBA-based flux analysis method. Click on the *Ok* button to execute the operation and a new object, named "CBFA FBA Simulation 1", is added to the clipboard, containing the result of the simulation, under the "CBFA-FBA Simulations" list within the "Simulation results" branch. If you select the object, you will see a set of tabs on the bottom of the visualization area, each containing distinct information about the performed simulation (Figure 27).

On the "CBFA Process Summary" view, you can check the main results of the performed simulation, such as the used flux bounds and ratio constraints. The former is presented as a table where there is a column for the bounds of the fluxes and another with its source. Note that, for instance, the lower bound of the flux *R_FUM* has been updated to zero, since it has been set to be positive in one of the flux ratios. The "Select Source" combo box allows to filter the type of constraints to be visible.

Because the results of the methods have similar information, these results are

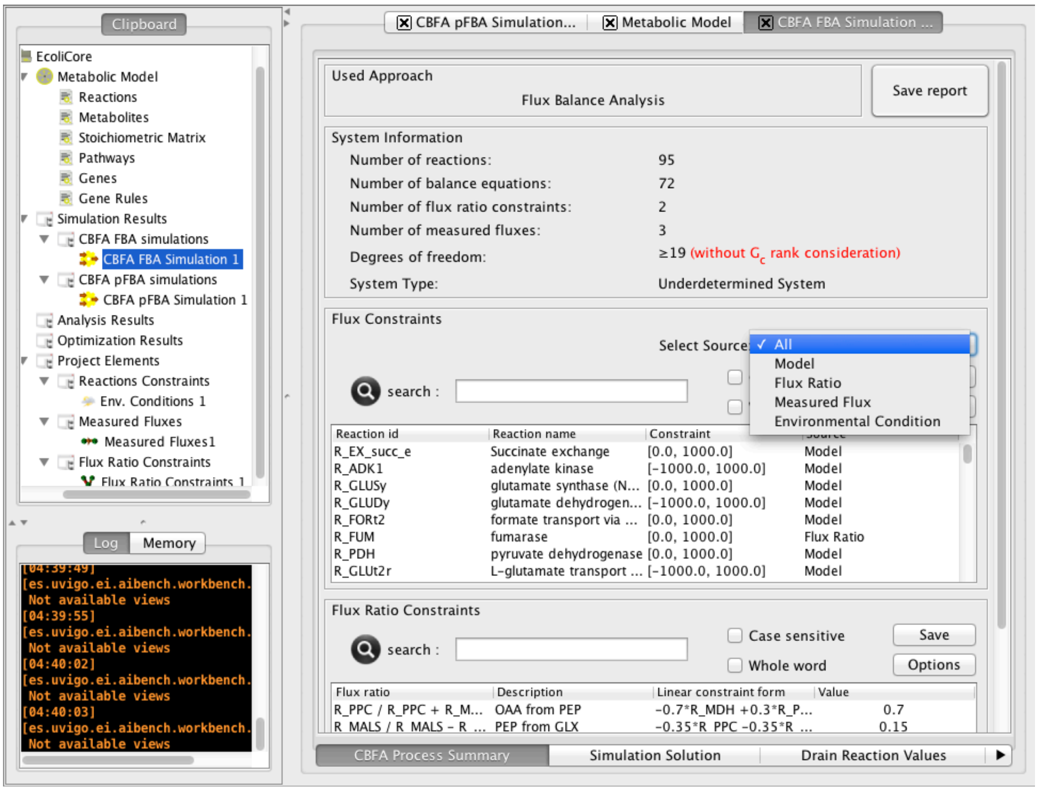

Figure 27: FBA-based flux analysis results - data type view

implemented to respect a set of rules defined by the application programming interface of the software. Therefore, the results of the methods will inherit views that are common to other results. In this tutorial, such views will be shown only once for the first method that uses them (Figure 28), although they are also available for the results of other methods.

The figure is divided in six views that can be selected on the bottom of the visualization area whenever a flux analysis result is added to the clipboard:

- Simulation solution (**A**): here, you can check the simulation configuration, the value of the objective function, the net conversion, etc.
- Drain reaction values and Internal / transport reaction values (**B** and **D** respectively): allow to check the flux values returned by the simulation

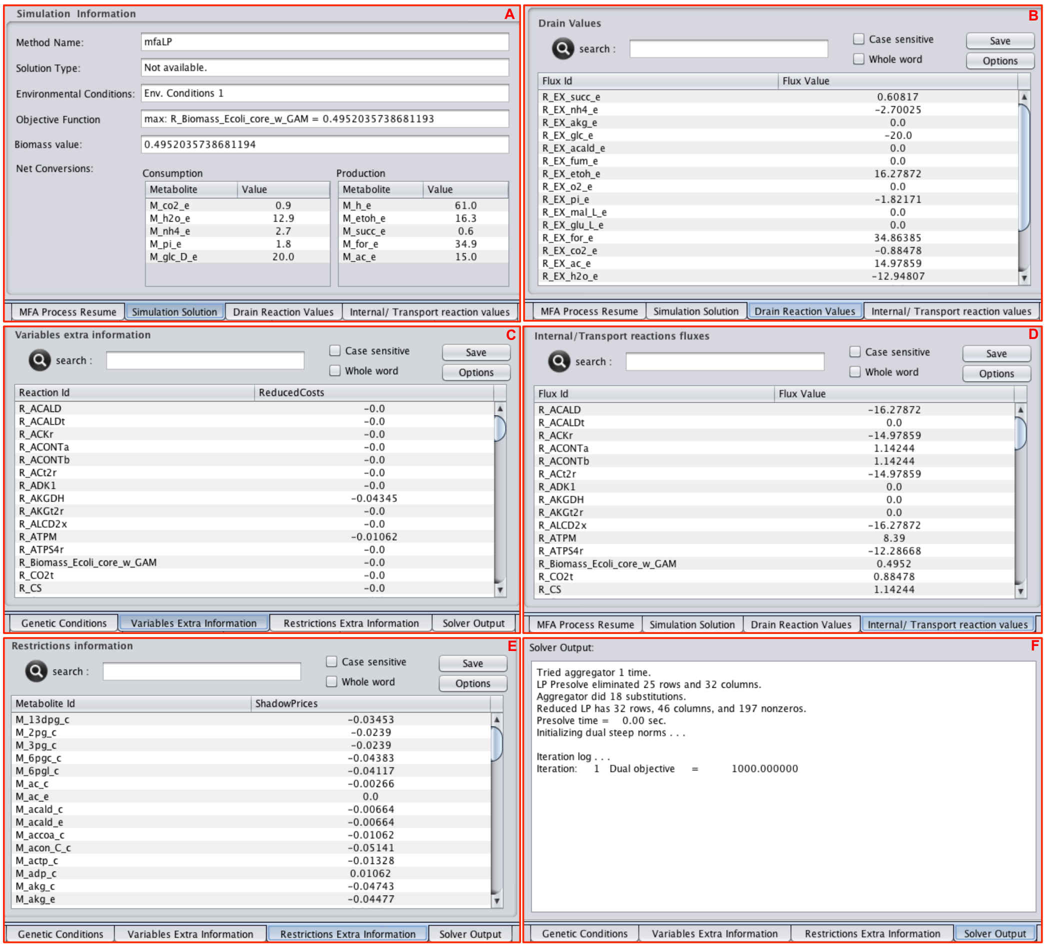

Figure 28: flux analysis results - general views

for all the fluxes (split into drains and internal reactions, respectively); these values can be easily exported in a table format (e.g. CSV). The screenshot shows part of the flux values for internal reactions.

- Restriction and variables extra information (**C** and **E** respectively): allows to check complementary information (if available) returned by the simulation method (can change from method to method).
- Solver output (**F**): textual output of the solver used in the underlying optimization (e.g. linear programming solver).

7.2 Parsimonious Flux balance analysis approach

The Parsimonious enzyme usage Flux Balance Analysis (pFBA) (a variant of FBA) searches for a solution that optimizes the growth rate using the previous FBA-based method and, afterwards, minimizes the sum of the fluxes of all reactions in the stoichiometric model. You can choose to execute an flux analysis method that follows this approach by selecting the option **Flux Analysis** on the *Simulation / Flux Analysis* menu as shown in Figure 24.

Select the “Measured Fluxes1” and the “Flux Ratio Constraints 1” data types, and select the “Parsimonious Flux Balance Analysis” approach. As it is shown in Figure 29, the properties to configure this method are the same as those for the FBA-based method. Thus, select the “Env. Conditions 1” object as environmental conditions and the objective function, which in this case is the maximization of the biomass ($R\_Biomass\_Ecoli\_core\_w\_GAM$).

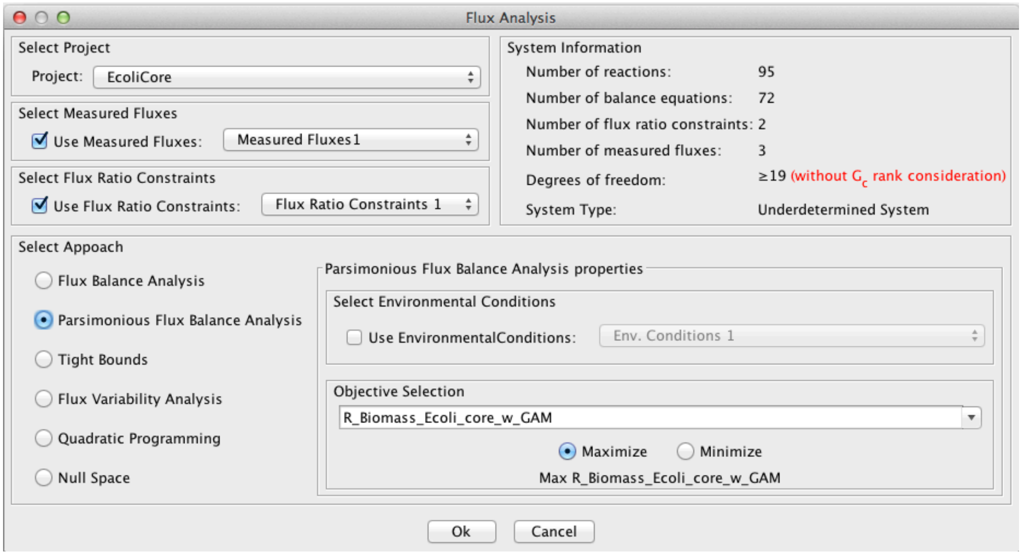

Figure 29: pFBA-based flux analysis - operation dialogue

Click on the *Ok* button and check the result of the operation by selecting the resulting data type “CBFA pFBA Simulation 1” that was added to the clipboard under the “CBFA pFBA simulations” list (Figure 30), that will

hold all the pFBA-based flux analysis solutions. If you select the tabs on the visualization area, you will be able to check that the available views are the same of the previous method. Note that on the "Simulation Solution" view, the value of the objective function is the sum of all fluxes in the generated solution.

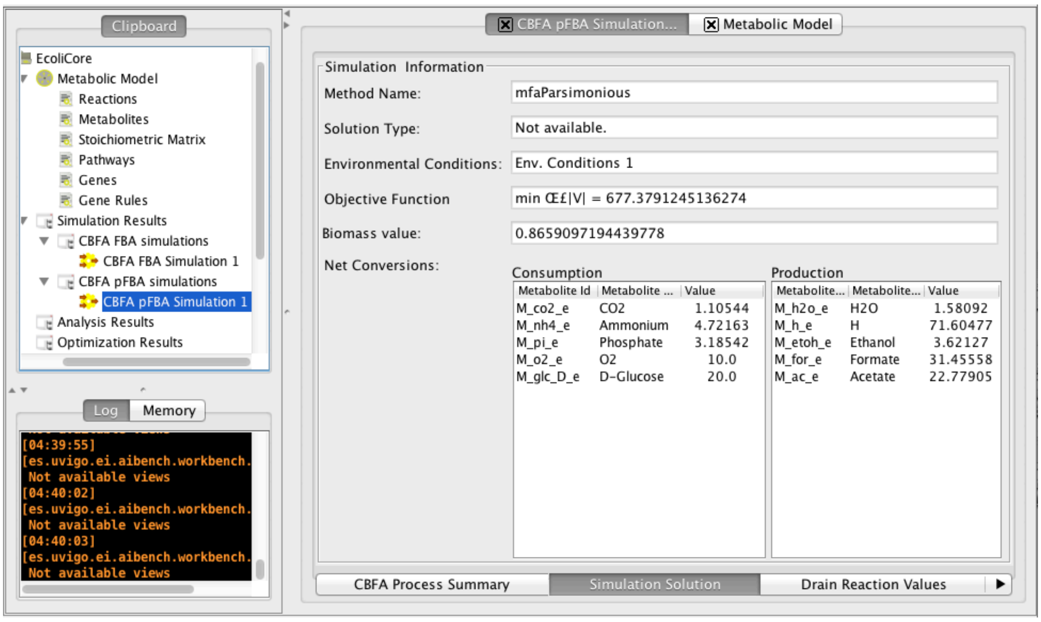

Figure 30: pFBA-based flux analysis results - data type view

7.3 Tight bounds approach

This method is used to compute the lower and upper limits for all non free fluxes of the system. Therefore, unlike the previous method, it does not return a flux distribution that satisfies a given objective function but rather ranges of flux values for each reaction.

To run this operation, first select the **Flux Analysis** operation (Figure 24). The flux analysis operation dialogue will appear and you should select the measured fluxes and flux ratios that you have created. Because this method calculates the minimum and maximum values for all the fluxes that have

not been measured neither selected for knockouts (as you will see later on this document), without any specific objective function to be optimized, the properties that are shown when you select the "Tight Bounds" approach are only the environmental conditions (Figure 31). Thus, simply select the "Env. Conditions 1" object and click on the *Ok* button.

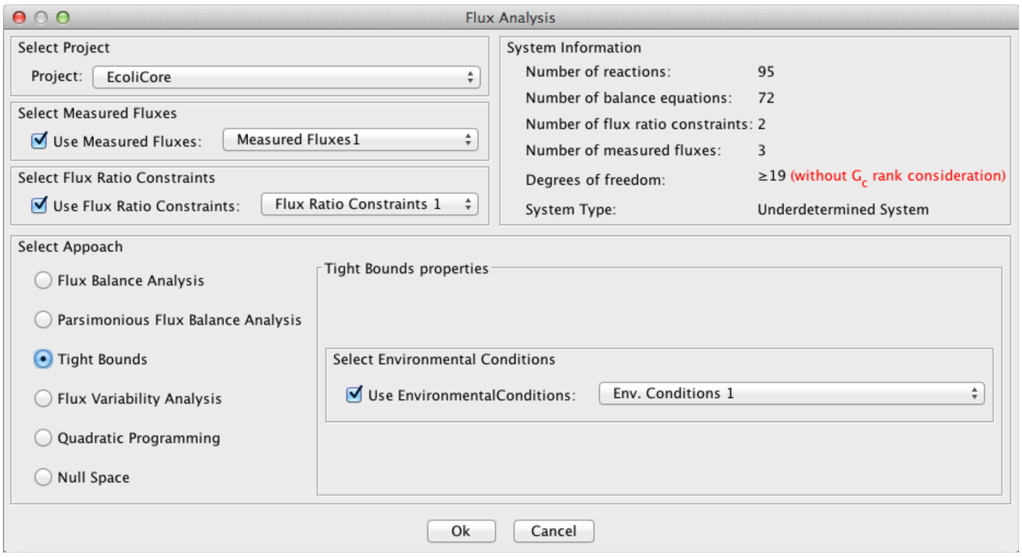

Figure 31: Tight bounds flux analysis - operation dialogue

This operation might take a bit longer than the others because it has to perform two optimization processes (minimization and maximization) for every non free flux. When it is finished, the resulting object is added to the clipboard, in this case named "CBFA TightBounds Simulation 1 ", placed under "CBFA Tight Bounds Simulations" list. This result has an additional tab, named "CBFA Tight Bounds" that does not appear on the preceding methods. This tab enables to access the lower and upper bounds that is possible to calculate for the non free fluxes (Figure 32), given the environmental and experimental conditions that you have chosen.

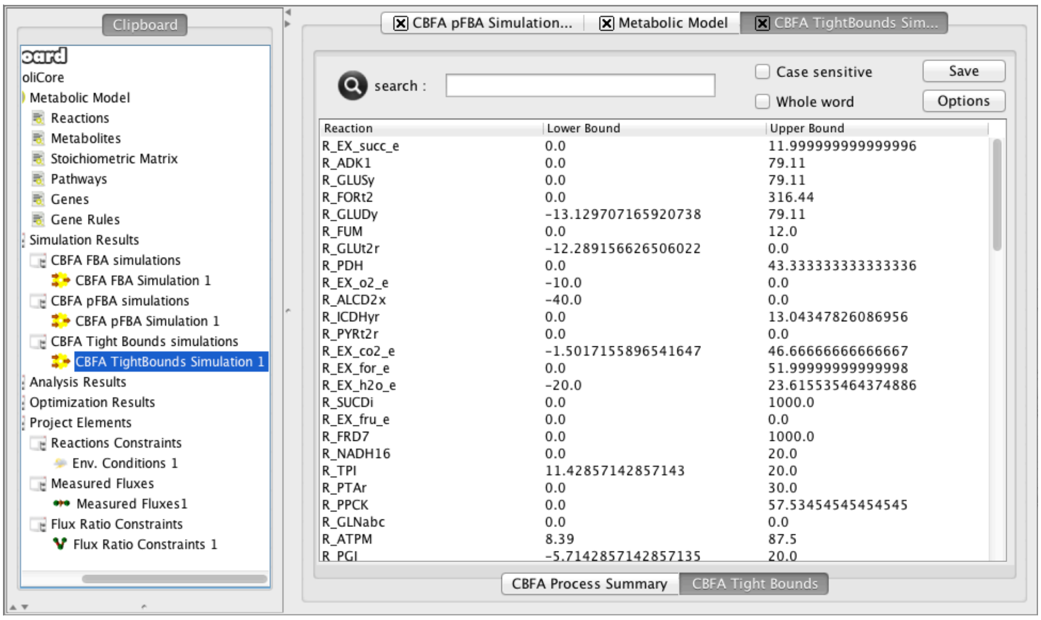

Figure 32: Tight bounds flux analysis results - data type view

7.4 Flux Variability Analysis approach

You can use the Flux Variability Analysis (FVA)-based flux analysis method to determine how fluxes can change when alternative solutions are obtained by a Linear Programming (LP) problem, that satisfies a set of configured constraints, and allows for the same optimal objective function value. To do that, select the flux analysis operation as described above.

After selecting the “Measured Fluxes1” and the “Flux Ratio Constraints 1” objects, you can see that the system is under-determined and you can select the “CBFA Tight Bounds” approach. In this example, we are going to calculate the maximum and minimum possible values of the whole set of non free fluxes of the model, given a defined level (90%) of the biomass flux (relative to the wild type) (Figure 33). However, you can define a different objective function and a different flux to be fixed in the desired level. Once again, select “Env. Conditions 1” to set up your environmental conditions.

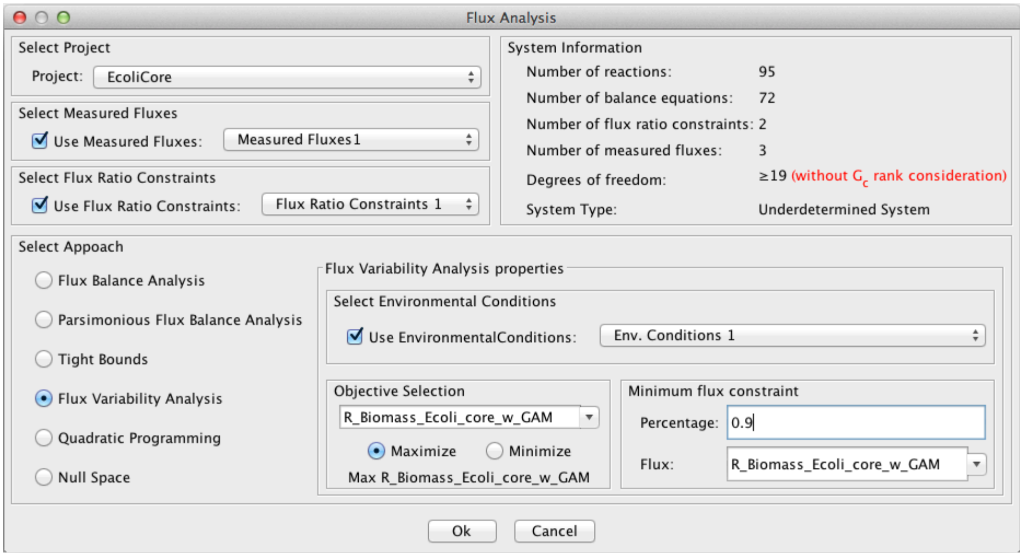

Figure 33: FVA-based flux analysis - operation dialogue

When the operation ends, the "CBFA FVA Simulation 1" data type is created and returned to the clipboard. A view named "CBFA FVA Bounds", similar to the one described in the tight bounds method, is available and you can visualize the bounds of the fluxes in the model, the value of the objective function and the value of the flux that was selected to be fixed.

7.5 Quadratic Programming approach

The QP-based flux analysis method formulates an optimization problem that minimizes the sum of the squares of the differences between the measured and the calculated fluxes. Thus, to use this approach, when you choose to run the flux analysis operation (see Figure 24) and the dialogue of the operation is displayed, you have to select an object with flux measurements. Otherwise, the "Quadratic Programming" approach will not be visible on the dialogue.

To illustrate how to execute this operation we are going to use the same metabolic flux ratios we have been using ("Flux Ratio Constraints 1") but a different set of flux measurements. Please import the measured fluxes

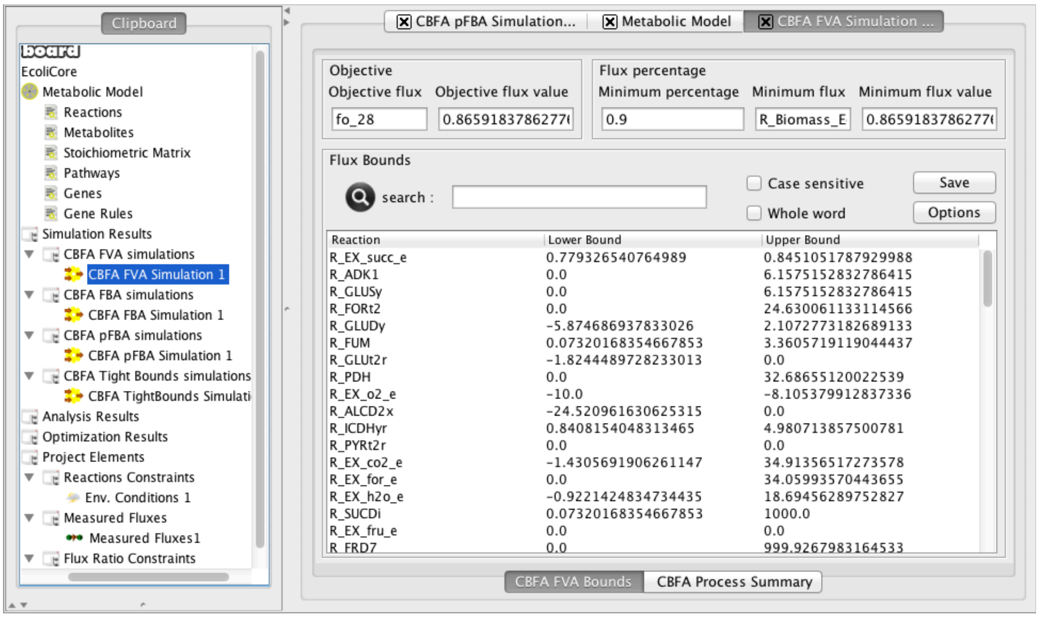

Figure 34: FVA-based flux analysis results - data type view

from the file “ecMeasurements.csv” available on `http://www.optflux.org/cbfa/ecMeasurements.csv` (refer to section 5.2 to check how to import flux measurements), and select the created data type to enable the QP option on the “Select Approach” panel. Also, select the “Flux Ratio Constraints 1” as metabolic flux ratios. As you can see in the “System information” panel, the system is still underdetermined, however this approach can also be performed to the other types of system. When you select the “Quadratic Programming” approach, you only have to select the environmental conditions, since the objective function is configured automatically from the measurements that have been selected (Figure 35).

When the simulation is finished, the result with the id “CBFA QP Simulation 1” is sent to the clipboard and you can navigate through the different views that are available (Figure 36). Here you can notice that the accessible views are exactly the same of the ones for the FBA and pFBA based methods.

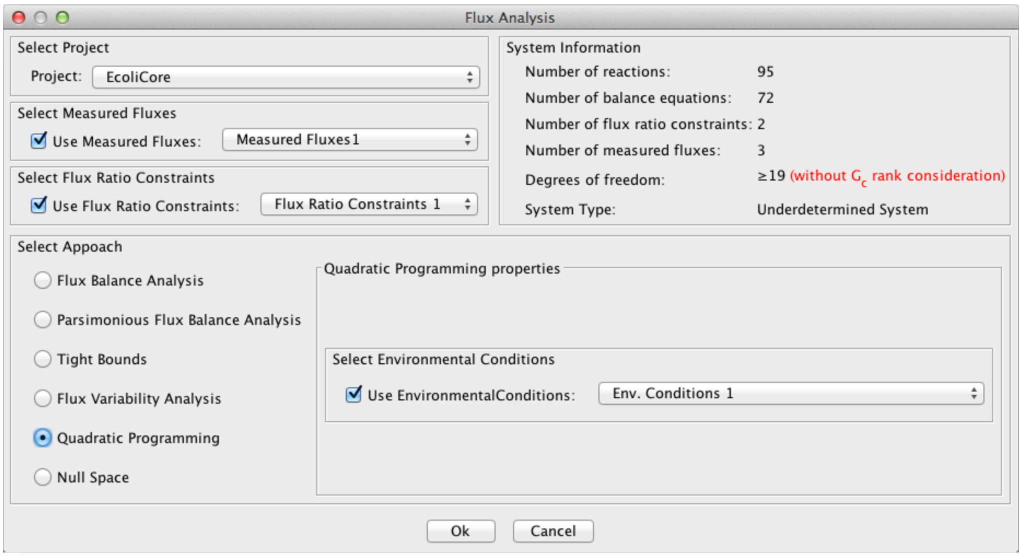

Figure 35: QP-based flux analysis - operation dialogue

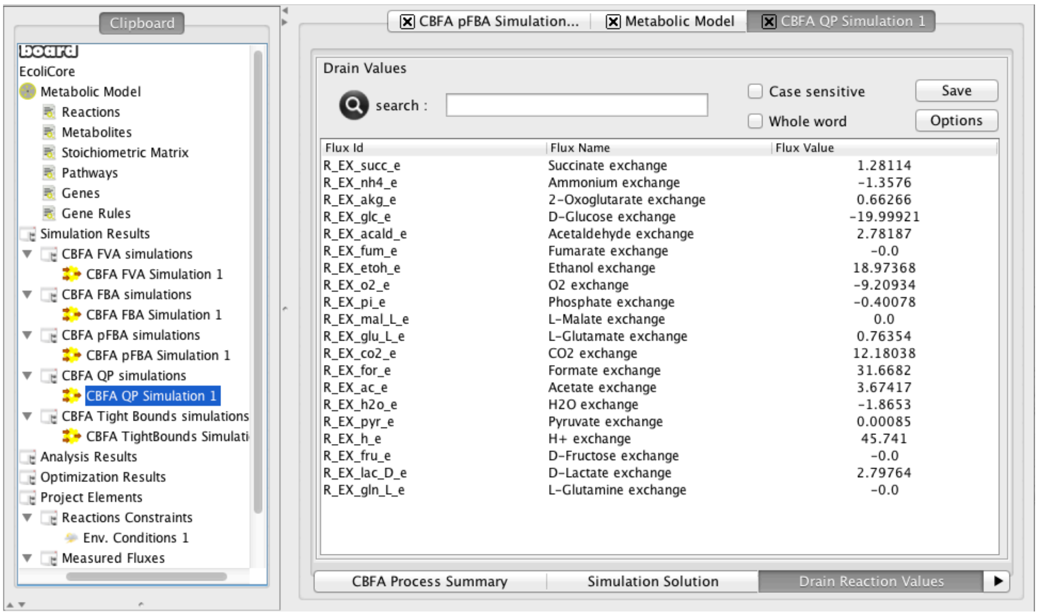

Figure 36: QP-based flux analysis results - data type view

8 Robustness analysis

In order to investigate how an objective function can change, if the flux values of the optimal solution changes, a robustness analysis method is available. The analysis can be performed using the same set of inputs as described above.

The robustness analysis operation is made available through the option **Robustness Analysis** on the *Simulation / Flux Analysis / Robustness Analysis* menu as shown in Figure 37.

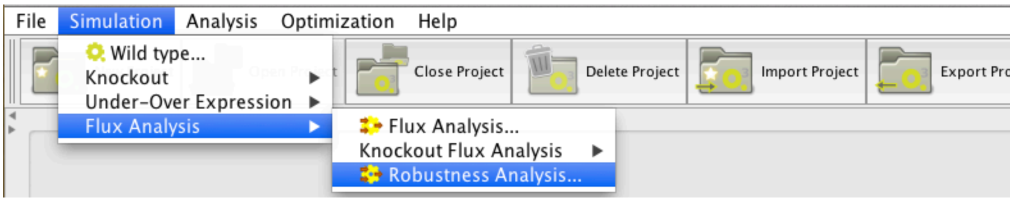

Figure 37: Robustness analysis menu option

After selecting this option, the corresponding dialogue is opened (Figure 38). As it is shown, a loaded model must be chosen and, if exists, environmental conditions, flux measurements and environmental conditions can be selected.

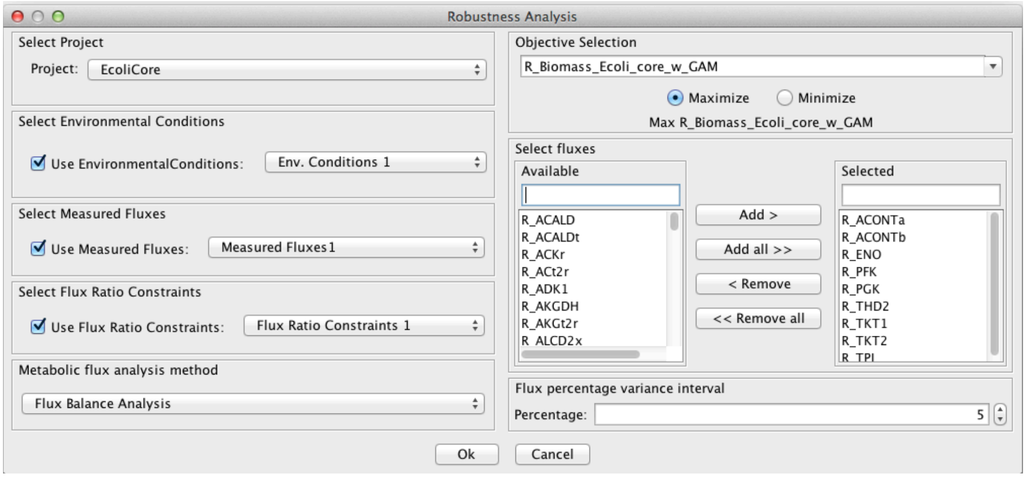

Figure 38: Robustness analysis - operation dialogue

Here, the user can select either the FBA-based method, or the pFBA one. In this example, select the “Flux Balance Analysis” method, and select the “Env. Conditions 1”, “Measured Fluxes1” and “Flux Ratio Constraints 1” datatypes to constrain the simulations. Also, select the biomass flux (*R_Biomass_Ecoli_core_w_GAM*) for the objective function and set it to be a maximization problem.

Now, its time to select the fluxes for which the robustness analysis will be done (the right box in the “Select fluxes” panel indicates the fluxes that are selected’). The following fluxes have been selected in this example:

- *R_ACONTa*
- *R_ACONTb*
- *R_ENO*
- *R_PFK*
- *R_PGK*
- *R_THD2*
- *R_TKT1*
- *R_TKT2*
- *R_TPI*

When all the configurations are set, and the ‘OK” button is pressed, the operation will be executed. If the number of selected fluxes is too high, this may take a while. When all the simulations of the analysis are accomplished, a datatype with the name “CBFA Robustness Analysis 1” is added to the clipboard. On the view area you can select the “Robustness Analysis” tab to check the plots of each flux analysis (Figure 39). Here, the user can select the fluxes to be shown in the main plot, change the color of each flux and visualize them separately, by clicking on their right-hand side button.

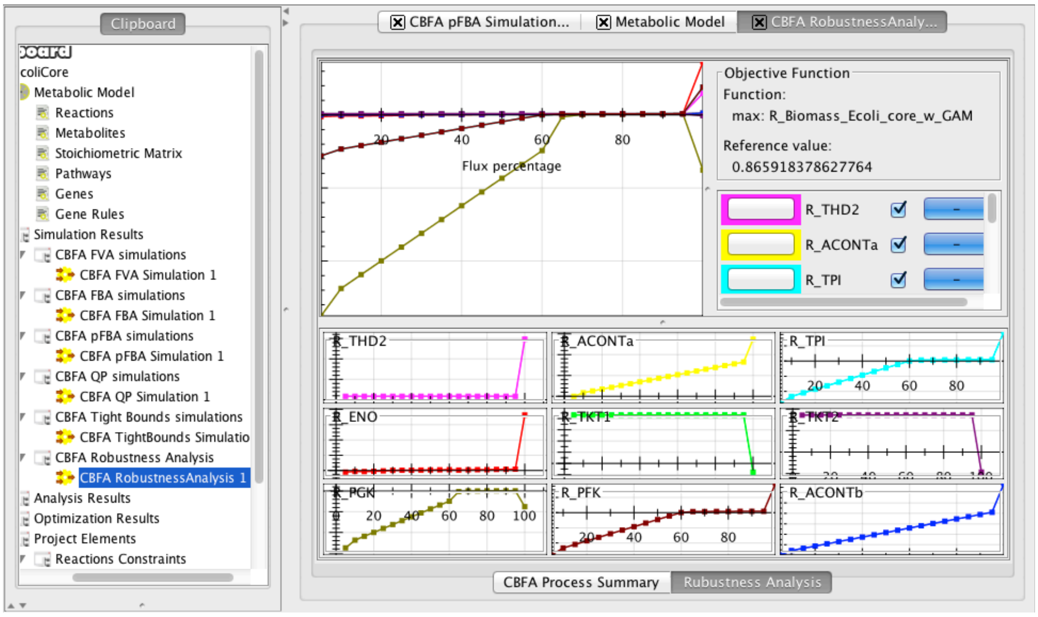

Figure 39: Robustness analysis - data type view

9 Performing flux analysis for mutant simulation

9.1 Mutant flux analysis - reaction deletions

One other alternative is to perform flux analysis for mutant strains. We will start by checking how to simulate the case where certain reactions are removed from the model. You can access the **Reaction Knockout Flux Analysis** option under the *Simulation/ Flux Analysis/ Knockout Flux Analysis* menu (Figure 40) or right click on the Metabolic Model icon on the clipboard.

When launching the operation, a panel similar to the one from the flux analysis operation appears as shown in Figure 41. In this panel, it is possible to select the inputs to configure the system as in the normal flux analysis operations, and also a panel named “Reactions Knockouts” to select the

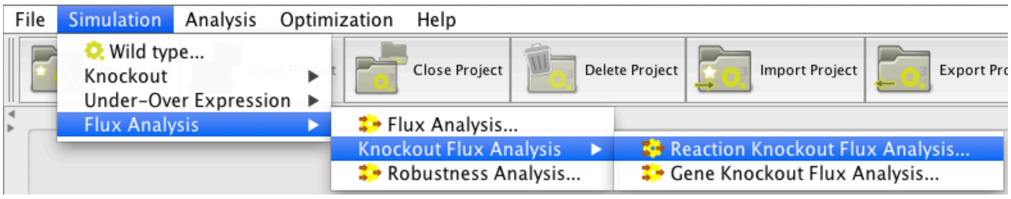

Figure 40: Reaction knockout flux analysis operation - menu option

reactions in the model to be removed. In this example, select as measured fluxes, the data type "Measured Fluxes 1", and the "Flux Ratio Constraints 1", as metabolic flux ratios. Additionally, the reactions *R_ACKr* and *R_Act2r* should be included in the knockout list. For selecting the reactions to be deleted, you just need to choose the desired reactions and press the *Add* button to add them to the right box in the knockouts panel.

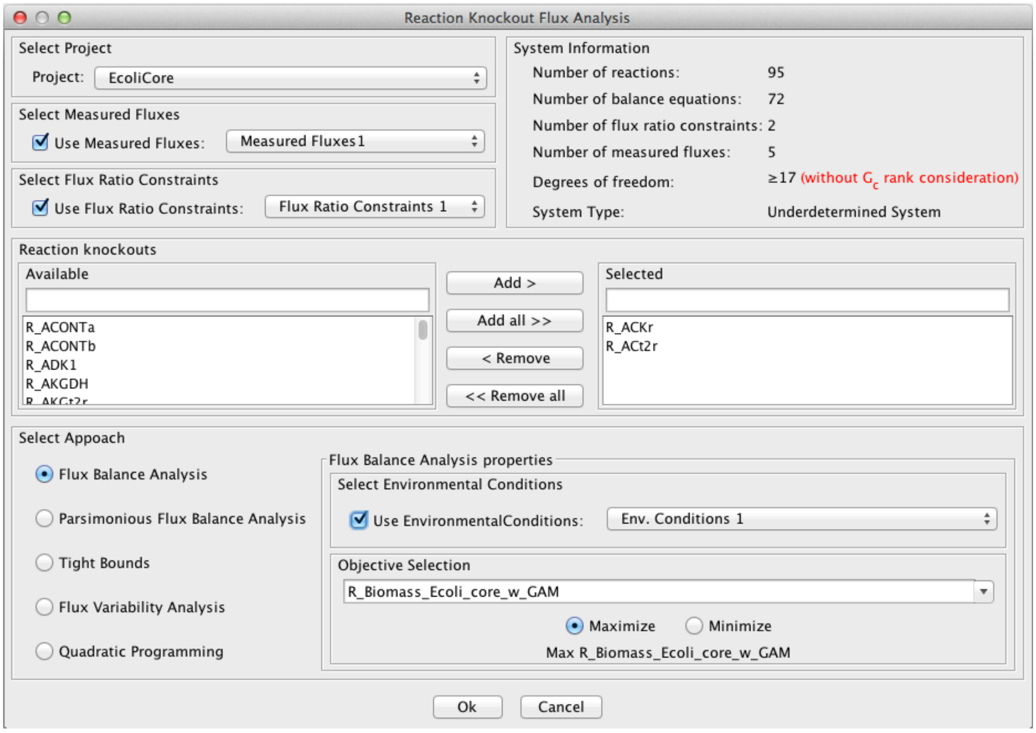

Figure 41: Reaction knockout flux analysis operation - operation dialogue

As it is shown, the flux analysis or mutant strains enables the use of all the approaches previously described for wild type cases. Therefore, when

selecting the knockouts, the available approaches will be listed in the operation panel, and the method to be used can be configured as before. In this case, select the "Flux Balance Analysis" method and on its configuration, select the anaerobic environmental conditions that we have created and set the objective function of the problem to maximize the biomass flux as done previously.

The output of the operation will be added to the clipboard with the name "CBFA FBA Simulation 2" nested to the FBA simulations of the project. In Figure 42 the visualization area of the interface is shown, where a new type of constraint has been inserted in the problem formulation, resulting from the configured knockouts.

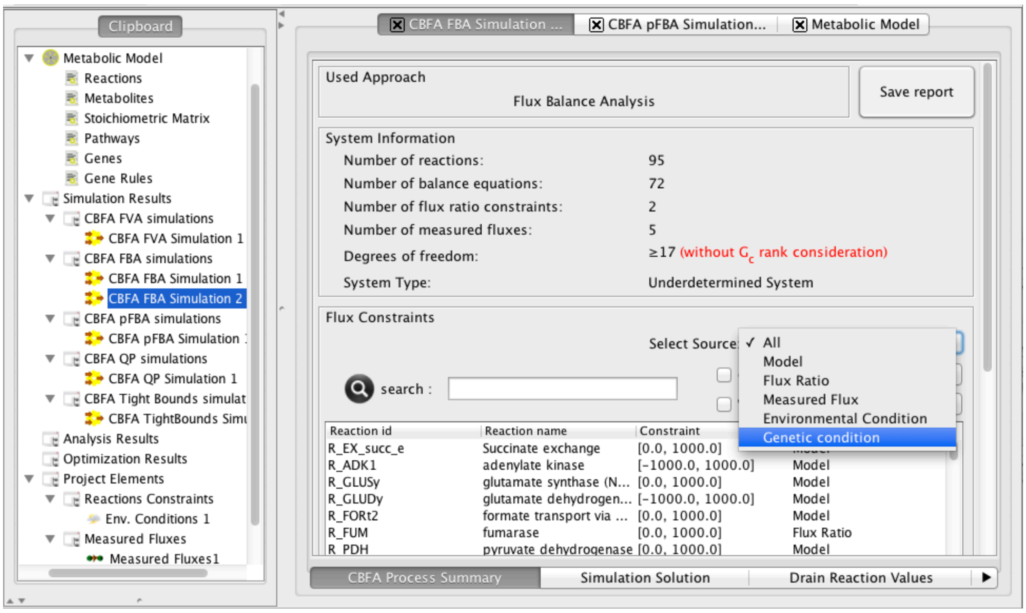

Figure 42: Reaction knockout flux analysis results - data type view

9.2 Mutant flux analysis - gene deletions

If the model contains information on genes and gene-reaction associations, it is possible to perform flux analysis of mutant strains with gene knockouts.

This option is available through the **Gene Knockout Flux Analysis** option under the *Simulation/ Flux Analysis/ Knockout Flux Analysis* menu (Figure 43).

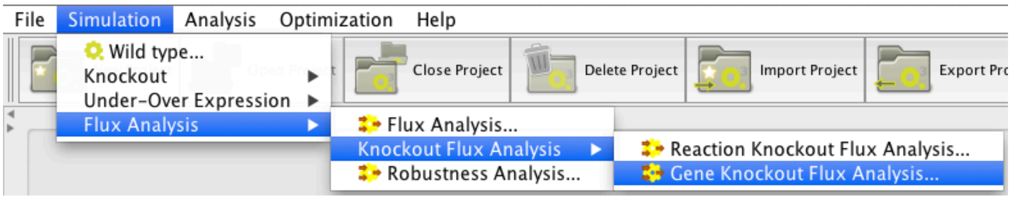

Figure 43: Gene knockout flux analysis operation - menu option

The configuration of this operation is very similar to the one for reactions knockout simulation. Therefore, select the same data types as in the previous section. Indeed, in this case, the only difference relies on how the deleted reactions are configured, since the selection is made through a set of genes to knock out. In the user interface shown in Figure 44, you can add/remove genes, using the arrows buttons to the knockout list (the list of genes to be knocked out, in the "Selected" panel). On the rightmost panel ("Inactive Reactions"), the list of reactions that will be deleted from the model, as a result of the selected gene knockouts, is presented.

After selecting the genes *b0114*, *b0115*, *b0116* and *b3403* the following reactions will be inactivated: *R_PPCK*, *R_PDH* and *R_AKGDH*. Although all the approaches for performing flux analysis can be used in this operation, in this example we only illustrate how to perform a pFBA-based flux analysis. After selecting the environmental conditions and defining the objective function, execute the operation, and the result of the mutant simulation will be added to the clipboard with the name "CBFA pFBA Simulation 2" (Figure 43). This data type has the same views of the previous solutions in the other operations, and the figure shows the set of genes knockouts that have been selected and the resulting inactive reactions.

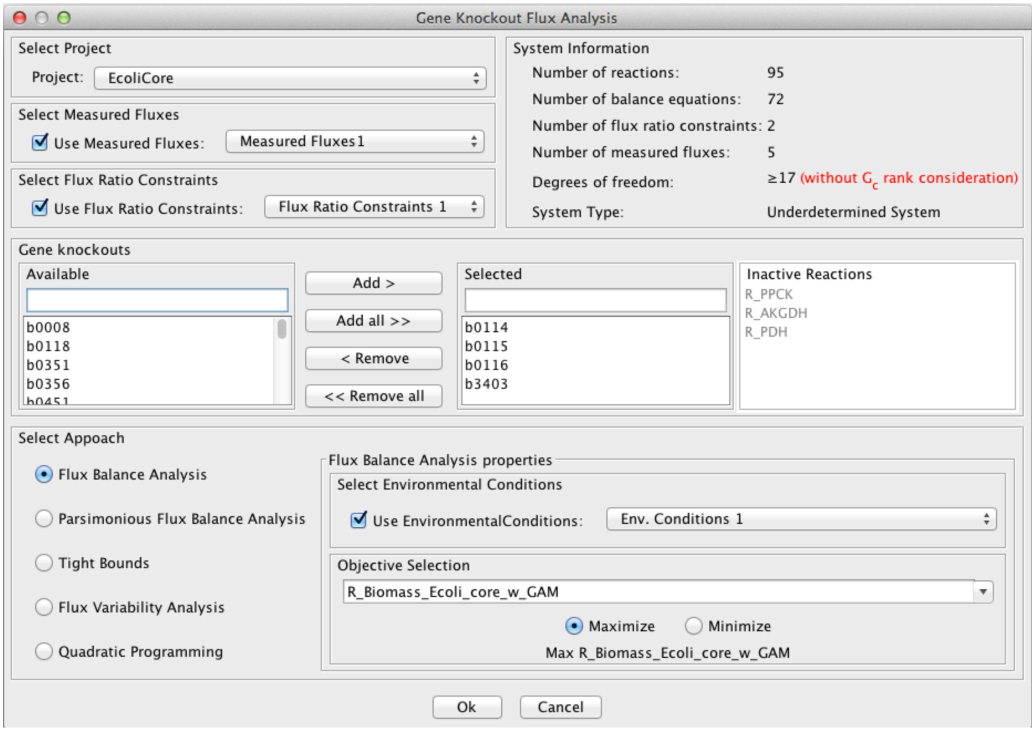

Figure 44: Gene knockout flux analysis operation - operation dialogue

10 Solving the systems algebraically

In the previous sections we have used approaches based on simulation methods to estimate the unknown fluxes from the input constraints. Here, we are going to illustrate how to calculate fluxes in an algebraically manner. In order to do that, the configured system has to be either determined or overdetermined.

To perform this type of flux analysis, we will use a different metabolic model, since the model that we have been using does not allow to obtain a system that is not underdetermined easily. The reason lies on the fact that the number of flux measurements, knockouts or flux ratio constraints, to be given to the system has to be too high to enable to obtain a full rank matrix, that would allow the calculation of the unknown fluxes. Moreover, even with a sufficient number of measured fluxes and knockouts, some problems frequently

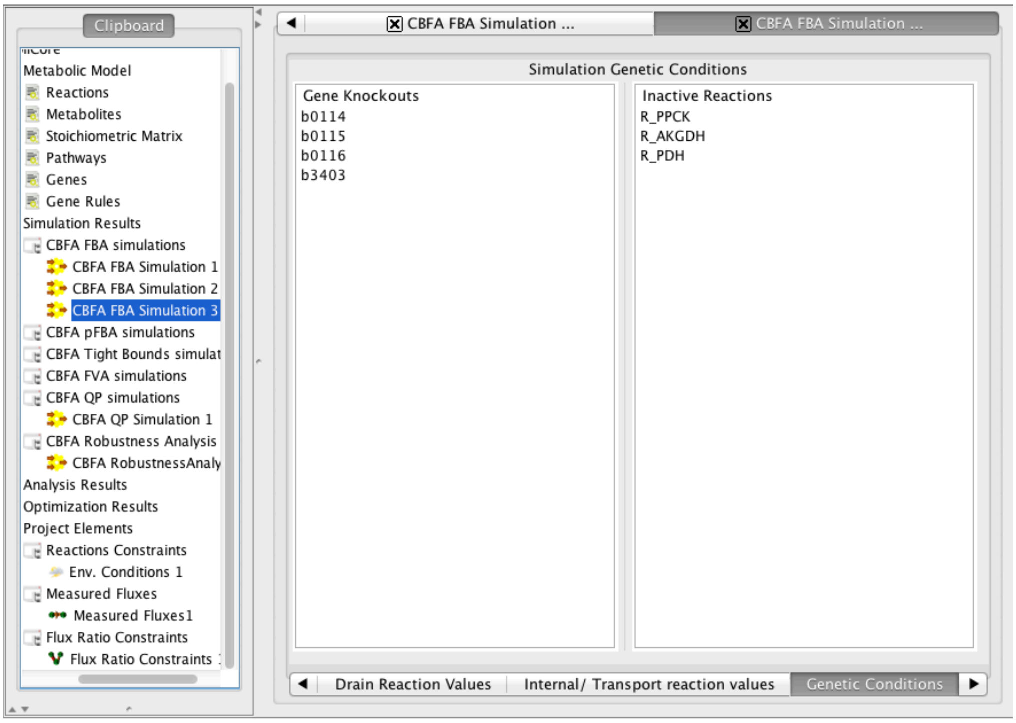

Figure 45: Gene knockout flux analysis results - data type view

arise due to the ill-conditioning of the system. Therefore, to make this tutorial an easy guide to perform the software tasks, we are going to use a simplified model for growth of *Saccharomyces cerevisiae* [Förster et al., 2002]. To use this model, please download the file *gomGG.xml*, available from `http://www.optflux.org/cbfa/gomgg.xml` and create a new project from this model such as it is illustrated on section 3.

Also, enable the environmental conditions to be aerobic, by updating the lower bounds of uptake for glucose and oxygen (*R_EX_GLC_* to -20 and (*R_EX_O2_* to -1000, respectively) as shown on section 4. Make sure that, on the next tasks, the proper project with the last loaded model is chosen. To avoid the selection of previous created projects during the tasks of the following sections, you can close them through the *File / Workspace / close project* menu.

When working with this model, although the difference between the unknowns and equations is far less than with the previous model, it is still necessary to add either some flux measurements, knockouts or flux ratio constraints to make the system (over)determined. Here, we are going to import some flux measurements from the file "scMeasurements.csv" available on `http://www.optflux.org/cbfa/scMeasurements.csv`. To see how to import the measurements please refer to the section 5.2. However, with these flux measurements there are still degrees of freedom. Thus, lets also import some metabolic flux ratios from the MathML-formatted file available on `http://www.optflux.org/cbfa/scRatiosMathML.xml` (please refer to section 6.2 to check how to import flux rations).

Like in the other approaches, to execute the flux analysis operation for this type of systems, choose the same **Flux Analysis** operation from the *Simularion / Flux Analysis* (Figure 24 on page 24).

To enable the available approaches to solve the system, let's select the "Measured Fluxes 1" of the current model project, as the measured fluxes of the system. After that, you will notice, from the "System Information" panel, that the type of the system was updated to overdetermined as shown in Figure 46.

In this example, we are not going to use additional information on the variance of the measured fluxes, and therefore let's select the "Least Squares Fitting" on the "Algebraic Method properties" panel, after selecting the "Algebraic Method" approach and click on the *Ok* button.

When the system is solved, the result is sent to the clipboard with the id "CBFA Algebra Simulation 1", and you can check the values of the internal and external calculated fluxes (Figure 47). Because this method uses some algebraic operations that have some mathematical pre-conditions, to proceed with its operations, if the configured system does not respect the requisites, a warning will be displayed with a text message showing the reason that prevents the system from being solved.

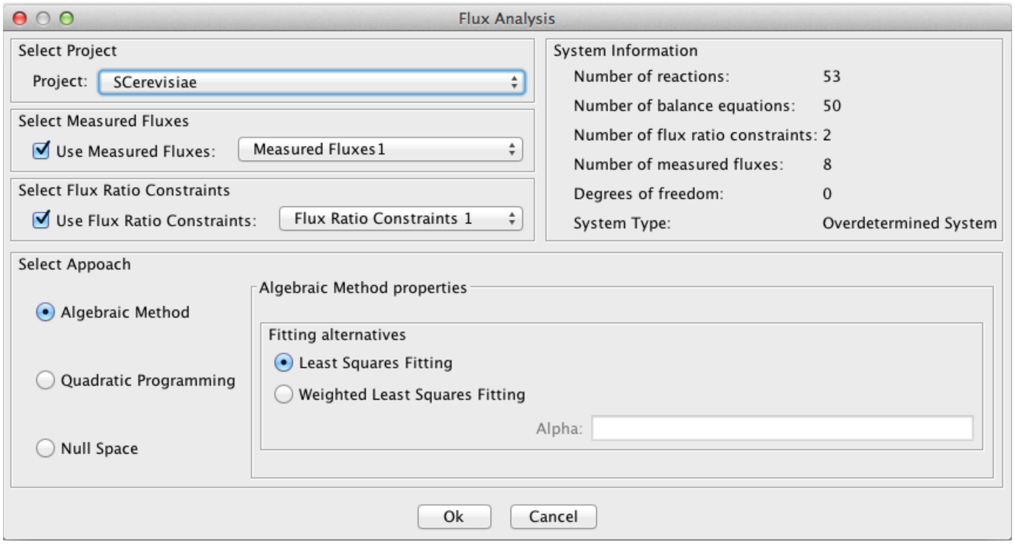

Figure 46: Flux analysis with algebraic methods - operation dialogue

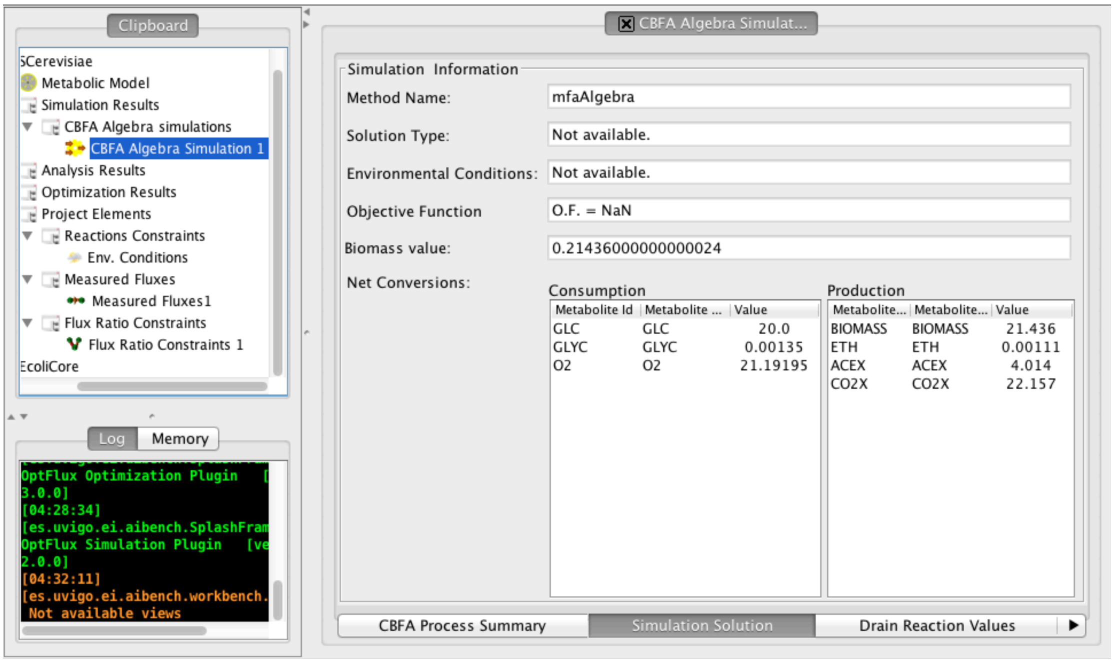

Figure 47: Results of flux analysis with algebraic methods - data type view

11 Performing null space-based flux analysis

This method is based on the null space of the stoichiometric matrix, or the extended stoichiometric matrix, if ratio equations are used. It can be used to solve all the three types of systems, but when the system is underdetermined it does not return a single solution. Instead, it returns what is called of "Principal Solution" and the option to generate alternative solutions from the base vectors that span the null space of the system, based on the measured fluxes (and knockouts) given as input.

Therefore, to illustrate how to deal with the possibility of generating alternative solutions, after selecting the flux analysis operation as in the preceding sections, let's use the same data types of the previous section, but with no flux ratios, so the system will have degrees of freedom, and consequently, be underdetermined (Figure 48).

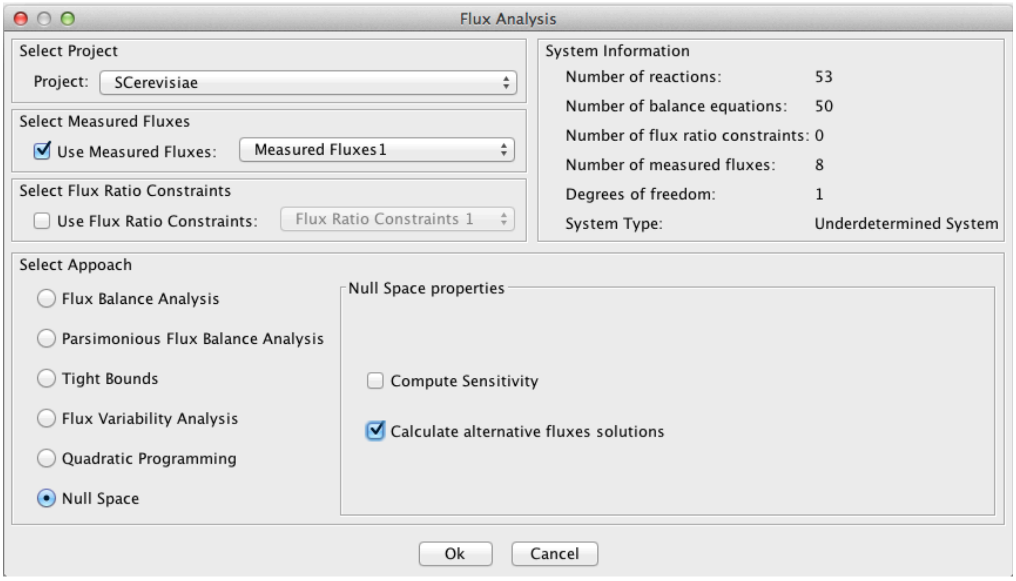

Figure 48: Null space-based flux analysis - operation dialogue

After selecting the *Saccharomyces Cerevisiae* model and the measured fluxes that you have previously imported, you can select the "Null Space" approach and on the properties panel, check the box "Calculate alternative fluxes so-

lution" so the method will compute alternative solutions depending on the rank deficiency of the null space based on the measured fluxes.

When the operation is performed, a new data type "CBFA Nullspace Simulation 1" is added to the clipboard. As you can see in Figure 49, this data type has a nested data type that corresponds to the principal solution obtained by the method. Nevertheless, if the user wants to check the base vectors that were used to generate the principal solution, it is possible to visualize the base vectors and the beta values for the free fluxes on the visualization area, through the "Nullspace" view.

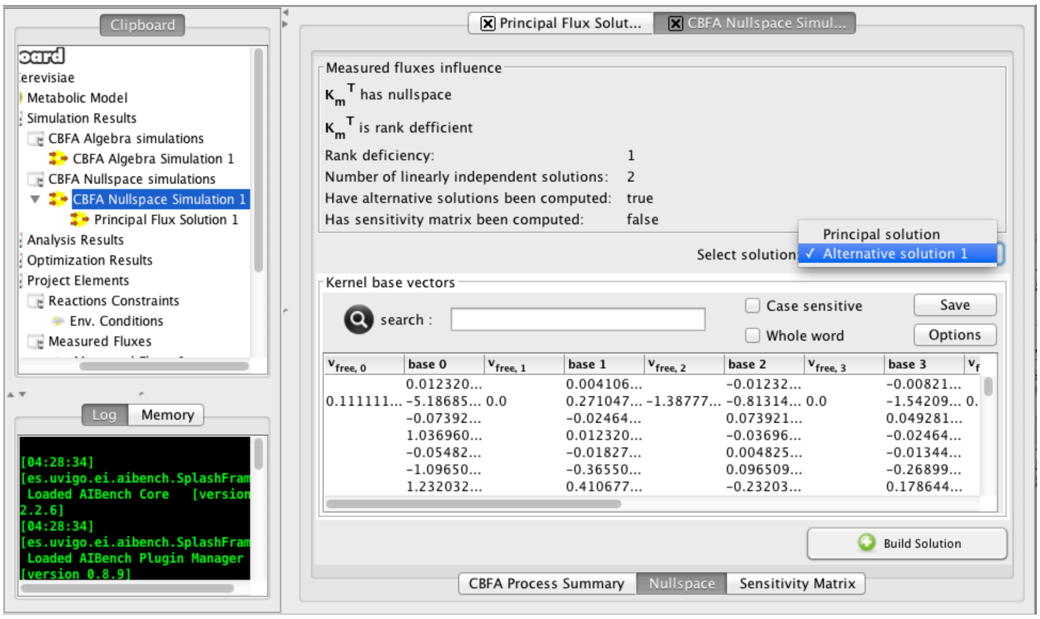

Figure 49: Nullspace-based flux analysis solution - data type view

In this example, there is only one alternative solution, but when the system has many degrees of freedom, several alternative solutions can be generated, hence it is not viable to instantiate all the alternatives to the clipboard. Rather, the software gives you the possibility to check the beta values for the alternative solutions, by selecting the corresponding solution on the "Select solution" combo box, and build a new data type for the solution by clicking on the *Build Solution* button. As it happens with the principal solution, this

data type is nested to the “CBFA Nullspace Simulation 1” data type on the clipboard and the user can select the different nullspace solutions and check the corresponding views like in the results of the other methods.

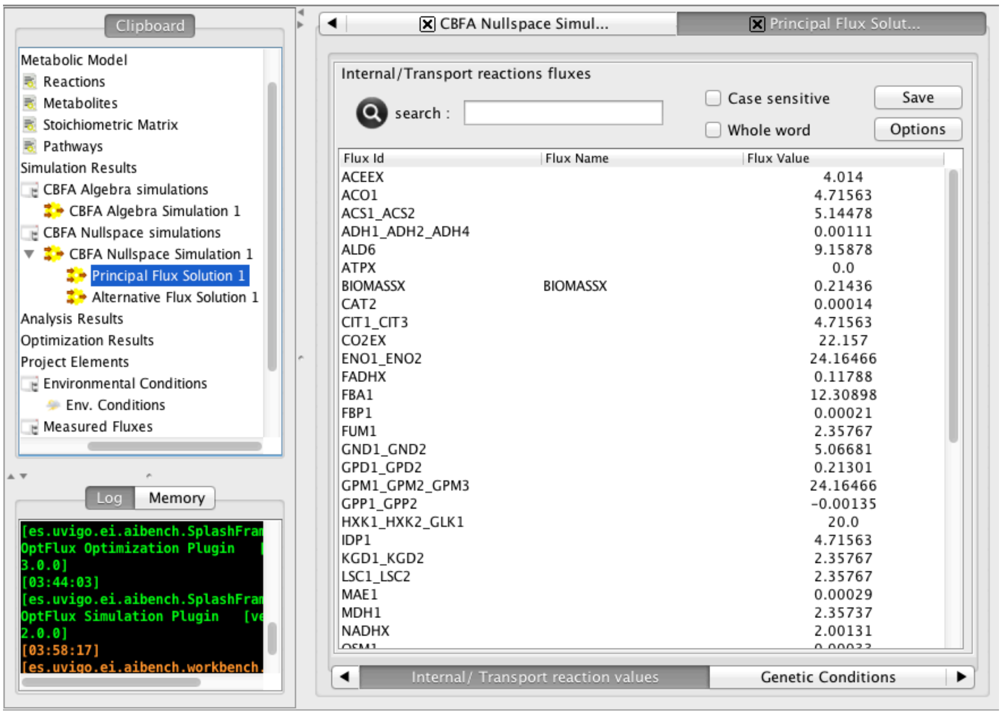

Figure 50: Nullspace-based flux analysis principal solution data type view

12 Visualizing the flux distribution of the flux analysis methods

The software allows the visualization of models (or parts of models) using an internal tool. Currently, this tool allows to import different layout formats, but here we will be focused only in the Cell Designer tool to support the definition of the layout.

This operation is available in the option **Import Layout** from the “File/ Import” menu as shown in Figure 51.

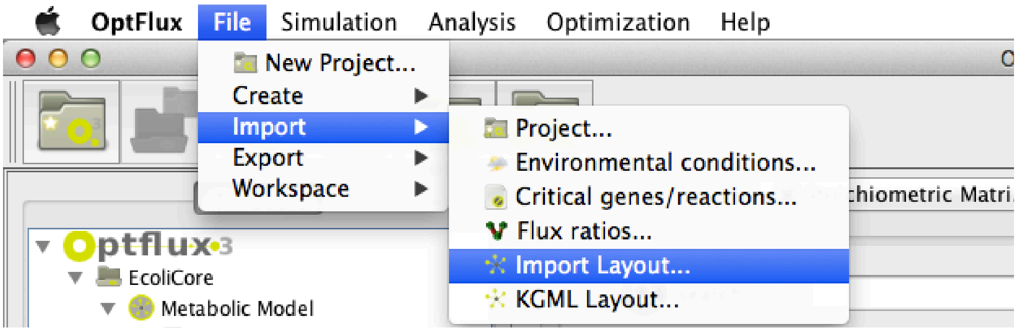

Figure 51: Visualization tool - menu option

To illustrate this feature, please import the cell designer layout, from the same file that you have used to create the *Saccharomyces cerevisiae* model, referred on section 10, associate it to the corresponding model project, and select the "CellDesigner" item in the "Reader' combo box (Figure 52).'

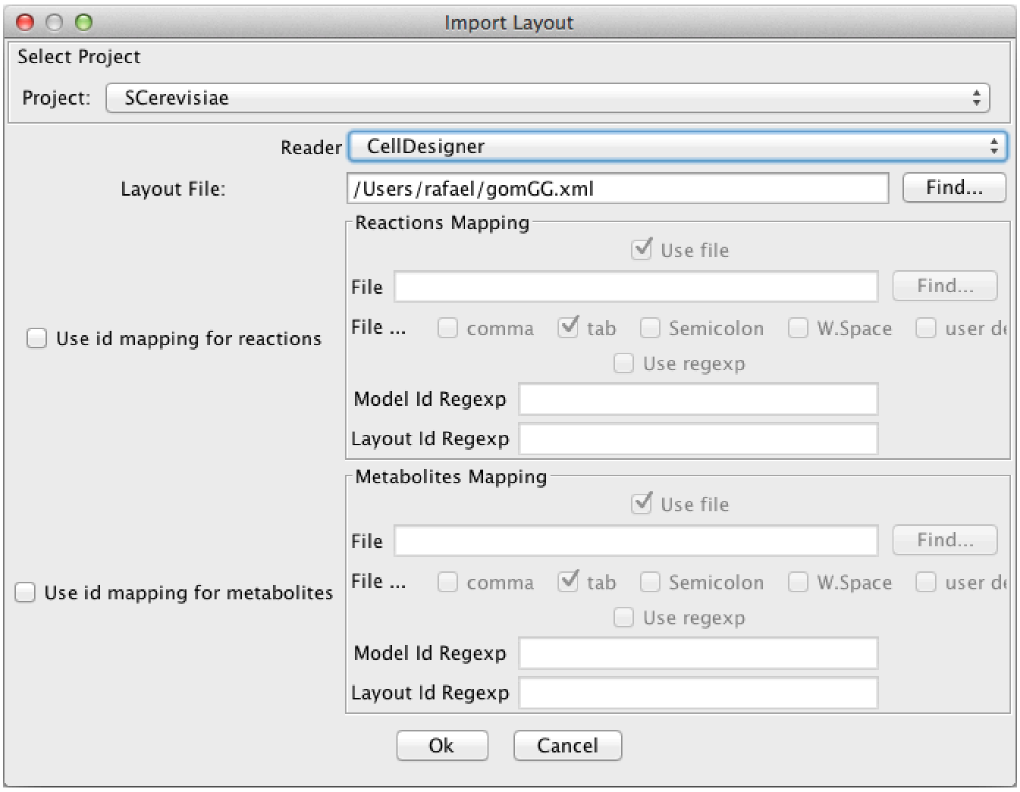

Figure 52: Visualization tool - operation dialogue

A new item is added to the clipboard in the “CellDesigner Layouts” list, under the “Project elements”. If you click on this object, a new interface will be presented in the visualization area (Figure 52). In this interface, two panels are shown, the left one showing the graphical representation of the model (using the layout created in Cell Designer) and the right one showing some filters that can be used to configure what is shown and, additionally, showing some information on the items selected from the graph (both reactions and metabolites).

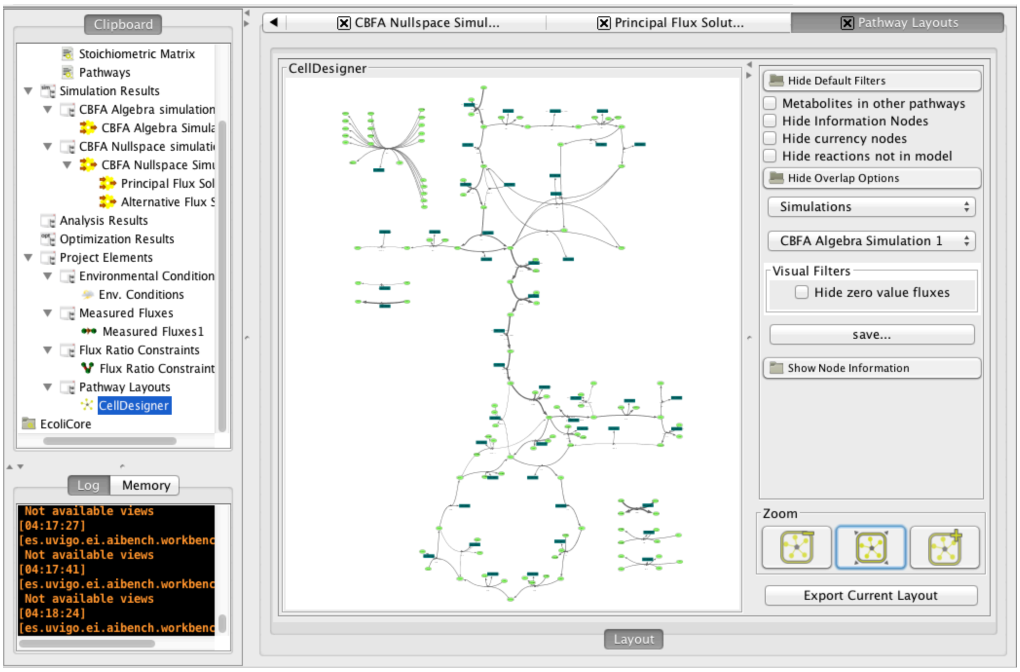

Figure 53: Visualization tool simulation flux distribution view

It is also possible to show the results of flux analysis methods, that have been performed, overlapped in the graph. In this case, you need to define which flux distribution to use, selecting a solution from the list box under “Flux Distribution”.

More details on this visualization tool and supported formats can be found in the how to’s of the OptFlux platform in the main web site.

References

[Förster et al., 2002] Förster, J., Gombert, A. K., and Nielsen, J. (2002). A functional genomics approach using metabolomics and in silico pathway analysis. *Biotechnology and Bioengineering*, 79(7):703–712.

[Orth et al., 2010] Orth, J. D., Fleming, R. M. T., and Palsson, B. O. (2010). Reconstruction and use of microbial metabolic networks: the core Escherichia coli metabolic model as an educational guide.
